# Turkish inappropriate medication use in the elderly (TIME) criteria to improve prescribing in older adults: TIME-to-STOP/TIME-to-START

**DOI:** 10.1007/s41999-020-00297-z

**Published:** 2020-03-05

**Authors:** Gulistan Bahat, Birkan Ilhan, Tugba Erdogan, Meltem Halil, Sumru Savas, Zekeriya Ulger, Filiz Akyuz, Ahmet Kaya Bilge, Sibel Cakir, Kutay Demirkan, Mustafa Erelel, Kerim Guler, Hasmet Hanagasi, Belgin Izgi, Ates Kadioglu, Ayse Karan, Isin Baral Kulaksizoglu, Ali Mert, Savas Ozturk, Ilhan Satman, Mehmet Sukru Sever, Tufan Tukek, Yagiz Uresin, Onay Yalcin, Nilufer Yesilot, Meryem Merve Oren, Mehmet Akif Karan

**Affiliations:** 1 Division of Geriatrics, Department of Internal Medicine, Istanbul Medical School, Istanbul University, Capa, 34390 Istanbul, Turkey; 2 Division of Geriatric Medicine, Department of Internal Medicine, Hacettepe University Faculty of Medicine, Ankara, Turkey; 3 Division of Geriatrics, Department of Internal Medicine, Ege University Faculty of Medicine, Izmir, Turkey; 4 Department of Internal Medicine, Kirikkale University Medical School, Kirikkale, Turkey; 5 Division of Gastroenterology, Department of Internal Medicine, Istanbul University Istanbul Medical School, Istanbul, Turkey; 6 Department of Cardiology, Istanbul University Istanbul Medical School, Istanbul, Turkey; 7 Department of Psychiatry, Istanbul University Istanbul Medical School, Istanbul, Turkey; 8 Department of Clinical Pharmacy, Hacettepe University Faculty of Pharmacy, Ankara, Turkey; 9 Department of Pulmonary Medicine, Istanbul University Istanbul Medical School, Istanbul, Turkey; 10 Department of Internal Medicine, Istanbul University Istanbul Medical School, Istanbul, Turkey; 11 Department of Neurology, Istanbul University Istanbul Medical School, Istanbul, Turkey; 12 Department of Ophthalmology, Istanbul University Istanbul Medical School, Istanbul, Turkey; 13 Department of Urology, Istanbul University Istanbul Medical School, Istanbul, Turkey; 14 Department of Physical Therapy and Rehabilitation, Istanbul University Istanbul Medical School, Istanbul, Turkey; 15 Infectious Diseases and Clinical Microbiology, Faculty of Medicine, Istanbul Medipol University, Istanbul, Turkey; 16 Department of Nephrology, Haseki Training and Research Hospital, Istanbul, Turkey; 17 Division of Endocrinology, Department of Internal Medicine, Istanbul University Istanbul Medical School, Istanbul, Turkey; 18 Division of Nephrology, Department of Internal Medicine, Istanbul University Istanbul Medical School, Istanbul, Turkey; 19 Department of Pharmacology, Istanbul University Istanbul Medical School, Istanbul, Turkey; 20 Department of Obstetrics and Gynecology, Istanbul University Istanbul Medical School, Istanbul, Turkey; 21 Department of Public Health, Istanbul University Istanbul Medical School, Istanbul, Turkey

**Keywords:** TIME criteria, Inappropriate medication use, Polypharmacy, Prescribing, Screening, Elderly

## Abstract

**Purpose:**

To improve prescribing in older adults, criterion sets have been introduced from different countries. While current criterion sets are useful to some extent, they do not meet the need in some European countries. Turkish inappropriate medication use in the elderly (TIME) criteria was planned to meet this need.

**Methods:**

In phase 1, the user friendly sets: STOPP/START version2 and CRIME criteria were combined. National experts composed of geriatricians and non-geriatricians were invited to review and comment. In phase 2, thorough literature review was performed and reference-based revisions, omissions, and additions were made. Explanatory additions were added to some criteria to improve application in practice. In phase 3, all working group members reviewed the criteria/explanations and agreed on the final content.

**Results:**

Phase 1 was performed by 49 expert academicians between May and October 2016. Phase 2 was performed by 23 working group academicians between October 2016 and November 2018 and included face-to-face interviews between at least two geriatrician members and one criterion-related specialist. Phase 3 was completed between November 2018–March 2019 with review and approval of all criteria by working group academicians. As a result, 55 criteria were added, 17 criteria were removed, and 60 criteria were modified from the first draft. A total of 153 TIME criteria composed of 112 TIME-to-STOP and 41 TIME-to-START criteria were introduced.

**Conclusion:**

TIME criteria is an update screening tool that differs from the current useful tools by the interactive study of experts from geriatrics and non-geriatrics, inclusion of practical explanations for some criteria and by its eastern European origin. TIME study respectfully acknowledges its roots from STOPP/START and CRIME criteria. Studies are needed whether it would lead improvements in older adults’ health.

**Electronic supplementary material:**

The online version of this article (10.1007/s41999-020-00297-z) contains supplementary material, which is available to authorized users.

## Key summary points

**Aim:**

To meet the current need in different European countries for improving prescribing in older adults, we aimed to create an update screening tool getting origin from the two user friendly criterion sets: the STOPP/STARTv2 criteria and CRIME criteria.

**Findings:**

Based on thorough literature review, 55 criteria were added, 17 criteria were removed, and 60 criteria were modified. As a result, 153 TIME criteria composed of 112 TIME-to-STOP and 41 TIME-to-START criteria were introduced.

**Message:**

TIME criterion set is an update screening tool reported from Eastern Europe that included experts from geriatrics and other specialties frequently giving care to older adults and some additional practical explanations for clinical use.

**Electronic supplementary material:**

The online version of this article (10.1007/s41999-020-00297-z) contains supplementary material, which is available to authorized users.

## Introduction

Aging is often associated with multiple comorbidities, geriatric syndromes, and the consequent multiple drug use and polypharmacy. The older adults are more prone to adverse effects of medications due to their decreased physiological reserves, the negative effects of the age-related comorbidities, increased vulnerability for geriatric syndromes, and higher number of drug use [1]. As the population ages, the adverse effects of drugs in the older adults emerge as a public health problem [2]. In geriatric perspective, the possibility of an adverse drug event should always be kept in mind when evaluating an older adult. Any new symptom should be considered medication side effect until proven otherwise [3]. However, on the other hand, older individuals are also at risk for denial of clinically indicated medications without a plausible reason. Accordingly, potentially inappropriate prescribing (PIP) includes over-, mis-, and also under-prescribing [4]. The significant negative medical, economic, and social consequences of inappropriate medication use (IMU) are very well known [2]. Thus, optimization of the drug therapy is an essential yet challenging component of management of older adults.

Explicit (criteria based) screening tools and implicit (judgment based) evaluation methods are among the tools developed to help in management of drug therapy in the older adults. Explicit tools are algorithmic approaches that include lists of drugs to avoid or specific indicators of inappropriate medication use [2, 5, 6]. They are helpful to provide knowledge and guidance on optimal medication use. Implicit approaches evaluate the patient with a much broader concept including the research data, clinical circumstances, and patient/family preferences [7, 8]. Thus, while the implicit evaluations offer the most appropriate assessment in terms of detecting IMU, they require much more time, background knowledge, and the judgment, which is hard to standardize. Therefore, explicit tools have been more widely studied to guide clinicians in the management of IMU. Studies using these methods showed high prevalence of PIPs and suggested that early detection may prevent negative outcomes and improve health outcomes in older adults [9–11]. The most commonly used criteria sets are the Beers criteria and the Screening Tool of Older Persons’ potentially inappropriate Prescriptions/Screening Tool to Alert to Right Treatment (STOPP/START) tools [12, 13], while the CRIME criterion set also comes forward with its recommendations focusing on medication therapy specifically in the older complex patients [14].

The explicit screening tools are needed to be updated periodically because of emerging scientific evidence. The Beers criteria from US have been updated on 2019 and the STOPP/START criteria from Europe have been updated on 2015 [5, 6]. PIP profile, prescription habits, and also existing medicines in the local market vary between the US and Europe and also within different parts of the Europe [10, 15]. Hereby, there are PIP lists developed by different European countries such as PRISCUS and FORTA in Germany [16, 17]; GheOP^3^S tool in Belgium [18]; and other specific lists from France and Norway [19, 20] but none is present from Eastern Europe. As an example of Eastern European countries, Turkey has significantly high-documented PIP prevalence as between 33.3 and 41.2% in older adults detected by implementation of STOPP and Beers criteria sets [15, 21]. Therefore, an update explicit screening tool for PIP seems reasonable for use both in primary care and research from Eastern Europe. Another point is that, the to date explicit tools have been developed by the groups involving geriatricians and specialists on pharmacology with few of them involving other major specialists involved in care of older adults. Maybe due to this reason, their use in researches and practice seems to be limited mostly to geriatricians. In addition, during application in clinical practice, non-specialist physicians ask for some explanations for some criteria related to the drugs/conditions they are less familiar with. Given this background, we aimed to produce Turkish inappropriate medication use in the elderly (TIME) criterion set as an update screening tool for PIP from Eastern Europe, including non-geriatrician specialists in addition to the geriatricians and explanations to aid in clinical practice when deemed necessary.

## Materials and methods

TIME study was performed under the leadership of Rational Drug Use Working Group of the Turkish Academic Geriatrics Society. It is a consensus study involving the TIME study group: the expert group and the working group. Study was performed in three phases (Table 1).

**Table 1:** The methodology involving three phases of the TIME study

Phase 1
Creation of Draft 1 TIME criteria by combination of STOPP/STARTv2 and CRIME criteria
Review of Draft 1 TIME criteria for their comments and suggestions by the expert group through e-mail correspondences
Phase 2
Creation of Draft 2 TIME criteria by inclusion of all expert comments and suggestions
Literature search
Face-to-face meetings within the working group to review all the draft 2 criterion
Creation of Draft 3 TIME criteria
Phase 3
Literature search for recent evidence
Review of all the Draft 3 TIME criteria by all working group members through e-mail correspondences
Creation of final TIME criteria set

### Phase 1

In phase 1, the first draft of TIME criterion set (Draft 1) was established by combination of STOPP/STARTv2 and CRIME criterion sets. As the basis of TIME criterion set, STOPP/STARTv2 and CRIME sets were chosen due to their user friendly structure and European origin for the both sets, recognition of dual nature of PIPs for the STOPP/START set and its specific focus on complex older adults for the CRIME set. For constituting and grouping the TIME criteria, we modelled STOPP/START set. The criteria were grouped as the TIME-to-STOP criteria for the criteria involving drugs that are often inappropriately over-prescribed in older adults and the TIME-to-START criteria for the criteria involving the drugs clinically indicated but frequently under-prescribed in the older adults.

As the second step, the TIME expert group, composed of 49 academicians from geriatrics and other specialties frequently taking care of older adults, was constituted. The composition of the expert group was as follows: 17 members from geriatrics; 4 members from psychiatry; 3 members each from neurology, cardiology, gastroenterology, general internal medicine and pharmacology; 2 members each from endocrinology, nephrology, physiatry, urology; 1 member each from pulmonology, infectious diseases, gynecology, ophthalmology and clinical pharmacology. These experts were identified among the recognized and experienced academicians giving care to older adults in their usual practice. Then, the expert group was asked by e-mail to review and comment on the Draft 1 criteria by free text within the framework of current literature, their clinical practices and expert opinions. All the suggestions and comments were requested by accompanying reasons and references. Phase 1 was completed after receipt of the suggestions/comments from all of the members of the expert group. Answers were anonymized for analysis.

### Phase 2

In phase 2, Draft 2 TIME criterion set was established by integration of all expert comments and suggestions to the Draft 1. Working group was created from the 23 academicians from expert group members that could interact closely in the second phase and additionally included 2 fellows of geriatrics (BI, TE) and 1 statistician consultant (MO). In this phase, the PubMed, Embase, and Cochrane Library databases were searched for the current evidence of the criteria, the comments, and the suggestions. The keywords related to each proposed criterion were entered in the all three databases. Search terms for each criterion included individual drugs, drug classes, specific conditions, and combinations thereof. For TIME-to-STOP, each search specially focused on “adverse drug events” and “adverse drug reactions.” Search filtered for human participants in either English or Turkish language. The relevant articles were identified in the following categories: systematic reviews, reviews, randomised controlled trials (RCTs), and guidelines. Database search and review of selected articles were performed by four members of the working group (the principal author, the fellows of geriatrics, and statistician member) to assess their suitability as a supporting evidence. This procedure was supervised and directed by the principal author. We also examined Lexicomp^®^ drug information for specific drug informations. The references of the criteria from STOPP/STARTv2 and CRIME criterion sets were also considered.

Afterwards, two geriatrician members and the criterion-related specialty-specific working group members worked at face-to-face meetings to review the Draft 2 criteria in view of the references. Among suggestions and comments at Draft 2 criterion set, additional criteria were generated if the comments had sound evidence. Existing criteria were removed if they currently lacked evidence, if they were already been included within a more comprehensive added criteria, if they were on medications not commonly used in general practice (but only in limited specialty-specific practices) or assessed as having concerns in practical application. If deemed necessary, the remaining criteria were modified as follows: changes according to the recent literature evidence, addition of explanations, inclusion of other problematic medications and the doses according to the availability in the market, converting units of measurements to the practically used units, and combining criteria for ease of understanding. Explanations, which were judged helpful and added to some criteria to aid in clinical use, were composed of alternative treatment options with detailed recommendations for the proposed treatment, additional risk factors and cautions, some specific characteristics within the same group of drugs, frequent wrong application examples with approaches to prevent them. The working group members were given chance to add further references to the above supplied references from the recently published textbooks, official publications of the medical societies, the Food and Drug Administration (FDA)’s online sources, the evidence-based clinical decision support resource UpToDate^®^, and evidence-based references published in a peer-reviewed journal, to reinforce the evidence identified from the above search system. In this process, the methodologic quality of each study, its relevance to older adults, and its concordance with the desired evidence were considered. At the end of Phase 2, Draft 3 TIME criteria were established.

### Phase 3

At the beginning of the third phase, the database search was repeated to reflect the current evidence emerged between Phase 2 and Phase 3 and relevant publications were identified as described above. Draft 3 TIME criteria and references as a whole were sent to the all TIME working group academicians by e-mail to review the Draft 3 TIME criteria. At this stage, working group academicians paid particular attention to review all the criteria specifically related to their field; however, also they reviewed the remaining TIME criteria. The working group academicians were let available to propose additional criteria or change at the Draft 3 criteria based on the recent evidences between Phase 2 and Phase 3**.** The same search strategy with Phase 2 was conducted in Phase 3 for the articles published in the time period between Phase 2 and Phase 3. This was the final revision phase for the TIME criteria. At the end of this phase, the TIME criteria were finalized with the approval of all working group members. Before submission of the manuscript, online references were checked with regard to current availability.

The construction process of TIME criteria is summarised in Table 1.

The study protocol was approved by the Istanbul University, Istanbul Medical School ethics committee and conducted according to the guidelines laid down in the Declaration of Helsinki.

## Results

The study was completed in 3 years period between May 2016 and March 2019. Draft 1 TIME criteria were composed of a total of 133 criteria which consisted 114 criteria from STOPP/START version2 (80 STOPP criteria, 34 START criteria) and 19 criteria from CRIME criteria. When grouped into the preliminary TIME-to-STOP and TIME-to-START criteria groups, Draft 1 TIME criteria were composed of 99 preliminary TIME-to-STOP and 34 preliminary TIME-to-START criteria. The e-mails including Draft 1 TIME criteria were sent on May 2016 to the expert group members and phase 1 was completed on October 2016 upon completion of the reviews from all of the group members on Draft 1. Phase 2 started on October 2016 by integration of all expert comments and suggestions to the Draft 1 criteria with establishment of the resultant Draft 2 criteria. Literature search was performed, and relevant references were selected. Two hundred and eighty-eight references were selected for TIME-to-STOP criteria and 173 for TIME-to-START criteria. All the relevant reference articles were provided to the working group online. After the evaluation with the literature search and face-to-face meetings by the working group in phase 2, Draft 3 criteria were generated by addition of 50 new criteria that had supported published evidence to warrant inclusion, removal of 17 criteria, and modification of 60 criteria. As a new section of criteria, “Supplements” section was generated including some dietary supplements at TIME-to-STOP criteria and oral nutritional supplements at TIME-to-START criteria. Phase 2 was completed by generation of Draft 3 TIME criteria on November 2018. Phase 3 started on November 2018 with literature search to reflect the recent evidence accumulated in 2 years period of the second phase. Draft 3 TIME criteria and all references were sent to the 23 working group members for review of the full content and propose changes if needed. At phase 3, five additional new criteria (three were TIME-to-STOP criteria; two were TIME-to-START criteria) that had supported published evidence were added and some minor changes in expressions were made with establishment of final TIME criteria. Thirty-three new TIME-to-STOP references and 28 TIME-to-START references were added in Phase 3. The full reviews were completed on March 2019. As a result, the final TIME criteria included a total of 153 criteria composed of 112 TIME-to-STOP and 41 TIME-to-START criteria. The details of the changes made on upon Draft 1 criteria through the development process of the final TIME criteria are outlined at Table 2. The total of 55 added criteria and 17 removed criteria by the TIME study is given in Online Resource 1 and Online Resource 2, respectively. The final list of TIME-to-STOP and TIME-to-START criteria is given in Online Resource 3 and Online Resource 4, the final list of TIME-to-STOP and TIME-to-START criteria with full list of references and accompanying explanations added to some criteria is given in Online Resource 5 and Online Resource 6, respectively.

**Table 2:** The development process of the final TIME criteria: summary of changes applied to the Draft 1 criteria

Number of added criteria: 55^a^
43 criteria created by TIME study group
30 TIME-to-STOP criteria
12 TIME-to-START criteria
12 criteria from the Beers criteria
Number of removed criteria: 17^b^
5 criteria from CRIME criteria
12 criteria from STOPP/ START version2
8 criteria from STOPP criteria
4 criteria from START criteria
Number of criteria adapted/modified by the TIME study group: 60
Number of criteria with no changes: 35

^a^Added 55 criteria are given in Online Resource 1

^b^Removed 17 criteria are given in Online Resource 2

## Discussion

In this study, we have constituted an updated screening tool: the TIME criteria, getting origin from the two user friendly criterion sets: the STOPP/STARTv2 criteria and CRIME criteria. We adapted STOPP/STARTv2 and CRIME criteria for their use in Turkey; evaluated and—if considered necessary—updated the criteria according to the most recent data, added new criteria accounting for the recent data and the local practices, and—last but not the least—we added practical explanations accompanying some criteria if deemed necessary.

Explicit tools to detect IMU are helpful for the clinicians taking care of older adults as they provide pragmatic, condensed knowledge and guidance in the management of medications. Fronting the ageing world, explicit tools are needed and welcomed by practitioners as potential aids to improve medication use among older adults. Beers criterion set has a unique place among the explicit tools as it represents the first attempt that recognizes and focuses on IMU systematically. While it has been helpful and is the most cited criteria set, it has some drawbacks. As it originated from US, it contains significant number of drugs that are absent from most European markets; its structure is complex precluding its use in clinical practice, and it fails to consider indicated but under-used drugs in the older adults [22]. Accordingly, it remained essentially a research tool rather than a practical aid in clinical practice of the older adults [22]. STOPP and START criterion set has been created to cover these problems and been well accepted. It has been successfully applied for both research and practical clinical purposes in several countries not only in Europe, but also in Asia, Australia, North and South America [6]. Nevertheless, it originates from a distinct European country i.e. the Ireland and, therefore, attempts that aim to adapt STOPP/START tool to the different countries [23–26] or to create new explicit tools [16, 19, 20] have been made. To our knowledge, none of these attempts were from Eastern Europe that Turkey belongs to. As an example of Eastern European countries, Turkey has significantly high-documented PIP prevalence [15, 21] that was detected at a higher extend with STOPP/STARTv2 criteria when compared to the Beers criteria [15]. Also, we recently showed that there were as much as STOPPv2 identified PIPs by clinical evaluation which was not detected by STOPPv2 in a significant percent of our older patients [27]. CRIME criteria have, on the other hand, a special feature, as it developed recommendations for the older complex patients. Hence, an updated screening tool, taking its origins from STOPP/START and CRIME criteria and considering the most recent data, and local practices/factors into account was judged to be needed and useful for our researches and practices. As a result, TIME study completed the three phases with 153 criteria developed by the addition of 55 new criteria, removal of 17 criteria and modification/adaptation of 60 criteria from the first 133 criteria draft based on the STOPP/STARTv2 and CRIME tools.

Considering its origination widely from STOPP/START criteria and the appreciated easy and practical use of STOPP/START tool, we organized TIME criteria as TIME-to-STOP and TIME-to-START categories. One of the important progresses was the addition of new section as supplements. Use of herbal or dietary supplements by older adults is a common and increasing trend involving more than 50% of older adults [28]. Supplements may have significant interaction with prescribed drug therapies leading to adverse events [29] and is recognized as a significant problem in the older adults [30]. Some common significant examples with problematic adverse effects have been presented in the TIME-to-STOP supplement section. This section may increase inquiry about use of herbal supplements and decrease their potentially inappropriate use. On the other hand, the beneficial use of oral nutritional supplements as food for special medical purposes has been well documented particularly in older adults with malnutrition e.g. during hospitalization. The use of nutritional supplements is regarded as medical therapy [31], is being reimbursed in some countries including Turkey and, therefore, has been presented in TIME-to-START section. We suggest this section could enhance integration of nutritional care for the older adults, which would be an important forward step in well care of older adults.

There are some other distinctive features of TIME criteria. Concerning the involved developing physicians, the explicit tools included mostly geriatricians and specialists on pharmacology or clinical pharmacy [5, 6, 32, 33], some included additional general practitioners [20, 34, 35] and very few included the other specialties that are frequently involved in care of older adults (e.g. neurology, psychiatry) [16, 36]. To our knowledge, involvement of the major specialties other than above, e.g. cardiologists, pulmonologists, nephrologists, urologists, etc., was lacking. This practice seems limiting use of IMU criterion sets mostly to geriatricians, maybe in part due to being non-informed and unaware for. Furthermore, these physicians frequently take care of older adults in their clinical practice and, therefore, their contribution with accompanying geriatricians could improve the benefit of these tools. Therefore, TIME study was performed by involvement of the physicians commonly prescribing for the older adults. To ensure and preserve the geriatric view, two geriatrician members unexceptionally worked with the criterion-related specialty-specific working group members at face-to-face meetings. It has been constituted with participation of quite high number, 49, expert academicians from geriatrics and non-geriatrics specialties involved in medical care of older adults i.e., psychiatry, neurology, cardiology, general internal medicine, endocrinology, nephrology, physiatry, urology, gynecology, gastroenterology, pulmonology, infectious diseases, ophthalmology, and also pharmacology/clinical pharmacology We suggest that this approach is an important feature of the TIME study.

The IMU tools are developed not only for use of geriatricians, but also for the general practitioners and other specialists giving care for older adults. It is not uncommon that during application in clinical practice, non-specialist physicians ask for some explanations for some criteria related to the drugs/conditions as they are less familiar with. As another significant feature of TIME criteria, explanations, aimed to aid their use in practice were added. They were composed of alternative treatment options with detailed recommendations for the proposed treatment, additional risk factors and cautions, some specific characteristics within the same group of drugs, frequent wrong application examples with approaches to prevent them. As an example, while acknowledging inappropriate additional use of aspirin or clopidogrel in patients already on oral anticoagulant treatment in lack of specific indications for the add on use, indications for the add on use and the frequently applied inappropriate add on use practices were given in explanation to help the practicing clinicians. This approach was partly applied previously with GheOP^3^S tool which included the alternative treatment approaches when present and been appreciated as helpful [18].

We suggest and hope that TIME criteria would be helpful not only locally, but also globally as it is performed by global updated literature search aiming to attain the best current knowledge. As demanded, TIME criteria considered the local needs and cultural background during its development such as addition of the vaccination criteria for meningococcal vaccine in older adults who will pilgrimage to Mecca. However, it is important to underline that, as TIME criteria readily includes the aspects included by the well-accepted central European tools and update thorough literature review, it could be regarded as a widened set for use of older adults from additional different cultural backgrounds rather than restricted to use by them.

We presented the results of TIME study with completed third phase in this manuscript. Our next task is completion of an additional fourth phase composed of the international Delphi validation and it is currently underway. Eleven internationally recognized panellist on geriatric pharmacotherapy has been involved in this validation phase to come up with internationally validated TIME criteria set with Delphi consensus. In this planned validation, aforementioned explanations accompanying some of the criteria were not involved due to difficulty in integration and limited comprehensibility for the panellists by online interaction. The readers would be referred to the present study for the explanations.

In conclusion, we presented TIME criterion set to detect IMU and improve use of medications in older adults as a consensus study developed by the local experts from Eastern Europe by respectfully acknowledging its roots from STOPP/START and CRIME criteria sets. While local in preparation, it included internationally recognised experts and global literature search. We suggest that it could be helpful for all the physicians practicing with the older adults. We look forward to reactions from the medical community and results and comprehension related to its use in future practices.

## Electronic supplementary material

Below is the link to the electronic supplementary material.

Supplementary file1 (DOCX 26 kb)

Supplementary file2 (DOCX 23 kb)

Supplementary file3 (DOCX 40 kb)

Supplementary file4 (DOCX 32 kb)

Supplementary file5 (DOCX 146 kb)

Supplementary file6 (DOCX 104 kb)
