# Supplementary material for: Turkish inappropriate medication use in the elderly (TIME) criteria to improve prescribing in older adults: TIME-to-STOP/TIME-to-START
Source: Eur Geriatr Med. 2020 Mar 5;11(3):491–8. doi: 10.1007/s41999-020-00297-z (PMC7280176; doi:10.1007/s41999-020-00297-z)
Supplement: Supplementary file 1 — Supplementary file1 (DOCX 26 kb) [file 41999_2020_297_MOESM1_ESM.docx]

**Turkish Inappropriate Medication Use in the Elderly (TIME) criteria to improve prescribing in older adults: TIME to STOP/TIME to START**

**Journal name:** European Geriatric Medicine

**Gulistan Bahat**^1^**, Birkan Ilhan**^1^**,** Tugba Erdogan^1^**, Meltem Halil**^2^**, Sumru Savas**^3^**, Zekeriya Ulger**^4^**, Filiz Akyuz**^5^**, Ahmet Kaya Bilge**^6^**, Sibel Cakir**^7^**, Kutay Demirkan** ^8^**, Mustafa Erelel^9^, Kerim Guler**^10^**, Hasmet Hanagasi**^11^**, Belgin Izgi**^12^**, Ates Kadioglu**^13^**, Ayse Karan**^14^**, Isin Baral Kulaksizoglu**^7^**, Ali Mert**^15^**, Savas Ozturk**^16^**, Ilhan Satman**^17^**, Mehmet Sukru Sever**^18^**, Tufan Tukek**^10^**, Yagiz Uresin**^19^**, Onay Yalcin**^20^**, Nilufer Yesilot**^11^**, Meryem Merve Oren^21^, Mehmet Akif Karan**^1^

^1^*Istanbul University, Istanbul Medical School, Department of Internal Medicine, Division of Geriatrics, Istanbul, Turkey*

^2^ *Hacettepe University Faculty of Medicine, Department of Internal Medicine, Division of Geriatric Medicine, Ankara, Turkey.*

^3^ *Ege University Faculty of Medicine, Department of Internal Medicine, Division of Geriatrics, Izmir, Turkey.*

^4^ *Kirikkale University Medical School, Department of Internal Medicine, Kirikkale, Turkey*

^5^*Istanbul University Istanbul Medical School, Department of Internal Medicine, Division of Gastroenterology, Istanbul, Turkey*

^6^*Istanbul University Istanbul Medical School, Department of Cardiology, Istanbul, Turkey*

^7^*Istanbul University Istanbul Medical School, Department of Psychiatry, Istanbul, Turkey*

*^8^Hacettepe University Faculty of Pharmacy, Department of Clinical Pharmacy, Ankara, Turkey.*

^9^ *Istanbul University Istanbul Medical School, Department of Pulmonary Medicine, Istanbul, Turkey*

^10^*Istanbul University Istanbul Medical School, Department of Internal Medicine, Istanbul, Turkey*

^11^*Istanbul University Istanbul Medical School, Department of Neurology, Istanbul, Turkey*

*^12^Istanbul University Istanbul Medical School, Department of Ophthalmology, Istanbul, Turkey*

^13^*Istanbul University Istanbul Medical School, Department of Urology, Istanbul, Turkey*

^14^*Istanbul University Istanbul Medical School, Department of Physical Therapy and Rehabilitation, Istanbul, Turkey*

^15^*Istanbul Medipol University, Infectious Diseases and Clinical Microbiology, Faculty of Medicine, Istanbul, Turkey*

^16^*Haseki Training and Research Hospital, Department of Nephrology, Istanbul, Turkey*

^17^*Istanbul University Istanbul Medical School, Department of Internal Medicine, Division of Endocrinology, Istanbul, Turkey*

^18^*Istanbul University Istanbul Medical School, Department of Internal Medicine, Division of Nephrology, Istanbul, Turkey*

^19^*Istanbul University Istanbul Medical School, Department of Pharmacology, Istanbul, Turkey*

^20^*Istanbul University Istanbul Medical School, Department of Obstetrics and Gynecology, Istanbul, Turkey*

^21^*Istanbul University Istanbul Medical School, Department of Public Health, Istanbul, Turkey*

**Corresponding author:** Gulistan Bahat (**For Reprint**)

**Address:** Istanbul University, Istanbul Medical School, Department of Internal Medicine, Division of Geriatrics, Capa, 34390, Istanbul, Turkey

**Telephone:** + 90 212 414 20 00-33204

**Fax:** + 90 212 532 42 08

**E-mail address:**gbahatozturk@yahoo.com

**Added criteria by the TIME study group (n=55)**

**43 criteria developed by the TIME study group**

#### *TIME to STOP criteria (n=31)*

#### 1.Use of beta-blockers as first line treatment for essential hypertension in lack of specific beta-blocker indication (increased risk of heart block, fatigue, sexual dysfunction and low activity in stroke protection, additionally, β-adrenergic receptor function decreases with aging).

#### 2. Fludrocortisone for the treatment of orthostatic hypotension without the exclusion of secondary factors and use of non-pharmacological approaches.

#### 3. Potassium-sparing drugs (aldosterone antagonists, triamterene, amiloride, ACEI, ARB) in patients with eGFR<30 ml/min/1.73m2 and whose serum potassium level cannot be closely monitored (risk of hyperkalemia).

#### 4.Prasugrel in patients aged 75 years or older or had TIA/ stroke.

#### 5. Allopurinol for asymptomatic hyperuricemia (those without gout or nephrolithiasis) (no evidence for benefit, risk of side effects with the use of xanthine oxidase inhibitors) (there is no evidence that treatment reduces cardiovascular risk or gout).

#### 6. Paroxetine, fluoxetine and fluvoxamine as the first line treatment among SSRIs (due to high anticholinergic effect of paroxetine, long half-life of fluoxetine, frequent drug interaction with fluoxetine and fluvoxamine).

#### 7. SNRIs in patients with uncontrolled hypertension.

#### 8. Anticholinergic agents for the treatment of Parkinson's disease (increased risk of side effects, safer and more effective drugs available).

#### 9. Continuous and long-term use of betahistine, trimetazidine, dimenhydrinate in the treatment of vertigo (no evidence-based beneficial effect).

#### 10. Cinnarizine use (extrapyramidal side effects, limited use).

#### 11. Piracetam except for myoclonic convulsion therapy (with no proven clinical efficacy, cost burden and side effect potential).

#### 12. Carbamazepine, phenytoin, phenobarbital or valproate for chronic treatment of epilepsy as first step therapy (negative effects on vitamin D, enzyme induction, risk of falls, also safer alternatives available).

#### 13. Antiepileptic treatment for seizure prophylaxis due to the presence of ischemic / hemorrhagic stroke in a patient without prior seizure.

#### 14. Citalopram >20 mg / day and escitalopram >10 mg / day  (risk of QTc elongation).

#### 15. Initiation of chronic aspirin or NSAID use without testing for H. pylori in patients with a history of peptic ulcer (complicated or uncomplicated, gastric or duodenal).

#### 16. PPIs for multiple drug use indication (no benefit, potential harm).

#### 17. Magnesium preparations as laxative or antacid if eGFR<30 ml/min/1.73m2 (risk of hypermagnesemia).

#### 18. Methotrexate if eGFR<30 ml/min/1.73m2.

#### 19. Initiation of osteoporosis treatment without excluding osteomalacia diagnosis.

#### 20. Conventional vitamin D at high intermittent doses (300,000 IU) as ‘maintenance’ vitamin D therapy (increased fall risk, no additional benefit on the musculoskeletal system).

#### 21. Active vitamin D (1-25 (OH)2 cholecalciferol) (calcitriol) or conventional  vitamin D (25 (OH) cholecalciferol)  in those with hyperphosphatemia and/ or hypercalcemia.

#### 22. Bisphosphonates if eGFR<30 ml/min/1.73m2 (increased risk of acute renal failure).

#### 23. Zoledronate, denosumab or teriparatide without monitoring serum calcium level and assuring adequate calcium/ vitamin D intake prior to the treatment.

#### 24. Antibiotic use in asymptomatic bacteriuria except during urological interventions that may damage the mucosa.

#### 25. Saxagliptin in patients with heart failure.

#### 26. Canagliflozin in patients with fracture/ recurrent urinary tract infection/ genitourinary infection/ severe peripheric artery disease/ lower extremity amputation due to diabetes.

#### 27. SGLT-2 inhibitors for glycemic regulation if eGFR<45 mL/min/1.73 m.

#### 28. Thyroid hormone in patients with subclinical hypothyroidism (TSH: 4-10 mIU/L; free T4: normal) (no additional benefit, risk of potential side effects such as atrial fibrillation, osteoporosis).

#### 29. Gingko biloba extract in patients with increased bleeding risk (use of anticoagulants, NSAIDs, history of significant bleeding).

#### 30. Systemic use of Hypericum perforatum (St. John’s Wort) in combination with antidepressants (the risk of serotonergic syndrome especially with SSRI) and drugs metabolized with cytochrome p450 (e.g. digoxin, theophylline, warfarin, carbamazepine, phenytoin, phenobarbital) (Hypericum perforatum (St. John’s Wort) activates cytochrome p450).

#### 31. Supplements with concurrent warfarin (high risk of interaction, increased risk of bleeding).

#### *TIME to START criteria (n=12)*

#### 1. Memantine for moderate-severe Alzheimer’s disease.

#### 2. Propranolol or primidone for essential tremor that interferes with functioning.

#### 3. Addition of a MAO-B inhibitor or COMT inhibitor to L-dopa treatment when on-off motor fluctuations start in idiopathic Parkinson's disease.

#### 4. Long-acting anti-resorptive treatment after discontinuation of at least two doses of denosumab (rebound increased BTMs, BMD loss and increased risk of vertebral fracture following denosumab discontinuation).

#### 5. Antiresorptive treatment after teriparatide treatment.

#### 6. Vaccination for herpes zoster (reduction in risk of shingles infection and post-herpetic neuralgia).

#### 7. Vaccination with Td (tetanus-diphtheria toxoid) every 10 years.

#### 8. Vaccination with meningococcal vaccine for patients who will pilgrimage to Mecca.

#### 9. ONS with MN or MNR if nutritional counseling/dietary supplementation are not sufficient to achieve nutritional goals.

#### 10. ONS for hospitalized older adults with MN or MNR (increases nutrient intake and body weight, reduces the risk of complications and readmissions).

#### 11. ONS for older adults with hip fractures in the postoperative period (regardless of nutritional status) (improves food intake and reduces the risk of complications).

#### 12. ONS with pressure ulcers to ensure adequate protein and energy intake targeting 1.2-2 g/kg/day protein, 30-35 kcal/kg/day energy.

**12 criteria adapted from the Beers criteria** [5]

1. Digoxin as first line treatment for atrial fibrillation.

2. Digoxin at a dose greater than 0.125 mg/day (toxicity risk).

3. Short-acting dipyridamole for antiplatelet-antiaggregant effect (orthostatic hypotension side effect and more effective agents).

4. Duloxetine if eGFR<30 ml/min/1.73m2 (increased GIS side effect).

5. Pregabalin and gabapentin without dose reduction if eGFR<30 ml/min/1.73m2.

6.Tramadol, neuroleptics/antipsychotics (clozapine, olanzapine, chlorpromazine, thioridazine), bupropion or maprotiline in epilepsy patients.

#### 7. Anticholinergic GIS antispasmodics (e.g. hyoscyamine) [increased anticholinergic side effect in older adults (dizziness, decreased cognitive function, blurred vision, arrhythmia, flatulence-constipation) and limited benefit].

#### 8. Meperidine in the treatment of pain (increased neurotoxicity, delirium risk, safer alternatives are available. Increased risk in especially patients with renal failure).

#### 9. Extended-release tramadol if eGFR<30 ml/min/1.73m^2^.

#### 10. Systemic skeletal muscle relaxant agents (thiocolchicoside, tizanidine, chlorzoxazone, carisoprodol, chlorfenese carbamate, cyclobenzaprine, metaxalone, methocarbamol and orphenadrine) for musculoskeletal pain (sedation, dizziness, dry mouth, constipation, cognitive impairment).

#### 11. Nitrofurantoin if eGFR<30 ml/min/1.73m2.

#### 12. Megestrol as an appetite enhancer (minimal effect on weight, prothrombotic side effect).

| **ABBREVIATIONS**  ACEI: Angiotensin converting enzyme inhibitors |
| --- |
| ARB: Angiotensin receptor blockers |
| BMD: Bone mineral density |
| BTMs: Bone Turnover Markers |
| COMT: Catechol-O-methyltransferase |
| eGFR: Estimated Glomerular Filtration Rate |
| GIS: Gastrointestinal system |
| MAO-B: Monoamine oxidase-B |
| MN: Malnutrition |
| MNR: Malnutrition risk |
| NSAID: Non steroidal anti inflammatory drug |
| ONS: Oral nutritional supplements |
| PPI: Proton pump inhibitors |
| QTc: Corrected QT Interval |
| SGLT-2: Sodium-glucose cotransporter-2 |
| SNRIs: Serotonin-norepinephrine reuptake inhibitors |
| SSRIs: Selective serotonin reuptake inhibitors |
| TIA: Transient ischemic attack |
| TSH: Thyroid stimulating hormone |
