# Supplementary material for: Turkish inappropriate medication use in the elderly (TIME) criteria to improve prescribing in older adults: TIME-to-STOP/TIME-to-START
Source: Eur Geriatr Med. 2020 Mar 5;11(3):491–8. doi: 10.1007/s41999-020-00297-z (PMC7280176; doi:10.1007/s41999-020-00297-z)
Supplement: Supplementary file 2 — Supplementary file2 (DOCX 23 kb) [file 41999_2020_297_MOESM2_ESM.docx]

**Turkish Inappropriate Medication Use in the Elderly (TIME) criteria to improve prescribing in older adults: TIME to STOP/TIME to START**

**Journal name:** European Geriatric Medicine

**Gulistan Bahat**^1^**, Birkan Ilhan**^1^**,** Tugba Erdogan^1^**, Meltem Halil**^2^**, Sumru Savas**^3^**, Zekeriya Ulger**^4^**, Filiz Akyuz**^5^**, Ahmet Kaya Bilge**^6^**, Sibel Cakir**^7^**, Kutay Demirkan** ^8^**, Mustafa Erelel^9^, Kerim Guler**^10^**, Hasmet Hanagasi**^11^**, Belgin Izgi**^12^**, Ates Kadioglu**^13^**, Ayse Karan**^14^**, Isin Baral Kulaksizoglu**^7^**, Ali Mert**^15^**, Savas Ozturk**^16^**, Ilhan Satman**^17^**, Mehmet Sukru Sever**^18^**, Tufan Tukek**^10^**, Yagiz Uresin**^19^**, Onay Yalcin**^20^**, Nilufer Yesilot**^11^**, Meryem Merve Oren^21^, Mehmet Akif Karan**^1^

^1^*Istanbul University, Istanbul Medical School, Department of Internal Medicine, Division of Geriatrics, Istanbul, Turkey*

^2^ *Hacettepe University Faculty of Medicine, Department of Internal Medicine, Division of Geriatric Medicine, Ankara, Turkey.*

^3^ *Ege University Faculty of Medicine, Department of Internal Medicine, Division of Geriatrics, Izmir, Turkey.*

^4^ *Kirikkale University Medical School, Department of Internal Medicine, Kirikkale, Turkey*

^5^*Istanbul University Istanbul Medical School, Department of Internal Medicine, Division of Gastroenterology, Istanbul, Turkey*

^6^*Istanbul University Istanbul Medical School, Department of Cardiology, Istanbul, Turkey*

^7^*Istanbul University Istanbul Medical School, Department of Psychiatry, Istanbul, Turkey*

*^8^Hacettepe University Faculty of Pharmacy, Department of Clinical Pharmacy, Ankara, Turkey.*

^9^ *Istanbul University Istanbul Medical School, Department of Pulmonary Medicine, Istanbul, Turkey*

*^10^Istanbul University Istanbul Medical School, Department of Internal Medicine, Istanbul, Turkey*

^11^*Istanbul University Istanbul Medical School, Department of Neurology, Istanbul, Turkey*

*^12^Istanbul University Istanbul Medical School, Department of Ophthalmology, Istanbul, Turkey*

^13^*Istanbul University Istanbul Medical School, Department of Urology, Istanbul, Turkey*

^14^*Istanbul University Istanbul Medical School, Department of Physical Therapy and Rehabilitation, Istanbul, Turkey*

^15^*Istanbul Medipol University, Infectious Diseases and Clinical Microbiology, Faculty of Medicine, Istanbul, Turkey*

^16^*Haseki Training and Research Hospital, Department of Nephrology, Istanbul, Turkey*

^17^*Istanbul University Istanbul Medical School, Department of Internal Medicine, Division of Endocrinology, Istanbul, Turkey*

^18^*Istanbul University Istanbul Medical School, Department of Internal Medicine, Division of Nephrology, Istanbul, Turkey*

^19^*Istanbul University Istanbul Medical School, Department of Pharmacology, Istanbul, Turkey*

^20^*Istanbul University Istanbul Medical School, Department of Obstetrics and Gynecology, Istanbul, Turkey*

^21^*Istanbul University Istanbul Medical School, Department of Public Health, Istanbul, Turkey*

**Corresponding author:** Gulistan Bahat (**For Reprint**)

**Address:** Istanbul University, Istanbul Medical School, Department of Internal Medicine, Division of Geriatrics, Capa, 34390, Istanbul, Turkey

**Telephone:** + 90 212 414 20 00-33204

**Fax:** + 90 212 532 42 08

**E-mail address:**gbahatozturk@yahoo.com

**Removed criteria by the TIME study group (n= 14)**

**Five criteria from the CRIME set†** [11]

#### 1.In patients with non-valvular atrial fibrillation and limited life expectancy (<6 months), the use of oral anticoagulants should be avoided.

#### 2. In non-valvular atrial fibrillation, the use of anticoagulants is not recommended in elderly patients with dementia if any of the following characteristics are present: unable to manage medications and living alone or high risk for falls.

#### 3. The use of statins for secondary prevention in older adults with limited life expectancy (<2 years) or advanced dementia is not recommended.

#### 4. In patients with dementia or cognitive impairment or functional limitation, use of more than three antihypertensive drugs should be avoided.

#### 5. In case of falls associated with orthostatic hypotension (or symptomatic orthostatic hypotension), the number of antihypertensive drugs should be reduced and concomitant use of multiple antihypertensive agents should be avoided.

**Twelve criteria from the STOPP/ START version2** [6] **criteria set**

***Eight STOPP criteria***

1. Any drug prescribed without an evidence-based clinical indication.*

2. Any drug prescribed beyond the recommended duration, where treatment duration is well defined.*

3. Any duplicate drug class prescription e.g. two concurrent NSAIDs, SSRIs, loop diuretics, ACE inhibitors, anticoagulants (optimization of monotherapy within a single drug class should be observed prior to considering a new agent).*

4.Amiodarone as first-line antiarrhythmic therapy in supraventricular tachyarrhythmias (higher risk of side-effects than beta-blockers, digoxin, verapamil or diltiazem)^‡^

5. Phenothiazines as first-line treatment, since safer and more efficacious alternatives exist (phenothiazines are sedative, have significant anti-muscarinic toxicity in older people, with the exception of prochlorperazine for nausea/vomiting/vertigo, chlorpromazine for relief of persistent hiccoughs and levomepromazine as an anti-emetic in palliative care ) ^‡^

6. Oral elemental iron doses greater than 200 mg daily (e.g. ferrous fumarate> 600 mg/day, ferrous sulphate> 600 mg/day, ferrous gluconate> 1800 mg/day; no evidence of enhanced iron absorption above these doses)^§^

7. Use of oral or transdermal strong opioids (morphine, oxycodone, fentanyl, buprenorphine, diamorphine, methadone, tramadol, pethidine, pentazocine) as first line therapy for mild pain (WHO analgesic ladder not observed)^‡^

8. Digoxin at a long-term dose greater than 125µg/day if eGFR< 30 ml/min/1.73m2 (risk of digoxin toxicity if plasma levels not measured)^¥^

***Four START criteria***

1.Aspirin (75 mg – 160 mg once daily) in the presence of chronic atrial fibrillation, where Vitamin K antagonists or direct thrombin inhibitors or factor Xa inhibitors are contraindicated.^€^

2.Antihypertensive therapy where systolic blood pressure consistently > 160 mmHg and/or diastolic blood pressure consistently >90 mmHg; if systolic blood pressure > 140 mmHg and /or diastolic blood pressure > 90 mmHg, if diabetic.^β^

3.Topical prostaglandin, prostamide or beta-blocker for primary open-angle glaucoma^‡^

4.Proton Pump Inhibitor with severe gastro-esophageal reflux disease or peptic stricture requiring dilatation^‡^

***Reasons for removal***

* self-evident for all clinicians, removed to avoid lengthening the tool without apparent benefit.

†due to ethical concerns that may be encountered in clinical practice and lack of guideline recommendations

^‡^ the drugs are not commonly used in primary care in local practice

^§^ misuse is uncommon in local practice

^¥^ added criteria is more comprehensive: A2. Digoxin at a dose greater than 0.125 mg / day (toxicity risk)

^€^ Updated in the opposite direction

^β^ Updated information

| **ABBREVIATIONS**  ACEI: Angiotensin converting enzyme inhibitors |
| --- |
| eGFR: Estimated Glomerular Filtration Rate |
| NSAID: Non steroidal anti inflammatory drug |
| SSRIs: Selective serotonin reuptake inhibitors |
|  |
