# Supplementary material for: Turkish inappropriate medication use in the elderly (TIME) criteria to improve prescribing in older adults: TIME-to-STOP/TIME-to-START
Source: Eur Geriatr Med. 2020 Mar 5;11(3):491–8. doi: 10.1007/s41999-020-00297-z (PMC7280176; doi:10.1007/s41999-020-00297-z)
Supplement: Supplementary file 3 — Supplementary file3 (DOCX 40 kb) [file 41999_2020_297_MOESM3_ESM.docx]

**Turkish Inappropriate Medication Use in the Elderly (TIME) criteria to improve prescribing in older adults: TIME to STOP/TIME to START**

**Journal name:** European Geriatric Medicine

**Gulistan Bahat**^1^**, Birkan Ilhan**^1^**,** Tugba Erdogan^1^**, Meltem Halil**^2^**, Sumru Savas**^3^**, Zekeriya Ulger**^4^**, Filiz Akyuz**^5^**, Ahmet Kaya Bilge**^6^**, Sibel Cakir**^7^**, Kutay Demirkan** ^8^**, Mustafa Erelel^9^, Kerim Guler**^10^**, Hasmet Hanagasi**^11^**, Belgin Izgi**^12^**, Ates Kadioglu**^13^**, Ayse Karan**^14^**, Isin Baral Kulaksizoglu**^7^**, Ali Mert**^15^**, Savas Ozturk**^16^**, Ilhan Satman**^17^**, Mehmet Sukru Sever**^18^**, Tufan Tukek**^10^**, Yagiz Uresin**^19^**, Onay Yalcin**^20^**, Nilufer Yesilot**^11^**, Meryem Merve Oren^21^, Mehmet Akif Karan**^1^

^1^*Istanbul University, Istanbul Medical School, Department of Internal Medicine, Division of Geriatrics, Istanbul, Turkey*

^2^ *Hacettepe University Faculty of Medicine, Department of Internal Medicine, Division of Geriatric Medicine, Ankara, Turkey.*

^3^ *Ege University Faculty of Medicine, Department of Internal Medicine, Division of Geriatrics, Izmir, Turkey.*

^4^ *Kirikkale University Medical School, Department of Internal Medicine, Kirikkale, Turkey*

^5^*Istanbul University Istanbul Medical School, Department of Internal Medicine, Division of Gastroenterology, Istanbul, Turkey*

^6^*Istanbul University Istanbul Medical School, Department of Cardiology, Istanbul, Turkey*

^7^*Istanbul University Istanbul Medical School, Department of Psychiatry, Istanbul, Turkey*

*^8^Hacettepe University Faculty of Pharmacy, Department of Clinical Pharmacy, Ankara, Turkey.*

^9^ *Istanbul University Istanbul Medical School, Department of Pulmonary Medicine, Istanbul, Turkey*

^10^*Istanbul University Istanbul Medical School, Department of Internal Medicine, Istanbul, Turkey*

^11^*Istanbul University Istanbul Medical School, Department of Neurology, Istanbul, Turkey*

*^12^Istanbul University Istanbul Medical School, Department of Ophthalmology, Istanbul, Turkey*

^13^*Istanbul University Istanbul Medical School, Department of Urology, Istanbul, Turkey*

^14^*Istanbul University Istanbul Medical School, Department of Physical Therapy and Rehabilitation, Istanbul, Turkey*

^15^*Istanbul Medipol University, Infectious Diseases and Clinical Microbiology, Faculty of Medicine, Istanbul, Turkey*

^16^*Haseki Training and Research Hospital, Department of Nephrology, Istanbul, Turkey*

^17^*Istanbul University Istanbul Medical School, Department of Internal Medicine, Division of Endocrinology, Istanbul, Turkey*

^18^*Istanbul University Istanbul Medical School, Department of Internal Medicine, Division of Nephrology, Istanbul, Turkey*

^19^*Istanbul University Istanbul Medical School, Department of Pharmacology, Istanbul, Turkey*

^20^*Istanbul University Istanbul Medical School, Department of Obstetrics and Gynecology, Istanbul, Turkey*

^21^*Istanbul University Istanbul Medical School, Department of Public Health, Istanbul, Turkey*

**Corresponding author:** Gulistan Bahat (**For Reprint**)

**Address:** Istanbul University, Istanbul Medical School, Department of Internal Medicine, Division of Geriatrics, Capa, 34390, Istanbul, Turkey

**Telephone:** + 90 212 414 20 00-33204

**Fax:** + 90 212 532 42 08

**E-mail address:**gbahatozturk@yahoo.com

**TIME-to-STOP Criteria**

The use of this group of medications in the context of the specific criterion possesses “high” side effect potential due to drug-disease, drug-geriatric syndrome and / or drug-drug interaction in the older adults and is considered as “potentially inappropriate medication use”.  Clinicians should decide on all aspects of the patient, taking into account the potential benefits and harms of the drug in the patient (benefit and harm balance) and the treatment goals determined in accordance with the patient/caregiver's preferences. Clinicians may still find it appropriate to use this group of drugs in some cases. If clinicians would prefer to use, careful use with close clinical follow-up in terms of side effects should be implemented.

**Section A: Cardiovascular System criteria.**

A1. Digoxin as first line treatment for atrial fibrillation.

A2. Digoxin at a dose greater than 0.125 mg/day (toxicity risk).

#### A3.Digoxin for heart failure with preserved EF.

#### A4.Diltiazem or verapamil in heart failure with reduced EF.

#### A5.Rate-limiting therapy (beta-blocker, verapamil, diltiazem or digoxin) in patients with bradycardia (<50/ min), type 2 heart block, or complete heart block.

#### A6.Loop diuretic for dependent ankle edema without clinical, biochemical evidence or radiological evidence of heart failure, liver failure, nephrotic syndrome or renal failure (leg elevation and /or compression hosiery usually more appropriate).

#### A7.Use of beta-blockers as first line treatment for essential hypertension in lack of specific beta-blocker indication (increased risk of heart block, fatigue, sexual dysfunction and low activity in stroke protection, additionally, β-adrenergic receptor function decreases with aging).

#### A8.Diuretics as first-line treatment of essential hypertension with concurrent urinary incontinence (may exacerbate urgency and incontinence, impair quality of life and increase falls).

#### A9.Alpha-1 blockers or centrally acting antihypertensives (e.g. methyldopa, rilmenidine, reserpine) in treatment of hypertension, unless clear intolerance of/lack of efficacy with other classes of antihypertensives (heart failure, increased cardiovascular events, orthostatic hypotension, falls, syncope and in women worsening of urinary incontinence by alpha-1 blockers; central nervous system side effects, sedation, depression, parkinsonism, orthostatic hypotension and bradycardia side effects by centrally-active antihypertensives).

#### A10.Vasodilator antihypertensives (alpha-1 blockers, calcium channel blockers) and nitrates in patients with orthostatic hypotension (decrease in systolic blood pressure> 20 mmHg or diastolic blood pressure> 10 mmHg) (risk of exacerbation of orthostatic hypotension).

#### A11. Strict blood pressure control (<140/90 mmHg) in patients with orthostatic hypotension/ cognitive impairment (e.g. dementia)/ functional limitation/ low life expectancy (<2 years)/ high risk of falling.

#### A12. Fludrocortisone for the treatment of orthostatic hypotension without the exclusion of secondary factors and use of non-pharmacological approaches.

#### A13. Beta-blocker in combination with verapamil or diltiazem (risk of heart block).

#### A14. Starting RAS blockers (ACEI, ARB, direct renin inhibitor) or potassium-sparing diuretics (spironolactone, eplerenone, amiloride, triamterene) in patients with serum potassium levels above 5.5 mEq/L.

#### A15. Combination of RAS blockers (ACEI, ARB, direct renin inhibitor) and potassium-sparing diuretics (spironolactone, eplerenone, amiloride, triamterene) without monitoring serum potassium level (risk of dangerous hyperkalemia (i.e. K> 6.0 mEq/L– serum K should be monitored regularly, i.e. at least every 6 months).

#### A16. Potassium-sparing drugs (aldosterone antagonists, triamterene, amiloride, ACEI, ARB) in patients with eGFR<30 ml/min/1.73m2 and whose serum potassium level cannot be closely monitored (risk of hyperkalemia).

#### A17. Thiazide diuretic with concurrent significant hypokalemia (i.e. serum K<3.0 mEq/L), hyponatremia (i.e. serum Na < 130 mEq/L), hypercalcemia (i.e. corrected serum Ca> 10.6 mg/dL) or with a history of gout (hypokalemia, hyponatremia, hypercalcemia and gout can be precipitated by thiazide diuretic).

#### A18. NSAIDs in cases with cardiovascular disease- severe hypertension, heart failure or previous MI, stroke (increased cardiovascular event: MI, stroke, heart failure, and death risk).

#### A19. Beta-blockers in diabetes mellitus with frequent hypoglycemic episodes (risk of suppressing hypoglycemic symptoms).

#### A20. Non-selective beta-blocker (whether oral or topical for glaucoma) with a history of asthma requiring treatment (risk of increased bronchospasm).

#### A21. Long-term aspirin at doses greater than 75-150 mg per day for secondary or primary cardiovascular protection (increased risk of bleeding, no evidence for increased efficacy).

#### A22. Aspirin, clopidogrel, dipyridamole and OACs (Vitamin K antagonists, direct thrombin inhibitor or factor Xa inhibitors) with concurrent significant bleeding risk, i.e. uncontrolled severe hypertension, bleeding diathesis, recent non-trivial spontaneous bleeding) (high risk of bleeding).

#### A23.Aspirin plus clopidogrel as secondary stroke prevention, if the specific indications for the combined use of aspirin and clopidogrel are not present.

#### A24. Aspirin/clopidogrel add on therapy in patients using OAC for chronic atrial fibrillation or for other reasons if there is no additional indication for aspirin/clopidogrel use (no added benefit from aspirin).

#### A25. OACs (vitamin K antagonists, direct thrombin inhibitors or factor Xa inhibitors) for first deep venous thrombosis without continuing provoking risk factors (e.g. thrombophilia) for> 6 months (no proven added benefit).

#### A26. OACs (vitamin K antagonists, direct thrombin inhibitors or factor Xa inhibitors) for first pulmonary embolism without continuing provoking risk factors (e.g. thrombophilia) for > 12 months (no proven added benefit).

#### A27. Aspirin or clopidogrel monotherapy in patients with chronic atrial fibrillation who has contraindication for OAC (vitamin K antagonists, direct thrombin inhibitors or factor Xa inhibitors) use.

#### A28. Dabigatran if eGFR<30 ml/min/1.73 m2.

#### A29. Warfarin in non-valvular atrial fibrillation if malnutrition or irregular food intake is present.

#### A30. Narrow therapeutic indexed medications (such as warfarin, digoxin) in patients with known difficulty in managing therapy (e.g. patients with cognitive impairment) and lack of assistance (e.g., caregivers) (risk of life-threatening toxicity).

#### A31.Prasugrel in patients aged 75 years or older or had TIA/ stroke.

#### A32. Ticlopidine as an antiplatelet agent (clopidogrel or prasugrel or ticagrelor have higher efficacy, stronger evidence and fewer side-effects).

#### A33. Short-acting dipyridamole for antiplatelet-antiaggregant effect (orthostatic hypotension side effect and more effective agents).

#### A34. Statins for primary cardiovascular protection in patients with low-life expectancy (<2 years) or advanced dementia.

#### A35. Allopurinol for asymptomatic hyperuricemia (those without gout or nephrolithiasis) (no evidence for benefit, risk of side effects with the use of xanthine oxidase inhibitors) (there is no evidence that treatment reduces cardiovascular risk or gout).

**Section B: Central Nervous System criteria.**

#### B1. Tricyclic antidepressants (high anticholinergic effect, cognitive side effects, cardiac conduction disorder, orthostatic hypotension, urinary retention, worsening of prostatism, worsening of narrow-angle glaucoma).

#### B2. Paroxetine, fluoxetine and fluvoxamine as the first line treatment among SSRIs (due to high anticholinergic effect of paroxetine, long half-life of fluoxetine, frequent drug interaction with fluoxetine and fluvoxamine).

#### B3. SSRIs with current or recent significant hyponatremia i.e. serum Na<130 mEq/L (risk of exacerbating or precipitating hyponatremia).

#### B4. SNRIs in patients with uncontrolled hypertension.

#### B5. Duloxetine if eGFR<30 ml/min/1.73m2 (increased GIS side effect).

#### B6. Pregabalin and gabapentin without dose reduction if eGFR<30 ml/min/1.73m2.

#### B7. High anticholinergic drugs in patients with delirium or dementia (amitriptyline, paroxetine, dicyclomine, l-hyoscyamine, thioridazine, chlorpromazine, clozapine, olanzapine, urinary antimuscarinics,H1 receptor blockers esp. 1st generation H1 receptor blockers (diphenhydramine, cyproheptadine, pheniramine), H2 receptor blockers (risk of cognitive deterioration).

#### B8. Anticholinergic agents for the treatment of Parkinson's disease (increased risk of side effects, safer and more effective drugs available).

#### B9. Anticholinergics/antimuscarinics to treat extrapyramidal side-effects of neuroleptic / antipsychotic medications (risk of anticholinergic toxicity).

#### B10. Neuroleptics/ antipsychotics in patients with behavioral and psychological symptoms of dementia (BPSD) unless symptoms are severe and non-pharmacological treatments have failed (increased risk of stroke, heart failure, pneumonia-infection, risk of death).

#### B11. Neuroleptics/antipsychotics for hypnotic purpose (increased confusion, hypotension, extrapyramidal side effects, risk of fall).

#### B12. Neuroleptics/ antipsychotics (i.e. other than quetiapine or clozapine) in those with parkinsonism or Lewy Body Disease (risk of severe extrapyramidal symptoms).

#### B13. Neuroleptics/antipsyhotics (may cause gait dyspraxia, parkinsonism), benzodiazepines (sedative, may cause reduced sensorium, impair balance) and Z-type hypnotic (e.g. zopiclone, zolpidem, zaleplon) (may cause protracted daytime sedation, ataxia) in patients with high fall risk.

#### B14. Benzodiazepines for ≥ 4 weeks (risk of prolonged sedation, confusion, impaired balance, falls, road traffic accidents).

#### B15. Benzodiazepines with acute or chronic respiratory failure i.e. PO2 <60 mmHg and / or PCO2> 50 mmHg (risk of exacerbation of respiratory failure).

#### B16. Acetylcholinesterase inhibitors (ChEIs) with a history of persistent bradycardia (<50/min), 2nd or 3rd degree heart block, recurrent unexplained syncope, prolonged QTc (> 470 ms in woman> 450 ms in men) (increased risk for heart conduction defect, syncope, injury risk).

#### B17. Levodopa or dopamine agonists for essential tremor (no evidence of efficacy).

#### B18. Continuous and long-term use of betahistine, trimetazidine, dimenhydrinate in the treatment of vertigo (no evidence-based beneficial effect).

#### B19. Cinnarizine use (extrapyramidal side effects, limited use).

#### B20. Piracetam except for myoclonic convulsion therapy (with no proven clinical efficacy, cost burden and side effect potential).

#### B21. Carbamazepine, phenytoin, phenobarbital or valproate for chronic treatment of epilepsy as first step therapy (negative effects on vitamin D, enzyme induction, risk of falls, also safer alternatives available).

#### B22.Tramadol, neuroleptics/antipsychotics (clozapine, olanzapine, chlorpromazine, thioridazine), bupropion or maprotiline in epilepsy patients.

#### B23. Antiepileptic treatment for seizure prophylaxis due to the presence of ischemic / hemorrhagic stroke in a patient without prior seizure.

#### B24. Citalopram >20 mg / day and escitalopram >10 mg / day  (risk of QTc elongation).

**Section C: Gastrointestinal System criteria.**

#### C1. NSAIDs and OACs (vitamin K antagonist, direct thrombin inhibitor, factor Xa inhibitors) in combination (risk of gastrointestinal bleeding).

#### C2. Aspirin, clopidogrel, NSAIDs or corticosteroids in patients with peptic ulcer history/dyspepsia-gastroesophageal reflux symptomsor with concurrent antiplatelet/anticoagulant/corticosteroid treatment(s) without PPI prophylaxis.

#### C3. Initiation of chronic aspirin or NSAID use without testing for H. pylori in patients with a history of peptic ulcer (complicated or uncomplicated, gastric or duodenal).

#### C4. PPIs for uncomplicated peptic ulcer disease or erosive peptic esophagitis at full therapeutic dose for> 8-12 weeks (dose reduction or earlier discontinuation indicated).

#### C5. PPIs for multiple drug use indication (no benefit, potential harm).

#### C6. Anticholinergic GIS antispasmodics (e.g. hyoscyamine) [increased anticholinergic side effect in older adults (dizziness, decreased cognitive function, blurred vision, arrhythmia, flatulence-constipation) and limited benefit].

#### C7. Drugs likely to cause constipation (e.g. antimuscarinic/anticholinergic drugs, oral iron, opioids, verapamil, aluminum antacids) in patients with chronic constipation where non-constipating alternatives are available (risk of exacerbation of constipation).

#### C8. Metoclopramide or trimethobenzamide as the first line antiemetic treatment of older adults (due to the extrapyramidal side effects and restlessness).

#### C9. Magnesium preparations as laxative or antacid if eGFR<30 ml/min/1.73m2 (risk of hypermagnesemia).

**Section D: Respiratory System criteria.**

#### D1. Antimuscarinic bronchodilators (e.g. ipratropium, tiotropium) with a history of narrow angle glaucoma (may exacerbate glaucoma) or bladder outflow obstruction (may cause urinary retention).

#### D2. Theophylline in the maintenance treatment of COPD or asthma bronchiale (narrow therapeutic index, high insomnia and arrhythmia risk).

#### D3. Systemic corticosteroids instead of inhaled corticosteroids for maintenance therapy in moderate-severe COPD (unnecessary exposure to long-term side-effects of systemic corticosteroids and effective inhaled therapies are available).

**Section E: Musculoskeletal System criteria and analgesic drugs.**

#### E1. Long-term use of NSAIDs (>3 months) in the presence of alternative treatment.

#### E2. NSAIDs if eGFR< 50 ml/min/1.73m2 (risk of deterioration in renal function).

#### E3. Systemic corticosteroids for osteoarthritis (risk of systemic corticosteroid side-effects).

#### E4. Long-term corticosteroids (>3 months) as monotherapy for rheumatoid arthritis (risk of systemic corticosteroid side-effects).

#### E5. Long-term NSAID or colchicine for chronic treatment of gout where there is no contraindication to a xanthine-oxidase inhibitor (e.g. allopurinol, febuxostat) (xanthine-oxidase inhibitors are first choice prophylactic drugs in gout).

#### E6. Colchicine if eGFR <10 ml/min/1.73m2 (risk of colchicine toxicity).

#### E7. Methotrexate if eGFR <30 ml/min/1.73m2.

#### E8. Meperidine in the treatment of pain (increased neurotoxicity, delirium risk, safer alternatives are available. Increased risk in especially patients with renal failure).

#### E9. Extended-release tramadol if eGFR <30 ml/min/1.73m2.

#### E10. Regular opioids without concomitant laxative (risk of severe constipation).

#### E11. Systemic skeletal muscle relaxant agents (thiocolchicoside, tizanidine, chlorzoxazone, carisoprodol, chlorfenese carbamate, cyclobenzaprine, metaxalone, methocarbamol and orphenadrine) for musculoskeletal pain (sedation, dizziness, dry mouth, constipation, cognitive impairment).

#### E12. Initiation of osteoporosis treatment without excluding osteomalacia diagnosis.

#### E13. Conventional vitamin D at high intermittent doses (300,000 IU) as ‘maintenance’ vitamin D therapy (increased fall risk, no additional benefit on the musculoskeletal system).

#### E14. Active vitamin D (1-25 (OH)2 cholecalciferol) (calcitriol) or conventional  vitamin D (25 (OH) cholecalciferol)  in those withhyperphosphatemia and/ or hypercalcemia.

#### E15. Oral bisphosphonates in patients with a history ofupper gastrointestinal disease (i.e. dysphagia, esophagitis, peptic ulcer disease, upper gastrointestinal bleeding or gastroesophageal reflux that cannot be controlled by treatment) and/or those who cannot stand or sit in up right position (risk of relapse/exacerbation of esophagitis, esophageal ulcer, esophageal stricture).

#### E16. Bisphosphonates if eGFR<30 ml/min/1.73m2 (increased risk of acute renal failure).

#### E17. Zoledronate, denosumab or teriparatide without monitoring serum calcium level and assuring adequate calcium/ vitamin D intake prior to the treatment.

**Section F: Urogenital System criteria.**

#### F1. Bladder anticholinergic drugs if PVR>150 mlin men with symptoms of LUTS due to benign prostatic hyperplasia.

#### F2. Bladder anticholinergics in patients with chronic narrow-angle glaucoma.

#### F3. Bladder anticholinergics without PVR determination with concurrent prostatic hyperplasia (risk of obstruction) or diabetes mellitus with complications (risk for neurogenic bladder) or frailty (risk for reduced contractility with detrusor hyperactivity). [Risk of urinary retention and post-renal renal failure].

#### F4. Phosphodiesterase type-5 inhibitors (e.g. sildenafil, tadalafil, vardenafil) in patients with blood pressure <90/50 mmHg or >170/100 mmHg/unstable angina/angina during sexual intercourse/NYHA class IV heart failure/receiving nitrate for angina/ receiving alpha-1 blocker/ recent myocardial infarction (< three months)/ recent stroke (< six months).

#### F5. Non-uroselective alpha-1 blockers (e.g. doxazosin, terazosin) for the treatment of LUTS symptoms related to benign prostatic hyperplasia in patients with orthostatic hypotension.

#### F6. Antibiotic use in asymptomatic bacteriuria except during urological interventions that may damage the mucosa.

#### F7. Nitrofurantoin if eGFR <30 ml/min/1.73m2.

**Section J: Endocrine System criteria.**

#### G1. Intensive glycemic control (HbA1C< 7%) in patients with limited life expectancy (<5 years) or history of falls or cognitive impairment.

#### G2. Metformin in malnourished/frail patients (due to GIS side effects and loss of appetite).

#### G3.Metformin if eGFR <30 ml/min/ 1.73 m2 (risk of lactic acidosis).

#### G4. Sulphonylureas with long duration of action (i.e. glibenclamide or chlorpropamide) in patients with type 2 diabetes mellitus (risk of prolonged hypoglycemia).

#### G5. Thiazolidinediones (i.e. rosiglitazone, pioglitazone) in patients with heart failure/ a history of fracture/ increased fracture risk/ a history of bladder cancer or on insulin treatment (risk of exacerbation of heart failure, increase in the risk of fracture and bladder cancer).

#### G6. Saxagliptin in patients with heart failure.

#### G7. Canagliflozin in patients with fracture/ recurrent urinary tract infection/ genitourinary infection/ severe peripheric artery disease/ lower extremity amputation due to diabetes.

#### G8. SGLT-2 inhibitors for glycemic regulation if eGFR<45 mL/min/1.73 m.

#### G9. Androgens (male sex hormones) in the presence of low serum testosterone levels with no signs and symptoms associated with androgen deficiency.

#### G10.  Systemic estrogens with a history of breast cancer or venous thromboembolism (increased risk of recurrence).

#### G11. Oral estrogens without progestogen in patients with intact uterus (risk of endometrial cancer).

#### G12. Megestrol as an appetite enhancer (minimal effect on weight, prothrombotic side effect).

#### G13. Thyroid hormone in patients with subclinical hypothyroidism (TSH: 4-10 mIU/L; free T4: normal) (no additional benefit, risk of potential side effects such as atrial fibrillation, osteoporosis)

**Section H: Antimuscarinic/anticholinergic drug burden.**

#### H1. High potency anticholinergic drugs [e.g. tricyclic antidepressants, chlorpromazine, thioridazine, clozapine, olanzapine, hyoscine, oral oxybutynin, first generation antihistamines (pheniramine, chlorpheniramine, hydroxyzine, cyproheptadine, dimenhydrinate, diphenhydramine, meclizine etc.), paroxetine] in patients with falls/ constipation/ narrow angle glaucoma/ delirium/ dementia/ urinary retention/ obstructive LUTS symptoms/ concurrent use of anticholinergic drugs.

**Section J: Supplements criteria.**

#### J1. Gingko biloba extract in patients with increased bleeding risk (use of anticoagulants, NSAIDs, history of significant bleeding).

#### J2. Systemic use of Hypericum perforatum (St. John’s Wort) in combination with antidepressants (the risk of serotonergic syndrome especially with SSRI) and drugs metabolized with cytochrome p450 (e.g. digoxin, theophylline, warfarin, carbamazepine, phenytoin, phenobarbital) (Hypericum perforatum (St. John’s Wort) activates cytochrome p450).

#### J3. Supplements with concurrent warfarin (high risk of interaction, increased risk of bleeding).

| **ABBREVIATIONS**  ACEI: Angiotensin converting enzyme inhibitors |
| --- |
| ARB: Angiotensin receptor blockers |
| BPSD: Behavioral and psychological symptoms of dementia |
| ChEIs: Acetylcholinesterase inhibitors |
| COPD: Chronic obstructive pulmonary disease |
| EF: Ejection fraction |
| eGFR: Estimated Glomerular Filtration Rate |
| GIS: Gastrointestinal system |
| H1 receptor: Histamine 1 receptor |
| LUTS: Lower urinary tract symptoms |
| MI: myocardial infarction |
| NSAID: Non steroidal anti inflammatory drug |
| NYHA: New York Heart Association |
| OAC: Oral anticoagulant |
| pO2: Partial pressure of oxygen |
| PPI: Proton pump inhibitors |
| PVR: Post void residual |
| QTc: Corrected QT Interval |
| RAS: Renin angiotensin system |
| SGLT-2: Sodium-glucose cotransporter-2 |
| SNRIs: Serotonin-norepinephrine reuptake inhibitors |
| SSRIs: Selective serotonin reuptake inhibitors |
| TIA: Transient ischemic attack |
| TSH: Thyroid stimulating hormone |
