# Supplementary material for: Turkish inappropriate medication use in the elderly (TIME) criteria to improve prescribing in older adults: TIME-to-STOP/TIME-to-START
Source: Eur Geriatr Med. 2020 Mar 5;11(3):491–8. doi: 10.1007/s41999-020-00297-z (PMC7280176; doi:10.1007/s41999-020-00297-z)
Supplement: Supplementary file 4 — Supplementary file4 (DOCX 32 kb) [file 41999_2020_297_MOESM4_ESM.docx]

**Turkish Inappropriate Medication Use in the Elderly (TIME) criteria to improve prescribing in older adults: TIME to STOP/TIME to START**

**Journal name:** European Geriatric Medicine

**Gulistan Bahat**^1^**, Birkan Ilhan**^1^**,** Tugba Erdogan^1^**, Meltem Halil**^2^**, Sumru Savas**^3^**, Zekeriya Ulger**^4^**, Filiz Akyuz**^5^**, Ahmet Kaya Bilge**^6^**, Sibel Cakir**^7^**, Kutay Demirkan** ^8^**, Mustafa Erelel^9^, Kerim Guler**^10^**, Hasmet Hanagasi**^11^**, Belgin Izgi**^12^**, Ates Kadioglu**^13^**, Ayse Karan**^14^**, Isin Baral Kulaksizoglu**^7^**, Ali Mert**^15^**, Savas Ozturk**^16^**, Ilhan Satman**^17^**, Mehmet Sukru Sever**^18^**, Tufan Tukek**^10^**, Yagiz Uresin**^19^**, Onay Yalcin**^20^**, Nilufer Yesilot**^11^**, Meryem Merve Oren^21^, Mehmet Akif Karan**^1^

^1^*Istanbul University, Istanbul Medical School, Department of Internal Medicine, Division of Geriatrics, Istanbul, Turkey*

^2^ *Hacettepe University Faculty of Medicine, Department of Internal Medicine, Division of Geriatric Medicine, Ankara, Turkey.*

^3^ *Ege University Faculty of Medicine, Department of Internal Medicine, Division of Geriatrics, Izmir, Turkey.*

^4^ *Kirikkale University Medical School, Department of Internal Medicine, Kirikkale, Turkey*

^5^*Istanbul University Istanbul Medical School, Department of Internal Medicine, Division of Gastroenterology, Istanbul, Turkey*

^6^*Istanbul University Istanbul Medical School, Department of Cardiology, Istanbul, Turkey*

^7^*Istanbul University Istanbul Medical School, Department of Psychiatry, Istanbul, Turkey*

*^8^Hacettepe University Faculty of Pharmacy, Department of Clinical Pharmacy, Ankara, Turkey.*

^9^ *Istanbul University Istanbul Medical School, Department of Pulmonary Medicine, Istanbul, Turkey*

^10^*Istanbul University Istanbul Medical School, Department of Internal Medicine, Istanbul, Turkey*

^11^*Istanbul University Istanbul Medical School, Department of Neurology, Istanbul, Turkey*

*^12^Istanbul University Istanbul Medical School, Department of Ophthalmology, Istanbul, Turkey*

^13^*Istanbul University Istanbul Medical School, Department of Urology, Istanbul, Turkey*

^14^*Istanbul University Istanbul Medical School, Department of Physical Therapy and Rehabilitation, Istanbul, Turkey*

^15^*Istanbul Medipol University, Infectious Diseases and Clinical Microbiology, Faculty of Medicine, Istanbul, Turkey*

^16^*Haseki Training and Research Hospital, Department of Nephrology, Istanbul, Turkey*

^17^*Istanbul University Istanbul Medical School, Department of Internal Medicine, Division of Endocrinology, Istanbul, Turkey*

^18^*Istanbul University Istanbul Medical School, Department of Internal Medicine, Division of Nephrology, Istanbul, Turkey*

^19^*Istanbul University Istanbul Medical School, Department of Pharmacology, Istanbul, Turkey*

^20^*Istanbul University Istanbul Medical School, Department of Obstetrics and Gynecology, Istanbul, Turkey*

^21^*Istanbul University Istanbul Medical School, Department of Public Health, Istanbul, Turkey*

**Corresponding author:** Gulistan Bahat (**For Reprint**)

**Address:** Istanbul University, Istanbul Medical School, Department of Internal Medicine, Division of Geriatrics, Capa, 34390, Istanbul, Turkey

**Telephone:** + 90 212 414 20 00-33204

**Fax:** + 90 212 532 42 08

**E-mail address:**gbahatozturk@yahoo.com

**TIME-to-START Criteria**

The use of this group of medications in the context of the specific criterion posseses  indications and potential benefit in older adults, but can often be overlooked in clinical practice, or not prescribed due to advanced age, with no additional valid reason. Not using these drugs in the context of the criterion is considered as “potential inappropriate drug use’’. 

Clinicians should decide on all aspects of the patient, taking into account the potential benefits and harms of the drug in patient (benefit and harm balance) and the treatment goals determined in accordance with the expected life expectancy and patient / caregiver preferences. Clinicians may still find it appropriate not to use these drugs in their patients.

**Section A: Cardiovascular System criteria.**

A1. Antiplatelet therapy (aspirin or clopidogrel) for secondary prevention in patients with documented atherosclerotic coronary artery disease (previous acute coronary syndrome/ coronary artery angioplasty or stenting/ coronary artery bypass grafting/ abdominal aortic aneurysm), documented atherosclerotic cerebrovascular disease (presence of ischemic stroke/TIA/ previous carotid endarterectomy or stenting) or symptomatic lower extremity artery disease.

A2. Statin therapy for secondary prevention in patients with documented atherosclerotic coronary artery disease (previous acute coronary syndrome/ coronary artery angioplasty or stenting/ coronary artery bypass grafting/ abdominal aortic aneurysm), documented atherosclerotic cerebrovascular disease (presence of ischemic stroke/ TIA/ previous carotid endarterectomy or stenting) or peripheral arterial disease*.*

A3. Antihypertensive therapy where systolic blood pressure consistently >160 mmHg and/or diastolic blood pressure consistently >90 mmHg.

A4. OACs (vitamin K antagonists, direct thrombin inhibitors or factor Xa inhibitors) in the presence of chronic non-valvular atrial fibrillation, taking the CHA2DS2-VASc score into account*.*

A5. ACEI with systolic heart failure (EF<= 40%) or ST-elevation myocardial infarction.

A6. Beta-blocker with ischemic heart disease (antianginal effect in chronic ischemic heart disease/ mortality reduction effect in post-MI era) or systolic heart failure (EF<=%40) (bisoprolol/prolonged release metoprolol succinate/carvedilol/nebivolol in systolic heart failure; any beta blocker in ischemic heart disease)*.*

**Section B: Central Nervous System criteria.**

#### B1. Antidepressant treatment in the presence of major depressive disorder.

#### B2. SSRI (or SNRI or pregabalin if SSRI contraindicated) for persistent severe anxiety that interferes with functioning.

#### B3. Acetylcholinesterase inhibitors for mild-moderate Alzheimer’s disease.

#### B4. Memantine for moderate-severe Alzheimer’s disease.

#### B5. Propranolol or primidone for essential tremor that interferes with functioning.

#### B6. L-dopa in idiopathic Parkinson’s disease with functional impairment and disability.

#### B7. Addition of a MAO-B inhibitor or COMT inhibitor to L-dopa treatment when on-off motor fluctuations start in idiopathic Parkinson's disease.

#### B8. Dopamine agonists (ropinirole/pramipexole/rotigotine) or alpha-2-delta calcium channel blockers (pregabalin, gabapentin) for restless legs syndrome if the symptoms affect quality of life adversely and if iron deficiency and severe renal failure have been excluded.

####

**Section C: Gastrointestinal System criteria.**

#### C1. Fiber supplement (psyllium, methylcellulose, polycarbophil, wheat dextrin) or polyethylene glycol for symptomatic constipation without response to lifestyle changes (diet-exercise) after excluding fecal impaction.

**Section D: Respiratory System criteria.**

#### D1. Regular inhaled beta2 agonist or antimuscarinic bronchodilator (e.g. ipratropium, tiotropium) for mild to moderate asthma or COPD.

#### D2. Regular inhaled corticosteroid for moderate-severe asthma or COPD, where FEV1<50% of predicted value and repeated exacerbations requiring treatment with oral corticosteroids.

#### D3. Home continuous oxygen with documented chronic hypoxemia (i.e. pO2 <=55 mmHg or SaO2 <=88%)

**Section E: Musculoskeletal System criteriaand Analgesic drugs.**

#### E1. Vitamin D if vitamin D intake <800-1000 IU per day and/or calcium if elementary calcium intake <1000-1200 mg per day.

#### E2. Bone anti-resorptive (bisphosphonate, denosumab) or anabolic therapy (parathormone analog) in patients with documented osteoporosis [fragility fracture and/or bone mineral density T-scores (femur total, femoral neck or total lumbar)<-2.5].

#### E3. Bisphosphonates in patients started long-term systemic corticosteroid therapy (an anticipated duration of ≥3 months): i) if >= 7.5 mg/day prednisolone or equivalent dose is given,  ii) at any dose if T score is <-1.

#### E4. Long-acting anti-resorptive treatment after discontinuation of at least two doses of denosumab (rebound increased BTMs, BMD loss and increased risk of vertebral fracture following denosumab discontinuation).

#### E5. Antiresorptive treatment after teriparatide treatment.

#### E6. Disease-modifying anti-rheumatic drug with active, chronic rheumatoid disease.

#### E7. Folic acid supplement in patients on methotrexate.

#### E8. Xanthine-oxidase inhibitors (primarily allopurinol) with a history of recurrent episodes of gout.

#### E9. High-potency opioids in moderate-severe pain, if paracetamol, NSAIDs or low-potency opioids are not adequate to the pain severity or have been ineffective.

#### E10. Short-acting opioids in the presence of breakthrough pain (severe pain at intervals) for the patients with chronic pain already on long-acting opioids (risk of uncontrollable severe pain).

**Section F: Endocrine System criteria.**

#### F1. ACEI or ARB in diabetes mellitus with proteinuria (>300 mg/day) or microalbuminuria (>30 mg/day).

**Section G: Urogenital System criteria.**

#### G1. Alpha-1 receptor blocker with moderate-severe (IPSS score) LUTS, where prostatectomy is not considered necessary.

#### G2. 5-alpha reductase inhibitor in addition to alpha-1 receptor blocker with moderate-severe (IPSS score) symptomatic LUTS, if the prostate volume is >30-40 ml and prostatectomy is not considered necessary.

#### G3. Topical vaginal estrogen for symptomatic atrophic vaginitis after failure of non-hormonal treatments.

**Section H: Vaccines criteria.**

#### H1. Seasonal influenza vaccination annually.

#### H2. Pneumococcal vaccination (each one dose for 13-valent conjugate and 23-valent polysaccharide) after age 65.

#### i)in individuals who have not been previously vaccinated, 13-valent conjugate vaccine should be administered as the first dose. One year after conjugate vaccine, 23-valent polysaccharide vaccine should be applied

#### ii) in individuals who have been vaccinated by 23-valent polysaccharide vaccine previously, 13-valent conjugate vaccine should be administered one year later

#### H3.Vaccination for herpes zoster (reduction in risk of shingles infection and post-herpetic neuralgia).

#### H4. Vaccination with Td (tetanus-diphtheria toxoid) every 10 years.

#### H5. Vaccination with meningococcal vaccine for patients who will pilgrimage to Mecca.

**Section I: Supplements criteria.**

#### I1. ONS with MN or MNR if nutritional counseling/dietary supplementation are not sufficient to achieve nutritional goals.

#### I2. ONS for hospitalized older adults with MN or MNR (increases nutrient intake and body weight, reduces the risk of complications and readmissions).

#### I3. ONS for older adults with hip fractures in the postoperative period (regardless of nutritional status) (improves food intake and reduces the risk of complications).

#### I4. ONS with pressure ulcers to ensure adequate protein and energy intake targeting 1.2-2 g/kg/day protein, 30-35 kcal/kg/day energy.

| **ABBREVIATIONS**  ACEI: Angiotensin converting enzyme inhibitors |
| --- |
| ARB: Angiotensin receptor blockers |
| BMD: Bone mineral density |
| BTMs: Bone Turnover Markers |
| COMT: Catechol-O-methyltransferase |
| COPD: Chronic obstructive pulmonary disease |
| EF: Ejection fraction |
| FEV1: Forced expiratory volume in 1 second |
| IPSS: International Prostate Symptom Score |
| LUTS: Lower urinary tract symptoms |
| MAO-B: Monoamine oxidase-B |
| MI: myocardial infarction |
| MN: Malnutrition |
| MNR: Malnutrition risk |
| NSAID: Non steroidal anti inflammatory drug |
| OAC: Oral anticoagulant |
| ONS: Oral nutritional supplements |
| pO2: Partial pressure of oxygen |
| SaO2: Oxygen saturation |
| SNRIs: Serotonin-norepinephrine reuptake inhibitors |
| SSRIs: Selective serotonin reuptake inhibitors |
| TIA: Transient ischemic attack |
