# Supplementary material for: Turkish inappropriate medication use in the elderly (TIME) criteria to improve prescribing in older adults: TIME-to-STOP/TIME-to-START
Source: Eur Geriatr Med. 2020 Mar 5;11(3):491–8. doi: 10.1007/s41999-020-00297-z (PMC7280176; doi:10.1007/s41999-020-00297-z)
Supplement: Supplementary file 5 — Supplementary file5 (DOCX 146 kb) [file 41999_2020_297_MOESM5_ESM.docx]

**Turkish Inappropriate Medication Use in the Elderly (TIME) criteria to improve prescribing in older adults: TIME to STOP/TIME to START**

**Journal name:** European Geriatric Medicine

**Gulistan Bahat**^1^**, Birkan Ilhan**^1^**,** Tugba Erdogan^1^**, Meltem Halil**^2^**, Sumru Savas**^3^**, Zekeriya Ulger**^4^**, Filiz Akyuz**^5^**, Ahmet Kaya Bilge**^6^**, Sibel Cakir**^7^**, Kutay Demirkan** ^8^**, Mustafa Erelel^9^, Kerim Guler**^10^**, Hasmet Hanagasi**^11^**, Belgin Izgi**^12^**, Ates Kadioglu**^13^**, Ayse Karan**^14^**, Isin Baral Kulaksizoglu**^7^**, Ali Mert**^15^**, Savas Ozturk**^16^**, Ilhan Satman**^17^**, Mehmet Sukru Sever**^18^**, Tufan Tukek**^10^**, Yagiz Uresin**^19^**, Onay Yalcin**^20^**, Nilufer Yesilot**^11^**, Meryem Merve Oren^21^, Mehmet Akif Karan**^1^

^1^*Istanbul University, Istanbul Medical School, Department of Internal Medicine, Division of Geriatrics, Istanbul, Turkey*

^2^ *Hacettepe University Faculty of Medicine, Department of Internal Medicine, Division of Geriatric Medicine, Ankara, Turkey.*

^3^ *Ege University Faculty of Medicine, Department of Internal Medicine, Division of Geriatrics, Izmir, Turkey.*

^4^ *Kirikkale University Medical School, Department of Internal Medicine, Kirikkale, Turkey*

^5^*Istanbul University Istanbul Medical School, Department of Internal Medicine, Division of Gastroenterology, Istanbul, Turkey*

^6^*Istanbul University Istanbul Medical School, Department of Cardiology, Istanbul, Turkey*

^7^*Istanbul University Istanbul Medical School, Department of Psychiatry, Istanbul, Turkey*

*^8^Hacettepe University Faculty of Pharmacy, Department of Clinical Pharmacy, Ankara, Turkey.*

^9^ *Istanbul University Istanbul Medical School, Department of Pulmonary Medicine, Istanbul, Turkey*

^10^*Istanbul University Istanbul Medical School, Department of Internal Medicine, Istanbul, Turkey*

^11^*Istanbul University Istanbul Medical School, Department of Neurology, Istanbul, Turkey*

*^12^Istanbul University Istanbul Medical School, Department of Ophthalmology, Istanbul, Turkey*

^13^*Istanbul University Istanbul Medical School, Department of Urology, Istanbul, Turkey*

^14^*Istanbul University Istanbul Medical School, Department of Physical Therapy and Rehabilitation, Istanbul, Turkey*

^15^*Istanbul Medipol University, Infectious Diseases and Clinical Microbiology, Faculty of Medicine, Istanbul, Turkey*

^16^*Haseki Training and Research Hospital, Department of Nephrology, Istanbul, Turkey*

^17^*Istanbul University Istanbul Medical School, Department of Internal Medicine, Division of Endocrinology, Istanbul, Turkey*

^18^*Istanbul University Istanbul Medical School, Department of Internal Medicine, Division of Nephrology, Istanbul, Turkey*

^19^*Istanbul University Istanbul Medical School, Department of Pharmacology, Istanbul, Turkey*

^20^*Istanbul University Istanbul Medical School, Department of Obstetrics and Gynecology, Istanbul, Turkey*

^21^*Istanbul University Istanbul Medical School, Department of Public Health, Istanbul, Turkey*

**Corresponding author:** Gulistan Bahat (**For Reprint**)

**Address:** Istanbul University, Istanbul Medical School, Department of Internal Medicine, Division of Geriatrics, Capa, 34390, Istanbul, Turkey

**Telephone:** + 90 212 414 20 00-33204

**Fax:** + 90 212 532 42 08

**E-mail address:**gbahatozturk@yahoo.com

**TIME-to-STOP Criteria with References and Explanations**

The use of this group of medications in the context of the specific criterion possesses “high” side effect potential due to drug-disease, drug-geriatric syndrome and / or drug-drug interaction in the older adults and is considered as “potentially inappropriate medication use”.  Clinicians should decide on all aspects of the patient, taking into account the potential benefits and harms of the drug in the patient (benefit and harm balance) and the treatment goals determined in accordance with the patient/caregiver's preferences. Clinicians may still find it appropriate to use this group of drugs in some cases. If clinicians would prefer to use, careful use with close clinical follow-up in terms of side effects should be implemented.

#### Explanations that are added to some criteria to aid in clinical use are given in italics right after the criterion in italics with the prefix *.

#### The references include references of the criteria and the explanations if present.

**Section A: Cardiovascular System criteria.**

**A1. Digoxin as first line treatment for atrial fibrillation**.
**Digoxin can be used in atrial fibrillation cases where beta-blocker and calcium channel blockers cannot be tolerated (e.g. due to hypotension) or as combination therapy in cases where these treatments are insufficient.*

A1(i): By the 2019 American Geriatrics Society Beers Criteria® Update Expert Panel. American Geriatrics Society 2019 Updated AGS Beers Criteria® for Potentially Inappropriate Medication Use in Older Adults. J Am Geriatr Soc. 2019Apr;67(4):674-694. doi: 10.1111/jgs.15767. Epub 2019 Jan 29.

#### A1(ii): Kirchhof P, Benussi S, Kotecha D, Ahlsson A, Atar D, Casadei B, Castella M, Diener HC, Heidbuchel H, Hendriks J, Hindricks G, Manolis AS, Oldgren J, Popescu BA, Schotten U, Van Putte B, Vardas P; ESC Scientific Document Group. 2016 ESC Guidelines for the management of atrial fibrillation developed in collaboration with EACTS. Eur Heart J. 2016 Oct 7;37(38):2893-2962. doi:10.1093/eurheartj/ehw210. Epub 2016 Aug 27.

#### A2. Digoxin at a dose greater than 0.125 mg/day (toxicity risk).

A2(i):By the 2019 American Geriatrics Society Beers Criteria® Update Expert Panel. American Geriatrics Society 2019 Updated AGS Beers Criteria® for Potentially Inappropriate Medication Use in Older Adults. J Am Geriatr Soc. 2019Apr;67(4):674-694. doi: 10.1111/jgs.15767. Epub 2019 Jan 29.

A2(ii):Digoxin: Drug information, Lexicomp Online. Last accessed date 22 October 2019.

A2(iii): British National Formulary vol. 76, September 2018-March 2019: p 28.

#### A3.Digoxin for heart failure with preserved EF.  **Digoxin may have an indication for concomitant atrial fibrillation (see A1).*

A3(i): Jessup M, Abraham WT, Casey DE, Feldman AM, Francis GS, Ganiats TG, KonstamMA, Mancini DM, Rahko PS, Silver MA, Stevenson LW, Yancy CW.2009 focused update: ACCF/AHA Guidelines for the Diagnosis and Management of Heart Failure in Adults:a report of the American College of Cardiology Foundation/American Heart Association Task Force on Practice Guidelines: developed in collaboration with the International Society for Heart and Lung Transplantation. Circulation 2009; 119(14): 1977-2016.

A3 (ii): Cheng JW, Nayar M. A review of heart failure management in the elderly population. Am J Geriatr Pharmacother 2009; 7(5): 233-49. Review.

A3(iii): O'Mahony D, O'Sullivan D, Byrne S, O'Connor MN, Ryan C, Gallagher P. STOPP/START criteria for potentially inappropriate prescribing in older people: version 2. Age Ageing. 2015 Mar;44(2):213-8. doi: 10.1093/ageing/afu145. Epub 2014 Oct 16. Review.

#### A4.Diltiazem or verapamil in heart failure with reduced EF.

A4(i): By the 2019 American Geriatrics Society Beers Criteria® Update Expert Panel. American Geriatrics Society 2019 Updated AGS Beers Criteria® for Potentially Inappropriate Medication Use in Older Adults. J Am Geriatr Soc. 2019Apr;67(4):674-694. doi: 10.1111/jgs.15767. Epub 2019 Jan 29.

A4(ii): Ponikowski P, Voors AA, Anker SD, Bueno H, Cleland JGF, Coats AJS, Falk V, González-Juanatey JR, Harjola VP, Jankowska EA, Jessup M, Linde C, Nihoyannopoulos P, Parissis JT, Pieske B, Riley JP, Rosano GMC, Ruilope LM, Ruschitzka F, Rutten FH, van der Meer P; ESC Scientific Document Group. 2016 ESC Guidelines for the diagnosis and treatment of acute and chronic heart failure: The Task Force for the diagnosis and treatment of acute and chronic heart failure of the European Society of Cardiology (ESC)Developed with the special contribution of the Heart Failure Association (HFA) of the ESC. Eur Heart J. 2016 Jul 14;37(27):2129-2200. doi: 10.1093/eurheartj/ehw128. Epub 2016 May 20. Erratum in: Eur Heart J. 2016 Dec 30

A4 (iii): O'Mahony D, O'Sullivan D, Byrne S, O'Connor MN, Ryan C, Gallagher P. STOPP/START criteria for potentially inappropriate prescribing in older people: version 2. Age Ageing. 2015 Mar;44(2):213-8. doi: 10.1093/ageing/afu145. Epub 2014 Oct 16. Review.

#### A5.Rate-limiting therapy (beta-blocker, verapamil, diltiazem or digoxin) in patients with bradycardia (<50/ min), type 2 heart block, or complete heart block.  **Beta-blockers are relatively contraindicated if PR interval>240 msec. In patients who are currently using betablockers, diltiazem, verapamil or digoxin, dose reduction should be performed in case of bradycardia (<50 / min).*

A5(i):British National Formulary, No. 76, September 2018-March 2019, p 145.

A5(ii): O'Mahony D, O'Sullivan D, Byrne S, O'Connor MN, Ryan C, Gallagher P. STOPP/START criteria for potentially inappropriate prescribing in older people: version 2. Age Ageing. 2015 Mar;44(2):213-8. doi: 10.1093/ageing/afu145. Epub 2014 Oct 16. Review.

#### A6.Loop diuretic for dependent ankle edema without clinical, biochemical evidence or radiological evidence of heart failure, liver failure, nephrotic syndrome or renal failure (leg elevation and /or compression hosiery usually more appropriate).

A6(i): Wehling M. Morbus diureticus in the elderly: epidemic overuse of a widely applied group of drugs. J Am Med Dir Assoc 2013; 14(6): 437-42. Review.

A6(ii): Sarafidis PA, Georgianos PI, Lasaridis AN. Diuretics in clinical practice. Part I: mechanisms of action, pharmacological effects and clinical indications of diuretic compounds. Expert Opin Drug Saf 2010; 9(2):243-57. Review.

A6(iii): O'Mahony D, O'Sullivan D, Byrne S, O'Connor MN, Ryan C, Gallagher P. STOPP/START criteria for potentially inappropriate prescribing in older people: version 2. Age Ageing. 2015 Mar;44(2):213-8. doi: 10.1093/ageing/afu145. Epub 2014 Oct 16. Review.

A6(iv): British National Formulary, No. 76, September 2018-March 2019, p28.

#### A7.Use of beta-blockers as first line treatment for essential hypertension in lack of specific beta-blocker indication (increased risk of heart block, fatigue, sexual dysfunction and low activity in stroke protection, additionally, β-adrenergic receptor function decreases with aging).

A7(i): Whelton PK, Carey RM, Aronow WS, Casey DE Jr, Collins KJ, Dennison Himmelfarb C, DePalma SM, Gidding S, Jamerson KA, Jones DW, MacLaughlin EJ, Muntner P, Ovbiagele B, Smith SC Jr, Spencer CC, Stafford RS, Taler SJ, Thomas RJ, Williams KA Sr, Williamson JD, Wright JT Jr. 2017 ACC/AHA/AAPA/ABC/ACPM/AGS/APhA/ASH/ASPC/NMA/ PCNA Guideline for the Prevention, Detection, Evaluation, and Management of High Blood Pressure in Adults: ExecutiveSummary: A Report of the American College of Cardiology/American Heart Association Task Force on Clinical Practice Guidelines. Circulation. 2018 Oct 23;138(17):e426-e483

A7(ii): James PA, Oparil S, Carter BL, Cushman WC, Dennison-Himmelfarb C, Handler J, Lackland DT, LeFevre ML, MacKenzie TD, Ogedegbe O, Smith SC Jr, Svetkey LP, Taler SJ, Townsend RR, Wright JT Jr, Narva AS, Ortiz E. 2014 evidence-based guideline for the management of high blood pressure in adults: report from the panel members appointed to the Eighth Joint National Committee (JNC 8). JAMA. 2014 Feb 5;311(5):507-20.

A7(iii): Alexander K P, Peterson E D, Coronary heart disease in Hazzards Geriatric Medicine and Gerontology Seventh edition . Eds. Haler J B, Ouslander J G, Studenski S, High K P, Asthana S, Ritchie C S, Supiano M A,; 2017

A7(iv): Arıcı M, Birdane A, Güler K, Yıldız BO, Altun B, Ertürk Ş, Aydoğdu S, Özbakkaloğlu M, Ersöz HÖ, Süleymanlar G, Tükek T, Tokgözoğlu L, Erdem Y; Türk Kardiyoloji Derneği (TKD); Türk İç Hastalıkları Uzmanlık Derneği (TİHUD); Türkiye Endokrinoloji ve Metabolizma Derneği (TEMD); Türk Nefroloji Derneği (TND); Türk Hipertansiyon ve Böbrek Hastalıkları Derneği. [Turkish Hypertension Consensus Report]. Turk Kardiyol Dern Ars. 2015 Jun;43(4):402-9. doi: 10.5543/tkda.2015.16243. Turkish.

A7(v): Williams B, Mancia G, Spiering W, Agabiti Rosei E, Azizi M, Burnier M, Clement DL, Coca A, de Simone G, Dominiczak A, Kahan T, Mahfoud F, Redon J, Ruilope L, Zanchetti A, Kerins M, Kjeldsen SE, Kreutz R, Laurent S, Lip GYH, McManus R, Narkiewicz K, Ruschitzka F, Schmieder RE, Shlyakhto E, Tsioufis C, Aboyans V, Desormais I; ESC Scientific Document Group . 2018 ESC/ESH Guidelines for the management of arterial hypertension. Eur Heart J. 2018 Sep 1;39(33):3021-3104.

#### A8.Diuretics as first-line treatment of essential hypertension with concurrent urinary incontinence (may exacerbate urgency and incontinence, impair quality of life and increase falls). **Diuretics can be a risk factor for falls and impair quality of life by causing volume depletion and increasing the frequency and volume of micturition. Patients using diuretics should closely be monitored in these aspects.*

A8(i):Lukacz E. Evaluation of women with urinary incontinence.In: UpToDate, Post, TW (Ed), UpToDate, Waltham, MA, 2019 last accessed date 23 October 2019

A8(ii): Ekundayo OJ. The association between overactive bladder and diuretic use in the elderly. Curr Urol Rep 2009; 10(6):434-40. Review.

A8(iii): Ekundayo OJ, Markland A, Lefante C, Sui X, Goode PS, Allman RM, Ali M, Wahle C, Thornton PL, Ahmed A. Association of diuretic use and overactive bladder syndrome in older adults: a propensity score analysis. Arch Gerontol Geriatr2009; 49(1):64-8.

A8(iv): Finkelstein MM. Medical conditions, medications, and urinary incontinence. Analysis of a population-based survey. Can Fam Physician 2002; 48:96-101.

A8(V): O'Mahony D, O'Sullivan D, Byrne S, O'Connor MN, Ryan C, Gallagher P. STOPP/START criteria for potentially inappropriate prescribing in older people: version 2. Age Ageing. 2015 Mar;44(2):213-8. doi: 10.1093/ageing/afu145. Epub 2014 Oct 16. Review.

#### A9.Alpha-1 blockers or centrally acting antihypertensives (e.g. methyldopa, rilmenidine, reserpine) in treatment of hypertension, unless clear intolerance of/lack of efficacy with other classes of antihypertensives (heart failure, increased cardiovascular events, orthostatic hypotension, falls, syncope and in women worsening of urinary incontinence by alpha-1 blockers; central nervous system side effects, sedation, depression, parkinsonism, orthostatic hypotension and bradycardia side effects by centrally-active antihypertensives).

A9 (i): By the 2019 American Geriatrics Society Beers Criteria® Update Expert Panel. American Geriatrics Society 2019 Updated AGS Beers Criteria® for Potentially Inappropriate Medication Use in Older Adults. J Am Geriatr Soc. 2019Apr;67(4):674-694. doi: 10.1111/jgs.15767. Epub 2019 Jan 29.

A9 (ii): Marshall HJ, Beevers DG. Alpha-adrenoceptor blocking drugs and female urinary incontinence: prevalence and reversibility. Br J Clin Pharmacol. 1996 Oct;42(4):507-9

A9 (iii): Major cardiovascular events in hypertensive patients randomized to doxazosinvs chlorthalidone: the antihypertensive and lipid-lowering treatment to preventheart attack trial (ALLHAT). ALLHAT Collaborative Research Group. JAMA. 2000 Apr19;283(15):1967-75.

A9(iv): O'Mahony D, O'Sullivan D, Byrne S, O'Connor MN, Ryan C, Gallagher P. STOPP/START criteria for potentially inappropriate prescribing in older people: version 2. Age Ageing. 2015 Mar;44(2):213-8. doi: 10.1093/ageing/afu145. Epub 2014 Oct 16. Review.

#### A10.Vasodilator antihypertensives (alpha-1 blockers, calcium channel blockers) and nitrates in patients with orthostatic hypotension (decrease in systolic blood pressure> 20 mmHg or diastolic blood pressure> 10 mmHg) (risk of exacerbation of orthostatic hypotension). **In cases with orthostatic hypotension vasodilator antihypertensives may only be used when severe supine hypertension cannot be controlled with other antihypertensives. If they would be used, preventive measures from orthostatic hypotension must be taken simultaneously. In the presence of orthostatic hypotension, all antihypertensives should be evaluated in terms of dose reduction.*

A10(i): Aronow WS. Treating hypertension in older adults: safety considerations. Drug Saf 2009; 32(2): 111-8.

A10(ii): Verhaeverbeke I, Mets T. Drug-induced orthostatic hypotension in the elderly: avoiding its onset. Drug Saf 1997; 17(2): 105-18. Review.

A10(iii): O'Mahony D, O'Sullivan D, Byrne S, O'Connor MN, Ryan C, Gallagher P. STOPP/START criteria for potentially inappropriate prescribing in older people: version 2. Age Ageing. 2015 Mar;44(2):213-8. doi: 10.1093/ageing/afu145. Epub 2014 Oct 16. Review.

A10(iv): Brignole M, Moya A, de Lange FJ, Deharo JC, Elliott PM, Fanciulli A, Fedorowski A, Furlan R, Kenny RA, Martín A, Probst V, Reed MJ, Rice CP, Sutton R, Ungar A, van Dijk JG; ESC Scientific Document Group . 2018 ESC Guidelines for the diagnosis and management of syncope. Eur Heart J. 2018 Jun 1;39(21):1883-1948.

#### A11. Strict blood pressure control (<140/90 mmHg) in patients with orthostatic hypotension/ cognitive impairment (e.g. dementia)/ functional limitation/ low life expectancy (<2 years)/ high risk of falling.

A11(i):Onder G, Landi F, Fusco D, Corsonello A, Tosato M, Battaglia M, Mastropaolo S, Settanni S, Antocicco M, Lattanzio F. Recommendations to prescribe in complex older adults: results of the CRIteria to assess appropriate Medication use among Elderly complex patients (CRIME) project. Drugs Aging. 2014 Jan;31(1):33-45.Review.

A11(ii): Wu JS, Yang YC, Lu FH. Population-based study on the prevalence and risk factors of orthostatic hypotension in subjects withpre-diabetes and diabetes. Diabetes Care. 2009;32:69–74.

A11(iii): Luukinen H, Koski K, Laippala P, Kivela¨ SL. Prognosis of diastolic and systolic orthostatic hypotension in older persons. ArchIntern Med. 1999;159:273–80.

A(iv): Hiitola P, Enlund H, Kettunen R. Postural changes in blood pressure and the prevalence of orthostatic hypotension among home-dwelling elderly aged 75 years or older. J Hum Hypertens. 915 2009;23:33–9.

#### A12. Fludrocortisone for the treatment of orthostatic hypotension without the exclusion of secondary factors and use of non-pharmacological approaches. **Non-pharmacological approaches for the management of orthostatic hypotension include: standing up slowly, lower extremity resistance exercises, wearing stockings for varices, adequate fluid intake [2-3 L/ day], avoiding alcohol, eating less and frequently, adequate salt intake [6 -10g/ day], avoiding carbohydrate-rich foods, avoiding intense exercise in hot weather, keeping the head 30 to 45 degrees high while lying.*

A12(i):Seppi K, Weintraub D, Coelho M, Perez-Lloret S, Fox SH, Katzenschlager R,Hametner EM, Poewe W, Rascol O, Goetz CG, Sampaio C. The Movement DisorderSociety Evidence-Based Medicine Review Update: Treatments for the non-motorsymptoms of Parkinson's disease. Mov Disord. 2011 Oct;26 Suppl 3:S42-80.

A12(ii): Kaufmann H. Treatment of orthostatic and postprandial hypotension. In: UpToDate, Post, TW (Ed), UpToDate, Waltham, MA, 2019 last accessed date 23 October 2019

A12(iii): Shibao C, Lipsitz LA, Biaggioni I. ASH position paper: evaluation and treatment of orthostatic hypotension. J Clin Hypertens (Greenwich). 2013 Mar;15(3):147-53.

#### A13. Beta-blocker in combination with verapamil or diltiazem (risk of heart block). **Among calcium channel blockers, long-acting dihydropyridine group should be preferred for the treatment of hypertension in older adults (use of verapamil or diltiazem may increase the risk of heart block).*

#### **Combination of beta-blockers with verapamil/ diltiazem may seldomly be appropriate in some cases of supraventricular tachycardia. This should also be used with caution.*

A13(i):Edoute Y, Nagachandran P, Svirski B, Ben-Ami H. Cardiovascular adverse drug reaction associated with combined beta-adrenergic and calcium entry-blocking agents. J Cardiovasc Pharmacol 2000; 35(4): 556-9.

A13(ii): Egan BM. Treatment of hypertension in older adults, particularly isolated systolic hypertension.In: UpToDate, Post, TW (Ed), UpToDate, Waltham, MA, 2019 last accessed date 23 October 2019

A13(iii): O'Mahony D, O'Sullivan D, Byrne S, O'Connor MN, Ryan C, Gallagher P. STOPP/START criteria for potentially inappropriate prescribing in older people: version 2. Age Ageing. 2015 Mar;44(2):213-8. doi: 10.1093/ageing/afu145. Epub 2014 Oct 16. Review.

#### A14. Starting RAS blockers (ACEI, ARB, direct renin inhibitor) or potassium-sparing diuretics (spironolactone, eplerenone, amiloride, triamterene) in patients with serum potassium levels above 5.5 mEq/L.  **In cases with a potassium level above 6 mEq/ L, if being used, they should be discontinued.* **Combined use of ACEI and ARBs for antihypertensive purpose is not appropriate.*

A14(i): Izzo JL Jr, Weir MR. Angiotensin-converting enzyme inhibitors. J Clin Hypertens (Greenwich) 2011; 13(9):667-75. Review.

A14(ii): Desai AS, Swedberg K, McMurray JJ, Granger CB, Yusuf S, Young JB, Dunlap ME, Solomon SD, Hainer JW, Olofsson B, Michelson EL, Pfeffer MA; CHARM Program Investigators. Incidence and predictors of hyperkalemia in patients with heart failure: an analysis of the CHARM Program. J Am Coll Cardiol 2007 Nov 13;50(20):1959-66.

A14(iii): Reardon LC, Macpherson DS. Hyperkalemia in outpatients using angiotensin-converting enzyme inhibitors. How much should we worry? Arch Intern Med 1998; 158(1):26-32.

A14(iv): ONTARGET Investigators, Yusuf S, Teo KK, Pogue J, Dyal L, Copland I, Schumacher H, Dagenais G, Sleight P, Anderson C. Telmisartan, ramipril, or both in patients at high risk for vascular events. N Engl J Med. 2008 Apr 10;358(15):1547-59.

A14(v): O'Mahony D, O'Sullivan D, Byrne S, O'Connor MN, Ryan C, Gallagher P. STOPP/START criteria for potentially inappropriate prescribing in older people: version 2. Age Ageing. 2015 Mar;44(2):213-8. doi: 10.1093/ageing/afu145. Epub 2014 Oct 16. Review.

#### A15. Combination of RAS blockers (ACEI, ARB, direct renin inhibitor) and potassium-sparing diuretics (spironolactone, eplerenone, amiloride, triamterene) without monitoring serum potassium level (risk of dangerous hyperkalemia) (i.e. K> 6.0 mEq/L– serum K should be monitored regularly, i.e. at least every 6 months)  **This risk is particularly high in patients with diabetes mellitus, renal failure, or those using potassium salt supplements.*

A15(i): Bauersachs J, Fraccarollo D. Aldosterone antagonism in addition to angiotensin-converting enzyme inhibitors in heart failure. Minerva Cardioangiol 2003; 51(2):155-64. Review.

A15(ii): Poggio R, Grancelli HO, Miriuka SG. Understanding the risk of hyperkalaemia in heart failure: role of aldosterone antagonism. Postgrad Med J 2010; 86 (1013):136-42. Review.

A15(iii): Wrenger E, Müller R, Moesenthin M, Welte T, Frölich JC, Neumann KH. Interaction of spironolactone with ACE inhibitors or angiotensin receptor blockers: analysis of 44 cases. BMJ 2003; 327(7407):147-9.

A15(iv): Marcy TR, Ripley TL. Aldosterone antagonists in the treatment of heart failure. Am J Health Syst Pharm 2006; 63(1): 49-58.

A15(v): Tang WH, Parameswaran AC, Maroo AP, Francis GS. Aldosterone receptor antagonists in the medical management of chronic heart failure. Mayo Clin Proc 2005; 80(12): 1623-30. Review.

A15(vi): O'Mahony D, O'Sullivan D, Byrne S, O'Connor MN, Ryan C, Gallagher P. STOPP/START criteria for potentially inappropriate prescribing in older people: version 2. Age Ageing. 2015 Mar;44(2):213-8. doi: 10.1093/ageing/afu145. Epub 2014 Oct 16. Review.

#### A16. Potassium-sparing drugs (aldosterone antagonists, triamterene, amiloride, ACEI, ARB) in patients with eGFR<30 ml/min/1.73m2 and whose serum potassium level cannot be closely monitored (risk of hyperkalemia)

A16(i): Kidney Disease Outcomes Quality Initiative (K/DOQI). K/DOQI clinical practice guidelines on hypertension and antihypertensive agents in chronic kidney disease. Am J Kidney Dis. 2004 May;43(5 Suppl 1):S1-290.

#### A17. Thiazide diuretic with concurrent significant hypokalemia (i.e. serum K<3.0 mEq/L), hyponatremia (i.e. serum Na < 130 mEq/L), hypercalcemia (i.e. corrected serum Ca> 10.6 mg/dL) or with a history of gout (hypokalemia, hyponatremia, hypercalcemia and gout can be precipitated by thiazide diuretic).

A17(i):Sica DA, Carter B, Cushman W, Hamm L. Thiazide and loop diuretics. J Clin Hypertens (Greenwich) 2011; 13(9):639-43. Review.

A17(ii): Gurwitz JH, Kalish SC, Bohn RL, Glynn RJ, Monane M, Mogun H, Avorn J. Thiazide diuretics and the initiation of anti-gout therapy. J ClinEpidemiol1997; 50(8): 953-9.

A17(iii): O'Mahony D, O'Sullivan D, Byrne S, O'Connor MN, Ryan C, Gallagher P. STOPP/START criteria for potentially inappropriate prescribing in older people: version 2. Age Ageing. 2015 Mar;44(2):213-8. doi: 10.1093/ageing/afu145. Epub 2014 Oct 16. Review.

#### A18. NSAIDs in cases with cardiovascular disease- severe hypertension, heart failure or previous MI, stroke (increased cardiovascular event: MI, stroke, heart failure, and death risk).  **In cases where NSAID use is clinically indicated, short- term use may be preferred with close clinical follow-up and at the lowest possible dose.* **Although all NSAIDs have cardiovascular risks, naproxen and ibuprofen may be relatively safe.* **If NSAIDs would be administered in the patient who is using aspirin, it may be more appropriate to administer at least 2 hours later after aspirin.*

A18(i): Pilotto A, Sancarlo D, Addante F, Scarcelli C, Franceschi M. Non-steroidal anti-inflammatory drug use in the elderly. Surg Oncol 2010; 19(3): 167-72. Review.

A18 (ii): Strand V. Are COX-2 inhibitors preferable to non-selective non-steroidal anti-inflammatory drugs in patients with risk of cardiovascular events taking low-dose aspirin? Lancet 2007; 370(9605): 2138-51. Review.

A18(iii): White WB. Defining the problem of treating the patient with hypertension and arthritis pain. Am J Med. 2009; 122(5 Suppl): S3-9. Review.

A18(iv): [Park KE.Qin Y](http://0-www.embase.com.library.ucc.ie/search/results), [Bavry AA.](http://0-www.embase.com.library.ucc.ie/search/results)Nonsteroidal anti-inflammatory drugs and their effects in the elderly. Aging Health 2012; 8(2): 167-177.

A18(v):Solomon DH.NSAIDs: Adverse cardiovascular effects. In: UpToDate, Post, TW (Ed), UpToDate, Waltham, MA, 2019 last accessed date 23 October 2019

A18(vi): By the 2019 American Geriatrics Society Beers Criteria® Update Expert Panel. American Geriatrics Society 2019 Updated AGS Beers Criteria® for Potentially Inappropriate Medication Use in Older Adults. J Am Geriatr Soc. 2019Apr;67(4):674-694. doi: 10.1111/jgs.15767. Epub 2019 Jan 29.

A18(vii): O'Mahony D, O'Sullivan D, Byrne S, O'Connor MN, Ryan C, Gallagher P. STOPP/START criteria for potentially inappropriate prescribing in older people: version 2. Age Ageing. 2015 Mar;44(2):213-8. doi: 10.1093/ageing/afu145. Epub 2014 Oct 16. Review.

#### A19. Beta-blockers in diabetes mellitus with frequent hypoglycemic episodes (risk of suppressing hypoglycemic symptoms).

A19(i): Chelliah A, Burge MR. Hypoglycaemia in elderly patients with diabetes mellitus: causes and strategies for prevention. Drugs Aging 2004; 21(8): 511-30. Review.

A19 (ii): British National Formulary vol. 76, September 2018-March 2019: p 145.

A19(iii): O'Mahony D, O'Sullivan D, Byrne S, O'Connor MN, Ryan C, Gallagher P. STOPP/START criteria for potentially inappropriate prescribing in older people: version 2. Age Ageing. 2015 Mar;44(2):213-8. doi: 10.1093/ageing/afu145. Epub 2014 Oct 16. Review.

#### A20. Non-selective beta-blocker (whether oral or topical for glaucoma) with a history of asthma requiring treatment (risk of increased bronchospasm).

A20(i): O'Mahony D, O'Sullivan D, Byrne S, O'Connor MN, Ryan C, Gallagher P. STOPP/START criteria for potentially inappropriate prescribing in older people: version 2. Age Ageing. 2015 Mar;44(2):213-8. doi: 10.1093/ageing/afu145. Epub 2014 Oct 16. Review.

A20(ii): Kaiserman I, Fendyur A, Vinker S. Topical beta blockers in asthmatic patients-is it safe? Curr Eye Res. 2009 Jul;34(7):517-22.

A20(iii): McNeill RS, Ingram CG. Effect of propranolol on ventilatory function. Am J Cardiol. 1966 Sep;18(3):473-5.

A20(iV): Morales DR, Lipworth BJ, Donnan PT, Jackson C, Guthrie B. Respiratory effect of beta-blockers in people with asthma and cardiovascular disease: population-based nested case control study. BMC Med. 2017 Jan 27;15(1):18.

#### A21. Long-term aspirin at doses greater than 75-150 mg per day for secondary or primary cardiovascular protection (increased risk of bleeding, no evidence for increased efficacy).

A21(i): Task Force Members, Montalescot G, Sechtem U, Achenbach S, Andreotti F, Arden C, Budaj A, Bugiardini R, Crea F, Cuisset T, Di Mario C, Ferreira JR, Gersh BJ, Gitt AK, Hulot JS, Marx N, Opie LH, Pfisterer M, Prescott E, Ruschitzka F, Sabaté M, Senior R, Taggart DP, van der Wall EE, Vrints CJ; ESC Committee for Practice Guidelines, Zamorano JL, Achenbach S, Baumgartner H, Bax JJ, Bueno H, Dean V, Deaton C, Erol C, Fagard R, Ferrari R, Hasdai D, Hoes AW, Kirchhof P, Knuuti J, Kolh P, Lancellotti P, Linhart A, Nihoyannopoulos P, Piepoli MF, Ponikowski P, Sirnes PA, Tamargo JL, Tendera M, Torbicki A, Wijns W, Windecker S; Document Reviewers, Knuuti J, Valgimigli M, Bueno H, Claeys MJ, Donner-Banzhoff N, Erol C, Frank H, Funck-Brentano C, Gaemperli O, Gonzalez-Juanatey JR, Hamilos M, Hasdai D, Husted S, James SK, Kervinen K, Kolh P, Kristensen SD, Lancellotti P, Maggioni AP, Piepoli MF, Pries AR, Romeo F, Rydén L, Simoons ML, Sirnes PA, Steg PG, Timmis A, Wijns W, Windecker S, Yildirir A, Zamorano JL. 2013 ESC guidelines on the management of stable coronary artery disease: the Task Force on the management of stable coronary artery disease of the European Society of Cardiology. Eur Heart J. 2013 Oct;34(38):2949-3003.

A21(ii):Hennekens CH.Aspirin for the secondary prevention of atherosclerotic cardiovascular disease. In: UpToDate, Post, TW (Ed), UpToDate, Waltham, MA, 2019 last accessed date 23 October 2019

A21(iii):Cucchiara BL.Antiplatelet therapy for secondary prevention of stroke. In: UpToDate, Post, TW (Ed), UpToDate, Waltham, MA, 2019 last accessed date 23 October 2019

A21(iv):Spencer FA, Guyatt G, Tampi M, Golemiec B.Aspirin in the primary prevention of cardiovascular disease and cancer. In: UpToDate, Post, TW (Ed), UpToDate, Waltham, MA, 2019 last accessed date 23 October 2019

A21(v):Smith SC Jr, Benjamin EJ, Bonow RO, Braun LT, Creager MA, Franklin BA, Gibbons RJ, Grundy SM, Hiratzka LF, Jones DW, Lloyd-Jones DM, Minissian M, Mosca L, Peterson ED, Sacco RL, Spertus J, Stein JH, Taubert KA; World Heart Federation and the Preventive Cardiovascular Nurses Association. AHA/ACCF Secondary Prevention and Risk Reduction Therapy for Patients with Coronary and other Atherosclerotic Vascular Disease: 2011 update: a guideline from the American Heart Association and American College of Cardiology Foundation. Circulation. 2011 Nov 29;124(22):2458-73.

A21(Vİ): O'Mahony D, O'Sullivan D, Byrne S, O'Connor MN, Ryan C, Gallagher P. STOPP/START criteria for potentially inappropriate prescribing in older people: version 2. Age Ageing. 2015 Mar;44(2):213-8. doi: 10.1093/ageing/afu145. Epub 2014 Oct 16. Review.

A21(vii): Powers WJ, Rabinstein AA, Ackerson T, Adeoye OM, Bambakidis NC, Becker K,Biller J, Brown M, Demaerschalk BM, Hoh B, Jauch EC, Kidwell CS, Leslie-Mazwi TM,Ovbiagele B, Scott PA, Sheth KN, Southerland AM, Summers DV, Tirschwell DL;American Heart Association Stroke Council. 2018 Guidelines for the EarlyManagement of Patients With Acute Ischemic Stroke: A Guideline for HealthcareProfessionals From the American Heart Association/American Stroke Association.Stroke. 2018 Mar;49(3):e46-e110.

#### A22. Aspirin, clopidogrel, dipyridamole and OACs (Vitamin K antagonists, direct thrombin inhibitor or factor Xa inhibitors) with concurrent significant bleeding risk, i.e. uncontrolled severe hypertension, bleeding diathesis, recent non-trivial spontaneous bleeding) (high risk of bleeding).

A22(i): Lip GY. Implications of the CHA(2)DS(2)-VASc and HAS-BLED Scores for thromboprophylaxis in atrial fibrillation. Am J Med. 2011; 124(2):111-4.

A22(ii): Pisters R, Lane DA, Nieuwlaat R, de Vos CB, Crijns HJ, Lip GY. A novel user-friendly score (HAS-BLED) to assess 1-year risk of major bleeding in patients with atrial fibrillation: the Euro Heart Survey. Chest 2010; 138(5):1093-100.

A22(iii): O'Mahony D, O'Sullivan D, Byrne S, O'Connor MN, Ryan C, Gallagher P. STOPP/START criteria for potentially inappropriate prescribing in older people: version 2. Age Ageing. 2015 Mar;44(2):213-8. doi: 10.1093/ageing/afu145. Epub 2014 Oct 16. Review.

#### A23.Aspirin plus clopidogrel as secondary stroke prevention, if the specific indications for the combined use of aspirin and clopidogrel are not present.  **Aspirin + clopidogrel combination is appropriate in the following conditions:* *1. acute coronary syndrome or coronary intervention in the last 12 months (balloon and/ or stent)interventiondan sonra mi yazilsa?* *2. intervention for peripheric artery disease in the last 1 month* *stent placement (due to carotid artery stenosis/ lower extremity artery disease) in the last month* *balloon application to the lower limb in the last month* *3. stroke/ TIA in the last 3 weeks-3 months* *in patients with stroke or TIA due to intracranial atherosclerosis for 3 months* *in cases of minor stroke or TIA for 3 weeks*

A23(i): Diener HC, Bogousslavsky J, Brass LM, Cimminiello C, Csiba L, Kaste M, Leys D, Matias-Guiu J, Rupprecht HJ; MATCH investigators. Aspirin and clopidogrel compared with clopidogrel alone after recent ischaemic stroke or transient ischaemic attack in high-risk patients (MATCH): randomised, double-blind, placebo-controlled trial. Lancet 2004; 364(9431):331-7.

A23(ii): Bhatt DL, Fox KA, Hacke W, Berger PB, Black HR, Boden WE, Cacoub P, Cohen EA, Creager MA, Easton JD, Flather MD, Haffner SM, Hamm CW, Hankey GJ, Johnston SC, Mak KH, Mas JL, Montalescot G, Pearson TA, Steg PG, Steinhubl SR, Weber MA, Brennan DM, Fabry-Ribaudo L, Booth J, Topol EJ; CHARISMA Investigators. Clopidogrel and aspirin versus aspirin alone for the prevention of atherothrombotic events. N Engl J Med. 2006; 354(16):1706-17.

A23(iii): Usman MH, Notaro LA, Nagarakanti R, Brahin E, Dessain S, Gracely E, Ezekowitz MD. Combination antiplatelet therapy for secondary stroke prevention: enhanced efficacy or double trouble? Am J Cardiol 2009;103(8):1107-12. Review.

A23(iv): Squizzato A, Keller T, Romualdi E, Middeldorp S. Clopidogrel plus aspirin versus aspirin alone for preventing cardiovascular disease. Cochrane Database Syst Rev 2011;(1):CD005158. Review.

A23(v): Fares RR, Lansing LS, Gallati CA, Mousa SA. Antiplatelet therapy with clopidogrel and aspirin in vascular diseases: clinical evidence for and against the combination. Expert Opin Pharmacother 2008; 9(3): 377-86. Review.

A23(vi): O'Mahony D, O'Sullivan D, Byrne S, O'Connor MN, Ryan C, Gallagher P. STOPP/START criteria for potentially inappropriate prescribing in older people: version 2. Age Ageing. 2015 Mar;44(2):213-8. doi: 10.1093/ageing/afu145. Epub 2014 Oct 16. Review.

A23(vii):Valgimigli M. The ESC DAPT Guidelines 2017. Eur Heart J. 2018 Jan 14;39(3):187-188. doi: 10.1093/eurheartj/ehx768.

A23(viii): Aboyans V, Ricco JB, Bartelink MEL, Björck M, Brodmann M, Cohnert T, Collet JP, Czerny M, De Carlo M, Debus S, Espinola-Klein C, Kahan T, Kownator S, Mazzolai L, Naylor AR, Roffi M, Röther J, Sprynger M, Tendera M, Tepe G, Venermo M, Vlachopoulos C, Desormais I; ESC Scientific Document Group . 2017 ESC Guidelines on the Diagnosis and Treatment of Peripheral Arterial Diseases, in collaboration with the European Society for Vascular Surgery (ESVS): Document covering atherosclerotic disease of extracranial carotid and vertebral, mesenteric, renal, upper and lower extremity arteriesEndorsed by: the European Stroke Organization (ESO)The Task Force for the Diagnosis and Treatment of Peripheral Arterial Diseases of the European Society of Cardiology (ESC) and of the European Society for Vascular Surgery (ESVS). Eur Heart J. 2018 Mar 1;39(9):763-816. doi: 10.1093/eurheartj/ehx095.

#### A24. Aspirin/clopidogrel add on therapy in patients using OAC for chronic atrial fibrillation or for other reasons if there is no additional indication for aspirin/clopidogrel use (no added benefit from aspirin). **In patients with OAC use, it is appropriate to add aspirin/clopidogrel to the treatment in the following conditions:* *1. acute coronary syndrome or coronary intervention in the last 12 months (balloon and/ or stent)* *2. intervention for peripheric artery disease in the last 1 month* *stent placement (due to carotid artery stenosis / lower extremity artery disease) in the last month* *balloon application to the lower limb in the last month* **In patients with OAC use, it is not appropriate to add aspirin/clopidogrel to the treatment in the following conditions:* *1. peripheric artery disease not documented above (e.g. carotid artery stenosis, lower extremity artery disease, intracerebral atherosclerosis)* *2. stable coronary artery disease (e.g. acute coronary syndrome or coronary interventions more than 12 months ago)*

A24(i): Aboyans V, Ricco JB, Bartelink MEL, Björck M, Brodmann M, Cohnert T, Collet JP, Czerny M, De Carlo M, Debus S, Espinola-Klein C, Kahan T, Kownator S, Mazzolai L, Naylor AR, Roffi M, Röther J, Sprynger M, Tendera M, Tepe G, Venermo M, Vlachopoulos C, Desormais I; ESC Scientific Document Group. 2017 ESC Guidelines on the Diagnosis and Treatment of Peripheral Arterial Diseases, in collaboration with the European Society for Vascular Surgery (ESVS): Document covering atherosclerotic disease of extracranial carotid and vertebral, mesenteric, renal, upper and lower extremity arteriesEndorsed by: the European Stroke Organization (ESO)The Task Force for the Diagnosis and Treatment of Peripheral Arterial Diseases of the European Society of Cardiology (ESC) and of the European Society for Vascular Surgery (ESVS). Eur Heart J. 2018 Mar 1;39(9):763-816. doi: 10.1093/eurheartj/ehx095.

A24(ii): Flaker GC, Gruber M, Connolly SJ, Goldman S, Chaparro S, Vahanian A, Halinen MO, Horrow J, Halperin JL; SPORTIF Investigators. Risks and benefits of combining aspirin with anticoagulant therapy in patients with atrial fibrillation: an exploratory analysis of stroke prevention using an oral thrombin inhibitor in atrial fibrillation (SPORTIF) trials. Am Heart J 2006; 152(5):967-73.

A24(iii): Larson RJ, Fisher ES. Should aspirin be continued in patients started on warfarin? J Gen Intern Med 2004; 19(8):879-86. Review.

A24(iv): Kirchhof P, Benussi S, Kotecha D, Ahlsson A, Atar D, Casadei B, Castella M, Diener HC, Heidbuchel H, Hendriks J, Hindricks G, Manolis AS, Oldgren J, Popescu BA, Schotten U, Van Putte B, Vardas P, Agewall S, Camm J, Baron Esquivias G,Budts W, Carerj S, Casselman F, Coca A, De Caterina R, Deftereos S, Dobrev D, Ferro JM, Filippatos G, Fitzsimons D, Gorenek B, Guenoun M, Hohnloser SH, Kolh P,Lip GY, Manolis A, McMurray J, Ponikowski P, Rosenhek R, Ruschitzka F, Savelieva I, Sharma S, Suwalski P, Tamargo JL, Taylor CJ, Van Gelder IC, Voors AA, Windecker S, Zamorano JL, Zeppenfeld K. 2016 ESC Guidelines for the management of atrial fibrillation developed in collaboration with EACTS. Europace. 2016 Nov;18(11):1609-1678.)

# A24(v):XU H. Antithrombotic Therapy for Patients With Both Stable Coronary Artery Disease andAtrial Fibrillation-Expert Analysis 2014. Available at:

# <http://www.acc.org/latest-in-cardiology/articles/2014/07/18/15/34/antithrombotic-therapy-for-patients-with-both-stable-cad-and-afib>) (last accessed date 28 October 2019)

A24(vi): O'Mahony D, O'Sullivan D, Byrne S, O'Connor MN, Ryan C, Gallagher P. STOPP/START criteria for potentially inappropriate prescribing in older people: version 2. Age Ageing. 2015 Mar;44(2):213-8. doi: 10.1093/ageing/afu145. Epub 2014 Oct 16. Review.

# A24(vii): Camm AJ, Lip GY, De Caterina R, Savelieva I, Atar D, Hohnloser SH, HindricksG, Kirchhof P; ESC Committee for Practice Guidelines-CPG; Document Reviewers.2012 focused update of the ESC Guidelines for the management of atrialfibrillation: an update of the 2010 ESC Guidelines for the management of atrialfibrillation--developed with the special contribution of the European HeartRhythm Association. Europace. 2012 Oct;14(10):1385-413.

# A24(viii): Smith SC Jr, Benjamin EJ, Bonow RO, et al. AHA/ACCF Secondary Prevention and Risk Reduction Therapy for Patients with Coronary and other Atherosclerotic Vascular Disease: 2011 update: a guideline from the American Heart Association and American College of Cardiology Foundation. J Am Coll Cardiol 2011;58:2432-2446.

# A24(ix):Yasuda S, Kaikita K, Akao M, Ako J, Matoba T, Nakamura M, Miyauchi K, Hagiwara N, Kimura K, Hirayama A, Matsui K, Ogawa H; AFIRE Investigators. Antithrombotic Therapy for Atrial Fibrillation with Stable Coronary Disease. N Engl J Med. 2019 Sep 19;381(12):1103-1113.

#### A25. OACs (vitamin K antagonists, direct thrombin inhibitors or factor Xa inhibitors) for first deep venous thrombosis without continuing provoking risk factors (e.g. thrombophilia) for> 6 months (no proven added benefit).

A25 (i): Pinede L, Ninet J, Duhaut P, Chabaud S, Demolombe-Rague S, Durieu I, Nony P, Sanson C, Boissel JP; Investigators of the "Durée Optimale du Traitement AntiVitamines K" (DOTAVK) Study. Comparison of 3 and 6 months of oral anticoagulant therapy after a first episode of proximal deep vein thrombosis or pulmonary embolism and comparison of 6 and 12 weeks of therapy after isolated calf deep vein thrombosis. Circulation 2001; 103(20): 2453-60.

A25(ii): Kearon C, Akl EA, Comerota AJ, Prandoni P, Bounameaux H, Goldhaber SZ, Nelson ME, Wells PS, Gould MK, Dentali F, Crowther M, Kahn SR; American College of Chest Physicians. Antithrombotic therapy for VTE disease: Antithrombotic Therapy and Prevention of Thrombosis, 9th ed: American College of Chest Physicians Evidence-Based Clinical Practice Guidelines. Chest 2012; 141(2 Suppl): e419S-94S.

A25(iii):Lip GYH, Hull RD. Overview of the treatment of lower extremity deep vein thrombosis (DVT). In: UpToDate, Post, TW (Ed), UpToDate, Waltham, MA, 2019 last accessed date 23 October 2019

A25(iv):O'Mahony D, O'Sullivan D, Byrne S, O'Connor MN, Ryan C, Gallagher P. STOPP/START criteria for potentially inappropriate prescribing in older people: version 2. Age Ageing. 2015 Mar;44(2):213-8. doi: 10.1093/ageing/afu145. Epub 2014 Oct 16. Review.

#### A26. OACs (vitamin K antagonists, direct thrombin inhibitors or factor Xa inhibitors) for first pulmonary embolism without continuing provoking risk factors (e.g. thrombophilia) for > 12 months (no proven added benefit).

A26 (i): Kearon C, Akl EA, Comerota AJ, Prandoni P, Bounameaux H, Goldhaber SZ, Nelson ME, Wells PS, Gould MK, Dentali F, Crowther M, Kahn SR; American College of Chest Physicians. Antithrombotic therapy for VTE disease: Antithrombotic Therapy and Prevention of Thrombosis, 9th ed: American College of Chest Physicians Evidence-Based Clinical Practice Guidelines. Chest 2012; 141(2Suppl): e419S-94S.

A26(ii):TapsonVF, Weinberg AS.Treatment, prognosis, and follow-up of acute pulmonary embolism in adults. In: UpToDate, Post, TW (Ed), UpToDate, Waltham, MA, 2019 last accessed date 23 October 2019

A26(iii): O'Mahony D, O'Sullivan D, Byrne S, O'Connor MN, Ryan C, Gallagher P. STOPP/START criteria for potentially inappropriate prescribing in older people: version 2. Age Ageing. 2015 Mar;44(2):213-8. doi: 10.1093/ageing/afu145. Epub 2014 Oct 16. Review.

#### A27. Aspirin or clopidogrel monotherapy in patients with chronic atrial fibrillation who has contraindication for OAC (vitamin K antagonists, direct thrombin inhibitors or factor Xa inhibitors) use.

#### *Aspirin or clopidogrel monotherapy is not recommended for the prevention of stroke in patients with atrial fibrillation (harmful). **Dual antiplatelet therapy is not also recommended for patients with OAC contraindication due to the presence of bleeding risk.* **Left atrial appendage closure may be recommended in cases with high risk of stroke.*

A27(i):Kirchhof P, Benussi S, Kotecha D, Ahlsson A, Atar D, Casadei B, Castella M, Diener HC, Heidbuchel H, Hendriks J, Hindricks G, Manolis AS, Oldgren J, Popescu BA, Schotten U, Van Putte B, Vardas P; ESC Scientific Document Group. 2016 ESC Guidelines for the management of atrial fibrillation developed in collaboration with EACTS. Eur Heart J. 2016 Oct 7;37(38):2893-2962. doi:10.1093/eurheartj/ehw210. Epub 2016 Aug 27.

A27 (ii): Själander S, Själander A, Svensson PJ, Friberg L. Atrial fibrillation patients do not benefit from acetylsalicylic acid. Europace 2014; 16:631-8.

A27(iii): Olesen JB, Lip GY, Lindhardsen J, Lane DA, Ahlehoff O, Hansen ML, Raunsø J,Tolstrup JS, Hansen PR, Gislason GH, Torp-Pedersen C. Risks of thromboembolism and bleeding with thromboprophylaxis in patients with atrial fibrillation: A net clinical benefit analysis using a 'real world' nationwide cohort study. Thromb Haemost. 2011 Oct;106(4):739-49.

A27(iv):ManningWJ, Singer DE,Lip GYH. Atrial fibrillation: Anticoagulant therapy to prevent thromboembolism. In: UpToDate, Post, TW (Ed), UpToDate, Waltham, MA, 2019 last accessed date 23 October 2019

#### A28. Dabigatran if eGFR<30 ml/min/1.73 m2. *I*f eGFR <15 ml / min / 1.73 m2, NOACs should not be used.* ** Although there is limited evidence for apixaban, rivaroxaban and edoxaban in patients with eGFR 15-30 ml/min/1.73 m2, their use is not recommended.* ** In patients with atrial fibrillation, if eGFR<30 ml/min/ 1.73 m2, or have high risk of falls or had had life-threatening bleeding, in patients who do not want to take the risk of bleeding, in patients who have difficulty in INR control, in poorly controlled cases of hypertension anticoagulants may not be given.* **Anticoagulants are not recommended in patients on dialysis* [*unless there is a high risk of stroke: atrial thrombus, TIA-stroke, valvular disease (moderate-severe mitral stenosis, prosthetic valve)*].*If anticoagulation is indicated, warfarin should be used.* **In cases with high risk of stroke, closure of the left atrium appendix may be recommended if OACs cannot be used.* **Edoxaban should not be used if eGFR> 95 ml/min/1.73 m2.*

A28(i): Hariharan S, Madabushi R. Clinical pharmacology basis of deriving dosing recommendations for dabigatran in patients with severe renal impairment. J ClinPharmacol 2012; 52(1 Suppl):119S-25S.

A28(ii): Samama MM. Use of low-molecular-weight heparins and new anticoagulants in elderly patients with renal impairment. Drugs Aging 2011; 28(3): 177-93.

A28(iii): By the 2019 American Geriatrics Society Beers Criteria® Update Expert Panel. American Geriatrics Society 2019 Updated AGS Beers Criteria® for Potentially Inappropriate Medication Use in Older Adults. J Am Geriatr Soc. 2019Apr;67(4):674-694.

A28(iv): Kirchhof P, Benussi S, Kotecha D, Ahlsson A, Atar D, Casadei B, Castella M, Diener HC, Heidbuchel H, Hendriks J, Hindricks G, Manolis AS, Oldgren J, Popescu BA, Schotten U, Van Putte B, Vardas P; ESC Scientific Document Group. 2016 ESC Guidelines for the management of atrial fibrillation developed in collaboration with EACTS. Eur Heart J. 2016 Oct 7;37(38):2893-2962.

A28(v): Manning WJ, Singer DE, Lip GYH. Atrial fibrillation: Anticoagulant therapy to prevent thromboembolism. In: UpToDate, Post, TW (Ed), UpToDate, Waltham, MA, 2019 last accessed date 28 October 2019

A28(vi): O'Mahony D, O'Sullivan D, Byrne S, O'Connor MN, Ryan C, Gallagher P. STOPP/START criteria for potentially inappropriate prescribing in older people: version 2. Age Ageing. 2015 Mar;44(2):213-8. doi: 10.1093/ageing/afu145. Epub 2014 Oct 16. Review.

A28(vii) Steffel J, Verhamme P, Potpara TS, Albaladejo P, Antz M, Desteghe L, Georg Haeusler K, Oldgren J, Reinecke H, Roldan-Schilling V, Rowell N, Sinnaeve P, Collins R, Camm AJ, Heidbüchel H; ESC Scientific Document Group. The 2018 European Heart Rhythm Association Practical Guide on the use of non-vitamin K antagonist oral anticoagulants in patients with atrial fibrillation: executive summary. Europace. 2018 Aug 1;20(8):1231-1242.

#### A29. Warfarin in non-valvular atrial fibrillation if malnutrition or irregular food intake is present.

A29(i): Onder G, Landi F, Fusco D, Corsonello A, Tosato M, Battaglia M, Mastropaolo S, Settanni S, Antocicco M, Lattanzio F. Recommendations to prescribe in complex older adults: results of the CRIteria to assess appropriate Medication use among Elderly complex patients (CRIME) project. Drugs Aging. 2014 Jan;31(1):33-45.Review.

A29(ii): Lurie Y, Loebstein R, Kurnik D, Almog S, Halkin H. Warfarin and vitamin K intake in the era of pharmacogenetics. Br J Clin Pharmacol. 2010;70:164–70. 1092.

A29(iii): Sebastian JL, Tresch DD. Use of oral anticoagulants in older patients. Drugs Aging. 2000;16:409–35. 1094.

A29(iv): Ansell J, Hirsh J, Hylek E, Jacobson A, Crowther M, Palareti G. Pharmacology and management of the vitamin K antagonists: American College of Chest Physicians Evidence-Based Clinical Practice Guidelines (8th Edition). Chest. 2008 Jun;133(6 Suppl):160S-198S.

A29(v): Vranckx P, Valgimigli M, Heidbuchel H. The Significance of Drug-Drug and Drug-Food Interactions of Oral Anticoagulation. Arrhythm Electrophysiol Rev. 2018 Mar;7(1):55-61.

**A30. Narrow therapeutic indexed medications (such as warfarin, digoxin) in patients with known difficulty in managing therapy (e.g. patients with cognitive impairment) and lack of assistance (e.g., caregivers) (risk of life-threatening toxicity).**

A30(i): Onder G, Landi F, Fusco D, Corsonello A, Tosato M, Battaglia M, Mastropaolo S, Settanni S, Antocicco M, Lattanzio F. Recommendations to prescribe in complex older adults: results of the CRIteria to assess appropriate Medication use among Elderly complex patients (CRIME) project. Drugs Aging. 2014 Jan;31(1):33-45.Review.

A30(ii) van Deelen BA, van den Bemt PM, Egberts TC, van ’t Hoff A, Maas HA. Cognitive impairment as determinant for sub-optimal control of oral anticoagulation treatment in elderly patients with atrial fibrillation. Drugs Aging.2005;22(4):353-60. Review.

A30(iii): Diug B, Evans S, Lowthian J, Maxwell E, Dooley M, Street A, Wolfe R, Cameron P, McNeil J. The unrecognized psychosocial factors contributing to bleeding risk in warfarin therapy. Stroke. 2011 Oct;42(10):2866-71.

A30(iv): Arlt S, Lindner R, Ro¨sler A, von Renteln-Kruse W. Adherence to medication in patients with dementia: predictors and strategies for improvement. Drugs Aging. 2008;25:1033–47.

A30(v): Brauner DJ, Muir JC, Sachs GA. Treating nondementia illnesses in patients with dementia. JAMA. 2000;283:3230–5.

A30(vi): Marvanova M. Drug-induced cognitive impairment: Effect of cardiovascular agents. Ment Health Clin. 2016 Jun 29;6(4):201-206. doi: 10.9740/mhc.2016.07.201.

#### A31.Prasugrel in patients aged 75 years or older or had TIA/ stroke.

A31(i): Prasugrel: Druginformation, Lexicomp Online. Last accessed date 22 October 2019.

#### A32. Ticlopidine as an antiplatelet agent (clopidogrel or prasugrel or ticagrelor have higher efficacy, stronger evidence and fewer side-effects).

A32(i): Furie KL, Kasner SE, Adams RJ, Albers GW, Bush RL, Fagan SC, Halperin JL, Johnston SC, Katzan I, Kernan WN, Mitchell PH, Ovbiagele B, Palesch YY, Sacco RL, Schwamm LH, Wassertheil-Smoller S, Turan TN, Wentworth D; American Heart Association Stroke Council, Council on Cardiovascular Nursing, Council on Clinical Cardiology, and Interdisciplinary Council on Quality of Care and Outcomes Research. Guidelines for the prevention of stroke in patients with stroke or transient ischemic attack: a guideline for healthcare professionals from the American heart association/American stroke association. Stroke 2011; 42(1):227-76.

A32(ii): Porto I, Giubilato S, De Maria GL, Biasucci LM, Crea F. Platelet P2Y12 receptor inhibition by thienopyridines: status and future. Expert Opin Investig Drugs 2009; 18(9):1317-32. Review.

A32(iii): O'Mahony D, O'Sullivan D, Byrne S, O'Connor MN, Ryan C, Gallagher P. STOPP/START criteria for potentially inappropriate prescribing in older people: version 2. Age Ageing. 2015 Mar;44(2):213-8. doi: 10.1093/ageing/afu145. Epub 2014 Oct 16. Review.

A32(iv): Li YH, Fang CY, Hsieh IC, Huang WC, Lin TH, Sung SH, Chiu CZ, Wu CJ, Shyu KG, Chang PY, Fang CC, Lu TM, Chen CP, Tai WC, Sheu CC, Wei KC, Huang YH, Wu HM, Hwang JH. 2018 Expert Consensus on the Management of Adverse Effects of Antiplatelet Therapy for Acute Coronary Syndrome in Taiwan. *Acta Cardiol Sin*. 2018 May;34(3):201-210.

#### A33. Short-acting dipyridamole for antiplatelet-antiaggregant effect (orthostatic hypotension side effect and more effective agents).

A33(i): By the 2019 American Geriatrics Society Beers Criteria® Update Expert Panel. American Geriatrics Society 2019 Updated AGS Beers Criteria® for Potentially Inappropriate Medication Use in Older Adults. J Am Geriatr Soc. 2019Apr;67(4):674-694.

A33(ii): Dipyridamole: Drug information, Lexicomp Online. Last accessed date 22 October 2019.

#### A34. Statins for primary cardiovascular protection in patients with low-life expectancy (<2 years) or advanced dementia.

A34(i): Onder G, Landi F, Fusco D, Corsonello A, Tosato M, Battaglia M, Mastropaolo S, Settanni S, Antocicco M, Lattanzio F. Recommendations to prescribe in complex older adults: results of the CRIteria to assess appropriate Medication use among Elderly complex patients (CRIME) project. Drugs Aging. 2014 Jan;31(1):33-45.Review.

A34(ii):Pignone M. Management of elevated low density lipoprotein-cholesterol (LDL-C) in primary prevention of cardiovascular disease.In: UpToDate, Post, TW (Ed), UpToDate, Waltham, MA, 2019 last accessed date 23 October 2019

A34(iii): Lavan AH, Gallagher P, Parsons C, O'Mahony D. STOPPFrail (Screening Tool of Older Persons Prescriptions in Frail adults with limited life expectancy) consensus validation. Age Ageing. 2017 Jul 1;46(4):600-607.

#### A35. Allopurinol for asymptomatic hyperuricemia (those without gout or nephrolithiasis) (no evidence for benefit, risk of side effects with the use of xanthine oxidase inhibitors) (there is no evidence that treatment reduces cardiovascular risk or gout). **If serum uric acid>13 mg/dL in men or >10 mg/dl in women, uric-acid lowering therapy may be suggested due to risk of chronic kidney disease.*

A35(i): Feig DI, Kang DH, Johnson RJ. Uric acid and cardiovascular risk. N Engl J Med. 2008 Oct 23;359(17):1811-21. doi: 10.1056/NEJMra0800885. Review.

A35(ii):Poon SH, Hall HA, Zimmermann B. Approach to the treatment of hyperuricemia. Med Health R I. 2009 Nov;92(11):359-62. Review.

A35(iii): Maria Lorenza Muiesan, Claudia Agabiti-Rosei, Anna Paini, Massimo Salvetti. Uric Acid and Cardiovascular Disease: An Update. European Cardiology Review 2016;11(1):54–9.

A35(iv): Luis Ruilope, César Cerezo. Uric acid and cardiovascular risk considered: an update An article from the e-journal of the ESC Council for cardiology Practice. e-Journal of Cardiology Practice. Vol. 10, N° 21 - 02 Mar 2012. Available at:

<https://www.escardio.org/Journals/E-Journal-of-Cardiology-Practice/Volume-10/Uric-Acid-and-Cardiovascular-Risk-Considered-an-Update>; last accessed date 28 October 2019

A35(V):Becker MA, Perez-Ruiz F. Pharmacologic urate-lowering therapy and treatment of tophi in patients with gout. In: UpToDate, Post, TW (Ed), UpToDate, Waltham, MA, 2019 last accessed date 28 October 2019

A35(vi): Wallace SL, Singer JZ. Therapy in gout. Rheum Dis Clin North Am 1988; 14:441.

A35(vii): Becker MA, Mount DB.Asymptomatic hyperuricemia. In: UpToDate, Post, TW (Ed), UpToDate, Waltham, MA, 2019 last accessed date 23 October 2019

A35(viii): Fessel WJ. Renal outcomes of gout and hyperuricemia. Am J Med. 1979 Jul;67(1):74-82.

**Section B: Central Nervous System criteria.**

#### B1. Tricyclic antidepressants (high anticholinergic effect, cognitive side effects, cardiac conduction disorder, orthostatic hypotension, urinary retention, worsening of prostatism, worsening of narrow-angle glaucoma).

B1(i):By the 2019 American Geriatrics Society Beers Criteria® Update Expert Panel. American Geriatrics Society 2019 Updated AGS Beers Criteria® for Potentially Inappropriate Medication Use in Older Adults. J Am Geriatr Soc. 2019Apr;67(4):674-694.

B1(ii): Sultana J, Spina E, Trifirò G. Antidepressant use in the elderly: the role of pharmacodynamics and pharmacokinetics in drug safety. Expert Opin Drug Metab Toxicol. 2015 Jun;11(6):883-92. doi: 10.1517/17425255.2015.1021684. Epub 2015 Mar 3. Review.

#### B2. Paroxetine, fluoxetine and fluvoxamine as the first line treatment among SSRIs (due to high anticholinergic effect of paroxetine, long half-life of fluoxetine, frequent drug interaction with fluoxetine and fluvoxamine).

B2(i):Canadian Coalition for Seniors’ Mental Health. National guidelines for seniors’ mental health: The assessment and treatment of depression. Toronto, ON: Canadian Coalition for Seniors’ Mental Health; 2006. Available at: https://ccsmh.ca/wp-content/uploads/2016/03/NatlGuideline_Depression.pdf (last accessed date October 23, 2019).

B2(ii): Bonnie Wiese, MD, MA, FRCPC. Geriatric depression: The use of antidepressants in the elderly. BCMJ, Vol. 53, No. 47, September, 2011, Page(s) 341-347 - Clinical Articles. Available at:

http://www.bcmj.org/articles/geriatric-depression-use-antidepressants-elderly. (last accessed date October 23, 2019).

#### B3. SSRIs with current or recent significant hyponatremia i.e. serum Na<130 mEq/L (risk of exacerbating or precipitating hyponatremia). **Risk factors for the development of hyponatremia with SSRIs are advanced age, female gender, concomitant use of diuretics, low body weight and low basal Na value.* **Patients, who is started on SSRIs for the first time or is exposed to dose exposure, should be informed about the clinical findings of hyponatremia. The first 4 weeks is the most risky period.* **For patients with hyponatremia, mirtazapine or bupropion may be preferable instead of an SSRI.*

B3 (i): Jacob S, Spinler SA. Hyponatremia associated with selective serotonin-reuptake inhibitors in older adults. Ann Pharmacother 2006; 40(9):1618-22. Review.

B3 (ii): Draper B, Berman K. Tolerability of selective serotonin reuptake inhibitors: issues relevant to the elderly. Drugs Aging 2008; 25(6): 501-19. Review.

B3 (iii): By the 2019 American Geriatrics Society Beers Criteria® Update Expert Panel. American Geriatrics Society 2019 Updated AGS Beers Criteria® for Potentially Inappropriate Medication Use in Older Adults. J Am Geriatr Soc. 2019Apr;67(4):674-694.

B3(iv): O'Mahony D, O'Sullivan D, Byrne S, O'Connor MN, Ryan C, Gallagher P. STOPP/START criteria for potentially inappropriate prescribing in older people: version 2. Age Ageing. 2015 Mar;44(2):213-8. doi: 10.1093/ageing/afu145. Epub 2014 Oct 16. Review.

B3(v): Fabian TJ, Amico JA, Kroboth PD, et al. Paroxetine-induced hyponatremia in older adults: a 12-week prospective study. Arch Intern Med. 2004;164: 327–332.

B3(vi): Fiske A,Wetherell JL, Gatz M. Depression in older adults. Annu Rev Clin Psychol. 2009;5:363–389.

B3(vii): De Picker L,VanDen Eede F, Dumont G, et al.Antidepressants and the risk of hyponatremia: a class-by-class review of literature. Psychosomatics. 2014;55:536–547.

B3(viii): Leth-Møller KB, Hansen AH, Torstensson M, Andersen SE, Ødum L, Gislasson G,Torp-Pedersen C, Holm EA. Antidepressants and the risk of hyponatremia: a Danishregister-based population study. BMJ Open. 2016 May 18;6(5):e011200.

#### B4. SNRIs in patients with uncontrolled hypertension. **Side effects of venlafaxine are more prominent than duloxetine.* **Hypertension side-effect is more prominent with venlafaxine at doses> 300 mg/day.*

B4(i): Breeden M, Brieler J, Salas J, Scherrer JF. Antidepressants and Incident Hypertension in Primary Care Patients. J Am Board Fam Med. 2018 Jan-Feb;31(1):22-28.

B4(ii): Taylor D, Lenox-Smith A, Bradley A. A review of the suitability of duloxetine and venlafaxine for use in patients with depression in primary care with a focus on cardiovascular safety, suicide and mortality due to antidepressant overdose. Ther Adv Psychopharmacol. 2013 Jun;3(3):151-61.

B4 (iii): Thase ME. Effects of venlafaxine on blood pressure: a meta-analysis of original data from 3744 depressed patients. J Clin Psychiatry. 1998 Oct;59(10):502-8.

#### B5. Duloxetine if eGFR<30 ml/min/1.73m2 (increased GIS side effect).  **Use of other common antidepressants in case of renal failure:*

#### *Citalopram and escitalopram: dose adjustment is not necessary for mild to moderate renal failure. For serious renal failure (eGFR<20 ml/min/1.73 m2), dose adjustment is not necessary but should be used with caution.*

#### *Sertraline: dose adjustment is not necessary in renal failure.* *Paroxetine: for patients with eGFR<30 ml/min/1.73 m2, maximum dose for rapid release*

#### *tablets and extended release preparations are 40 mg/d and 50 mg/d, respectively.*

#### *Venlafaxine: If eGFR<30 ml/min/1.73 m2, the dose should be reduced by 50% for extended*

#### *release preparations; if the eGFR is 10-75 ml/min/1.73 m2, the dose should be reduced by 25% for fast-release preparations.*

#### *Mirtazapine: dose adjustment is not necessary in renal failure but should be used with*

#### *caution in moderate to severe renal failure.*

#### *Agomelatine: dose adjustment is not necessary in renal failure.* *Vortioxetine: dose adjustment is not necessary in renal failure.*

B5(i):By the 2019 American Geriatrics Society Beers Criteria® Update Expert Panel. American Geriatrics Society 2019 Updated AGS Beers Criteria® for Potentially Inappropriate Medication Use in Older Adults. J Am Geriatr Soc. 2019Apr;67(4):674-694.

B5(ii):Vortioxetine: Drug information, Lexicomp Online. Last accessed date 22 October 2019.

B5(iii):Paroxetine: Drug information, Lexicomp Online. Last accessed date 22 October 2019.

B5(iv):Duloxetine: Drug information, Lexicomp Online. Last accessed date 22 October 2019.

B5(v):Sertraline: Drug information, Lexicomp Online. Last accessed date 22 October 2019.

B5(vi):Mirtazapine: Drug information, Lexicomp Online. Last accessed date 22 October 2019.

B5(vii): Venlafaxine: Drug information, Lexicomp Online. Last accessed date 22 October 2019.

B5(viii):Escitalopram: Drug information, Lexicomp Online. Last accessed date 22 October 2019.

B5(ix):Citalopram: Drug information, Lexicomp Online. Last accessed date 22 October 2019.

#### B6. Pregabalin and gabapentin without dose reduction if eGFR<30 ml/min/1.73m2

B6(i): By the 2019 American Geriatrics Society Beers Criteria® Update Expert Panel. American Geriatrics Society 2019 Updated AGS Beers Criteria® for Potentially Inappropriate Medication Use in Older Adults. J Am Geriatr Soc. 2019Apr;67(4):674-694.

B6(ii):Pregabalin: Drug information, Lexicomp Online. Last accessed date 22 October 2019.

#### B7. High anticholinergic drugs in patients with delirium or dementia (amitriptyline, paroxetine, dicyclomine, l-hyoscyamine, thioridazine, chlorpromazine, clozapine, olanzapine, urinary antimuscarinics,H1 receptor blockers esp. 1st generation H1 receptor blockers (diphenhydramine, cyproheptadine, pheniramine), H2 receptor blockers (risk of cognitive deterioration).

#### **Clozapine may need to be used in clinical practice for the treatment of severe behavioral symptoms of dementia/ delirium in patients with extrapyramidal system symptoms (signs of parkinsonism) such as Parkinson's dementia or Lewy body dementia. In these cases, it should be used as short as possible, at the lowest possible dose and with close follow-up of cognitive function.* **In patients with extrapyramidal system symptoms (signs of parkinsonism) such as dementia with Parkinson's disease or Lewy body dementia (signs of parkinsonism), it is appropriate to use quetiapine in the first step before clozapine treatment if neuroleptic use is required due to severe behavioral symptoms.*

#### **When considered, clozapine/ quetiapine treatments should be started at a dose of 12.5 mg, the side effects should be monitored closely and should be increased by 12.5-25 mg if needed. After a few weeks of symptom control, dose reduction/withdrawal should be considered.*

B7 (i): Pagoria D, O'Connor RC, Guralnick ML. Antimuscarinic drugs: review of the cognitive impact when used to treat overactive bladder in elderly patients. Curr Urol Rep 2011; 12 (5): 351-7. Review.

B7 (ii): Gerretsen P, Pollock BG. Drugs with anticholinergic properties: a current perspective on use and safety. Expert Opin Drug Saf 2011; 10(5): 751-65. Review.

B7(iii): O'Mahony D, O'Sullivan D, Byrne S, O'Connor MN, Ryan C, Gallagher P. STOPP/START criteria for potentially inappropriate prescribing in older people: version 2. Age Ageing. 2015 Mar;44(2):213-8. doi: 10.1093/ageing/afu145. Epub 2014 Oct 16. Review.

B7 (iv): McKeith IG, Boeve BF, Dickson DW, et al., Diagnosis and management of dementia with Lewy bodies: Fourth consensus report of the DLB Consortium. Neurology. 2017 Jul 4;89(1):88-100.

B7(v): By the 2019 American Geriatrics Society Beers Criteria® Update Expert Panel. American Geriatrics Society 2019 Updated AGS Beers Criteria® for Potentially Inappropriate Medication Use in Older Adults. J Am Geriatr Soc. 2019 Apr;67(4):674-694.

#### B8. Anticholinergic agents for the treatment of Parkinson's disease (increased risk of side effects, safer and more effective drugs available).

B8(i):Spindler MA, Tarsy D.Initial pharmacologic treatment of Parkinson disease. In: UpToDate, Post, TW (Ed), UpToDate, Waltham, MA, 2019 last accessed date 23 October 2019

B8(ii):Cummings JL. Behavioral complications of drug treatment of Parkinson's disease. J Am Geriatr Soc. 1991 Jul;39(7):708-16. Review.

#### B9. Anticholinergics/antimuscarinics to treat extrapyramidal side-effects of neuroleptic / antipsychotic medications (risk of anticholinergic toxicity)

B9(i): Heinik J. Effects of trihexyphenidyl on MMSE and CAMCOG scores of medicated elderly patients with schizophrenia. Int Psychogeriatr 1998; 10(1): 103-8.

B9(ii): Drimer T, Shahal B, Barak Y. Effects of discontinuation of long-term anticholinergic treatment in elderly schizophrenia patients. Int ClinPsychopharmacol 2004; 19(1):27-9.

B9(iii): O'Mahony D, O'Sullivan D, Byrne S, O'Connor MN, Ryan C, Gallagher P. STOPP/START criteria for potentially inappropriate prescribing in older people: version 2. Age Ageing. 2015 Mar;44(2):213-8. doi: 10.1093/ageing/afu145. Epub 2014 Oct 16. Review.

B9(iv): By the 2019 American Geriatrics Society Beers Criteria® Update Expert Panel. American Geriatrics Society 2019 Updated AGS Beers Criteria® for Potentially Inappropriate Medication Use in Older Adults. J Am Geriatr Soc. 2019 Apr;67(4):674-694.

#### B10. Neuroleptics/ antipsychotics in patients with behavioral and psychological symptoms of dementia (BPSD) unless symptoms are severe and non-pharmacological treatments have failed (increased risk of stroke, heart failure, pneumonia-infection, risk of death). **Neuroleptic antipsychotic can be used in patients with BPSD when symptoms are severe and other non-pharmacological treatments have failed but it should be used at the lowest dose and for shortest time.* **In dementia patients, the primary approach in the management of BPSD is to provide optimum dementia therapy (ChEIs/ memantine).  Subsequently SSRIs (especially citalopram) can be tested.* **There is conflicting information about the efficacy of sertraline, trazodone, and melatonin for BPSD.*

B10(i):Desmidt T, Hommet C, Camus V. Pharmacological treatments of behavioral and psychological symptoms of dementia in Alzheimer's disease: role of acetylcholinesterase inhibitors and memantine. Geriatr Psychol Neuropsychiatr Vieil. 2016 Sep 1;14(3):300-6. doi: 10.1684/pnv.2016.0621. Review.

B10(ii): Campbell N, Ayub A, Boustani MA, Fox C, Farlow M, Maidment I, Howards R. Impact of cholinesterase inhibitors on behavioral and psychological symptoms of Alzheimer's disease: a meta-analysis. Clin Interv Aging. 2008;3(4):719-28.

B10(iii): Tible OP, Riese F, Savaskan E, von Gunten A. Best practice in the management of behavioural and psychological symptoms of dementia. Ther Adv Neurol Disord. 2017 Aug;10(8):297-309. doi: 10.1177/1756285617712979. Epub 2017 Jun 19. Review.

B10(iv):Press D, Alexander M.Management of neuropsychiatric symptoms of dementia. In: UpToDate, Post, TW (Ed), UpToDate, Waltham, MA, 2019 last accessed date 23 October 2019.

B10(v): Corbett A, Smith J, Creese B, Ballard C. Treatment of behavioral and psychological symptoms of Alzheimer's disease. Curr Treat Options Neurol. 2012 Apr;14(2):113-25. doi: 10.1007/s11940-012-0166-9.

B10(vi): Whitney M. Buterbaugh, Todd Jamrose, Jonathon Lazzara, Lindsay Honaker, and Christopher J. Thomas (2014) Review of antidepressants in the treatment**of** behavioral and psychiatric symptoms in dementia (BPSD)Mental Health Clinician: July 2014, Vol. 4, pp 183-188.

B10(vii):Henry G, Williamson D, Tampi RR. Efficacy and tolerability of antidepressants in the treatment of behavioral and psychological symptoms of dementia, a literature review of evidence. Am J Alzheimers Dis Other Demen. 2011 May;26(3):169-83. doi: 10.1177/1533317511402051. Epub 2011 Mar 23. Review.

B10(viii): Seitz DP, Adunuri N, Gill SS, Gruneir A, Herrmann N, Rochon P. Antidepressantsfor agitation and psychosis in dementia. Cochrane Database Syst Rev. 2011 Feb16;(2):CD008191. doi: 10.1002/14651858.CD008191.pub2. Review.

B10(ix): Hersch EC, Falzgraf S. Management of the behavioral and psychological symptoms of dementia. Clin Interv Aging. 2007;2(4):611-21.

B10(x): Sultzer DL, Gray KF, Gnay I, Berisford MA, Mahler ME. A double-blind comparison of trazodone and haloperidol for treatment of agitation in patients with dementia. Am J Geriatr Psychiatry. 1997 Winter;5(1):60-9.

B10 (xi): Pazan F, Weiss C, Wehling M; FORTA. The EURO-FORTA (Fit fOR The Aged) List: International Consensus Validation of a Clinical Tool for Improved Drug Treatment in Older People. Drugs Aging. 2018 Jan;35(1):61-71.

B10 (xii): Martinon-Torres G, Fioravanti M, Grimley EJ. Trazodone for agitation indementia. Cochrane Database Syst Rev. 2004 Oct 18;(4):CD004990. Review.

B10(xiii):Alagiakrishnan K. Melatonin based therapies for delirium and dementia. Discov Med. 2016 May;21(117):363-71. Review.

B10(xiv): De Jonghe A, Korevaar JC, Van Munster BC, De Rooij SE. Effectiveness of melatonin treatment on circadian rhythm disturbances in dementia. Are there implications for delirium? A systematic review. Int J Geriatr Psychiatry 2010; 25(12):1201– 1208.

B10(xv): Jansen SL, Forbes DA, Duncan V, Morgan DG: Melatonin for cognitive impairment. Cochrane Database Syst Rev 2006; Jan 25 (1):CD003802. Review.

B10(xvi): Rabins P, Rovner B, Rummans T, Schneider L, Tariot P. GUIDELINE WATCH (OCTOBER 2014): PRACTICE GUIDELINE FOR THE TREATMENT OF PATIENTS WITH ALZHEIMER’S DISEASE AND OTHER DEMENTIAS. Available at: <https://psychiatryonline.org/pb/assets/raw/sitewide/practice_guidelines/guidelines/alzheimerwatch.pdf>(last accessed date 23 October 2019)

B10(xvii): O'Mahony D, O'Sullivan D, Byrne S, O'Connor MN, Ryan C, Gallagher P. STOPP/START criteria for potentially inappropriate prescribing in older people: version 2. Age Ageing. 2015 Mar;44(2):213-8. doi: 10.1093/ageing/afu145. Epub 2014 Oct 16. Review.

#### B11. Neuroleptics/antipsychotics for hypnotic purpose (increased confusion, hypotension, extrapyramidal side effects, risk of fall). **Sleep hygiene and cognitive behavioral therapy should be applied at first hand in the treatment of insomnia in older adults.* **If pharmacological treatment is needed in the treatment of insomnia in older adults; melatonin or melatonin receptor agonist ramelteon may be used. Use of sedative antidepressants (mirtazapine, trazodone, agomelatine) can be considered if there is associated depression.*

B11 (i): British National Formulary vol. 76, September 2018-March 2019: p 28.

B11 (ii): RD McEvoy, KS Nyfort-Hansen. Sleep disorders in the elderly: the pros and cons of prescribing. In: Prescribing for Elderly Patients, eds. S. Jackson, P. Jansen, A. Mangoni. Wiley-Blackwell 2009, pp 45-52.

B11 (iii): Alexopoulos GS, Streim J, Carpenter D, Docherty JP. Expert Consensus Panel for Using Antipsychotic Drugs in Older Patients. Using antipsychotic agents in older patients. J Clin Psychiatry 2004; 65 Suppl 2:5-99; discussion 100-102; quiz 103-4. Review.

B11 (iv): O'Mahony D, O'Sullivan D, Byrne S, O'Connor MN, Ryan C, Gallagher P. STOPP/START criteria for potentially inappropriate prescribing in older people: version 2. Age Ageing. 2015 Mar;44(2):213-8. doi: 10.1093/ageing/afu145. Epub 2014 Oct 16. Review.

B11(v): Schroeck JL, Ford J, Conway EL, Kurtzhalts KE, Gee ME, Vollmer KA, Mergenhag KA. Review of Safety and Efficacy of Sleep Medicines in Older Adults. Clin Ther. 2016 Nov;38(11):2340-2372.

#### B12. Neuroleptics/ antipsychotics (i.e. other than quetiapine or clozapine) in those with parkinsonism or Lewy Body Disease (risk of severe extrapyramidal symptoms).

B12(i): Mena MA, de Yébenes JG. Drug-induced parkinsonism. Expert Opin Drug Saf 2006; 5(6):759-71. Review.

B12(ii): Eng ML, Welty TE. Management of hallucinations and psychosis in Parkinson's disease. Am J Geriatr Pharmacother 2010; 8(4):316-30. Review.

B12(iii): O'Mahony D, O'Sullivan D, Byrne S, O'Connor MN, Ryan C, Gallagher P. STOPP/START criteria for potentially inappropriate prescribing in older people: version 2. Age Ageing. 2015 Mar;44(2):213-8. doi: 10.1093/ageing/afu145. Epub 2014 Oct 16. Review.

B12(iv): McKeith IG, Boeve BF, Dickson DW, Halliday G, Taylor JP, Weintraub D, Aarsland D, Galvin J, Attems J, Ballard CG, Bayston A, Beach TG, Blanc F, Bohnen N, Bonanni L, Bras J, Brundin P, Burn D, Chen-Plotkin A, Duda JE, El-Agnaf O, Feldman H, Ferman TJ, Ffytche D, Fujishiro H, Galasko D, Goldman JG, Gomperts SN, Graff-Radford NR, Honig LS, Iranzo A, Kantarci K, Kaufer D, Kukull W, Lee VMY, Leverenz JB, Lewis S, Lippa C, Lunde A, Masellis M, Masliah E, McLean P, Mollenhauer B, Montine TJ, Moreno E, Mori E, Murray M, O'Brien JT, Orimo S, Postuma RB, Ramaswamy S, Ross OA, Salmon DP, Singleton A, Taylor A, Thomas A, Tiraboschi P, Toledo JB, Trojanowski JQ, Tsuang D, Walker Z, Yamada M, Kosaka K. Diagnosis and management of dementia with Lewy bodies: Fourth consensus report of the DLB Consortium. Neurology. 2017 Jul 4;89(1):88-100.

#### B13. Neuroleptics/antipsyhotics (may cause gait dyspraxia, parkinsonism), benzodiazepines (sedative, may cause reduced sensorium, impair balance) and Z-type hypnotic (e.g. zopiclone, zolpidem, zaleplon) (may cause protracted daytime sedation, ataxia) in patients with high fall risk.  **In general, these drugs increase the risk of falls in older adults. Use in older adults should be avoided.*

B13(i): Huang AR, Mallet L, Rochefort CM, Eguale T, Buckeridge DL, Tamblyn R. Medication-related falls in the elderly: causative factors and preventive strategies. Drugs Aging 2012; 29(5): 359-76. Review.

B13(ii): Woolcott JC, Richardson KJ, Wiens MO, Patel B, Marin J, Khan KM, Marra CA. Meta-analysis of the impact of 9 medication classes on falls in elderly persons. Arch Intern Med 2009; 169(21): 1952-60. Review. Erratum in: Arch Intern Med 2010 Mar 8;170(5):477.

B13(iii): Hill KD, Wee R. Psychotropic drug-induced falls in older people: a review of interventions aimed at reducing the problem. Drugs Aging 2012; 29(1): 15-30. Review.

B13(iv): Mets MA, Volkerts ER, Olivier B, Verster JC. Effect of hypnotic drugs on body balance and standing steadiness. Sleep Med Rev 2010; 14(4): 259-67.

B13(v): Shuto H, Imakyure O, Matsumoto J, Egawa T, Jiang Y, Hirakawa M, Kataoka Y, Yanagawa T. Medication use as a risk factor for inpatient falls in an acute care hospital: a case-crossover study. Br J Clin Pharmacol 2010; 69(5): 535-42.

B13(vi): O'Mahony D, O'Sullivan D, Byrne S, O'Connor MN, Ryan C, Gallagher P. STOPP/START criteria for potentially inappropriate prescribing in older people: version 2. Age Ageing. 2015 Mar;44(2):213-8. doi: 10.1093/ageing/afu145. Epub 2014 Oct 16. Review.

#### B14. Benzodiazepines for ≥ 4 weeks (risk of prolonged sedation, confusion, impaired balance, falls, road traffic accidents). *I*n general, the use of benzodiazepines should be avoided because of their high side effect potential. *Short-acting benzodiazepines can be used carefully and short-term (<4 weeks) in the presence of clinical indication (eg lorazepam in agitation associated with dementia).*

#### **Among long acting benzodiazepines, clonazepam may be used for REM sleep behavior disorder that is not controlled by other drugs in selected patients, but in this case it should be followed closely.* **All benzodiazepines used for 2 weeks or more should be discontinued gradually in order not to cause the benzodiazepine withdrawal syndrome.*

B14 (i): Madhusoodanan S, Bogunovic OJ. Safety of benzodiazepines in the geriatric population. Expert Opin Drug Saf 2004; 3(5): 485-93. Review.

B14 (ii): Glass J, Lanctôt KL, Herrmann N, Sproule BA, Busto UE. Sedative hypnotics inolder people with insomnia: meta-analysis of risks and benefits. BMJ 2005; 331(7526): 1169. Review.

B14 (iii): Barker MJ, Greenwood KM, Jackson M, Crowe SF. Cognitive effects of long-term benzodiazepine use: a meta-analysis. CNS Drugs 2004; 18(1):37-48.

B14 (iv): Model DG, Berry DJ. Effects of chlordiazepoxide in respiratory failure due to chronic bronchitis. Lancet 1974; 2(7885): 869-70.

B14 (v): Hak E, Bont J, Hoes AW, Verheij TJ. Prognostic factors for serious morbidity and mortality from community-acquired lower respiratory tract infections among the elderly in primary care. Fam Pract 2005; 22(4): 375-80.

B14(vi): O'Mahony D, O'Sullivan D, Byrne S, O'Connor MN, Ryan C, Gallagher P. STOPP/START criteria for potentially inappropriate prescribing in older people: version 2. Age Ageing. 2015 Mar;44(2):213-8. doi: 10.1093/ageing/afu145. Epub 2014 Oct 16. Review.

B14(vii): Kotagal V, Bohnen N I. Parkinson Disease and Related Disorders in Hazzards Geriatric Medicine and Gerontology Seventh edition. Eds. Halter J B, Ouslander J G, Studenski S, High K P, Asthana S, Ritchie C S, Supiano M A,; 2017 page 1431.

B14(viii): British National Formulary vol. 76, September 2018-March 2019: p 28.

#### B15. Benzodiazepines with acute or chronic respiratory failure i.e. PO2 <60 mmHg and / or PCO2> 50 mmHg (risk of exacerbation of respiratory failure).

B15 (i): Model DG, Berry DJ. Effects of chlordiazepoxide in respiratory failure due to chronic bronchitis. Lancet 1974; 2(7885): 869-70.

B15 (ii): Hak E, Bont J, Hoes AW, Verheij TJ. Prognostic factors for serious morbidity and mortality from community-acquired lower respiratory tract infections among the elderly in primary care. Fam Pract 2005; 22(4): 375-80.

B15 (iii): O'Mahony D, O'Sullivan D, Byrne S, O'Connor MN, Ryan C, Gallagher P. STOPP/START criteria for potentially inappropriate prescribing in older people: version 2. Age Ageing. 2015 Mar;44(2):213-8. doi: 10.1093/ageing/afu145. Epub 2014 Oct 16. Review.

B15 (iv): Overdyk FJ, Dowling O, Marino J, Qiu J, Chien HL, Erslon M, Morrison N, Harrison B, Dahan A, Gan TJ. Association of Opioids and Sedatives with Increased Risk of In-Hospital Cardiopulmonary Arrest from an Administrative Database. PLoS One. 2016 Feb 25;11(2):e0150214.

#### B16. ChEIs with a history of persistent bradycardia (<50/min), 2nd or 3rd degree heart block, recurrent unexplained syncope, prolonged QTc (> 470 ms in woman> 450 ms in men) (increased risk for heart conduction defect, syncope, injury risk). **ChEIs can be started in patients who have a heart rate of 50-60/min and are asymptomatic. Patients should be checked with regard to bradycardia and symptoms after 1 week of treatment initiation or dose increase.*

#### **In patients with concomitant rate-limiting medication, ChEIs can be used with caution if heart rate is not <50/min and there is no symptom. ChEIs should be used with caution in patients with left bundle branch block or atrial fibrillation, the patients should be monitered.* **ChEIs should be used with caution in patients with chronic obstructive pulmonary disease (COPD) or asthma. They could aggravate bronchospasm therefore should be monitered.* **ChEIs should be used with caution in patients with a history of gastric ulcer and those using nonsteroidal anti-inflammatory drugs. These patients should be monitored for gastrointestinal bleeding.*

B16(i): Salarbaks AM, Boomkamp-Snoeren CM, van Puijenbroek E, Jansen PA, van Marum RJ. [Cardiac effects of cholinesterase inhibitors: a reason for restraint?]. Tijdschr Gerontol Geriatr 2009; 40(2):79-84.

B16 (ii): [Fisher A.A.](http://0-www.embase.com.library.ucc.ie/search/results) and [Davis M.W.](http://0-www.embase.com.library.ucc.ie/search/results) Prolonged QT interval, syncope, and delirium with galantamine Ann Pharmacother 2008 42; 2: 278-283.

B16(iii): [Suleyman T](http://0-www.embase.com.library.ucc.ie/search/results), [Tevfik P](http://0-www.embase.com.library.ucc.ie/search/results), [Abdulkadir G.](http://0-www.embase.com.library.ucc.ie/search/results) and [Ozlem S.](http://0-www.embase.com.library.ucc.ie/search/results) Complete atrioventricular block and ventricular tachyarrhythmia associated with donepezil**.** Emerg Med J 2006; 23(8): 641-2.

B16(iv): [Bordier P](http://0-www.embase.com.library.ucc.ie/search/results), [Lanusse S](http://0-www.embase.com.library.ucc.ie/search/results), [Garrigue S](http://0-www.embase.com.library.ucc.ie/search/results), [Reynard C](http://0-www.embase.com.library.ucc.ie/search/results), [Robert F](http://0-www.embase.com.library.ucc.ie/search/results), [Gencel L](http://0-www.embase.com.library.ucc.ie/search/results) and [Lafitte A.](http://0-www.embase.com.library.ucc.ie/search/results) Causes of syncope in patients with Alzheimer's disease treated with donepezil. Drugs Aging 2005;  22(8): 687-694.

B16(v): O'Mahony D, O'Sullivan D, Byrne S, O'Connor MN, Ryan C, Gallagher P. STOPP/START criteria for potentially inappropriate prescribing in older people: version 2. Age Ageing. 2015 Mar;44(2):213-8. doi: 10.1093/ageing/afu145. Epub 2014 Oct 16. Review.

B16(vi): Helou R, Rhalimi M. Cholinesterase inhibitors and the risk of pulmonary disorders in hospitalized dementia patients. J Popul Ther Clin Pharmacol. 2010 Fall;17(3):e379-89. Epub 2010 Oct 26.

B16(vii): Thavorn K, Gomes T, Camacho X, Yao Z, Juurlink D, Mamdani M. Upper gastrointestinal bleeding in elderly adults with dementia receiving cholinesterase inhibitors: a population-based cohort study. J Am Geriatr Soc. 2014 Feb;62(2):382-4.

B16(viii):Rivastigmine: Drug information, Lexicomp Online. Last accessed date 22 October 2019.

#### B17. Levodopa or dopamine agonists for essential tremor (no evidence of efficacy)

B17(i): Zesiewicz TA, Elble RJ, Louis ED, Gronseth GS, Ondo WG, Dewey RB Jr, Okun MS, Sullivan KL, Weiner WJ. Evidence-based guideline update- treatment of essential tremor-report of the Quality Standards subcommittee of the American Academy of Neurology. Neurology 2011; 77(19):1752-5. Review.

B17 (ii): Deuschl G, Raethjen J, Hellriegel H, Elble R. Treatment of patients with essential tremor. Lancet Neurol 2011; 10(2): 148-61. Review.

B17(iii): O'Mahony D, O'Sullivan D, Byrne S, O'Connor MN, Ryan C, Gallagher P. STOPP/START criteria for potentially inappropriate prescribing in older people: version 2. Age Ageing. 2015 Mar;44(2):213-8. doi: 10.1093/ageing/afu145. Epub 2014 Oct 16. Review.

#### B18. Continuous and long-term use of betahistine, trimetazidine, dimenhydrinate in the treatment of vertigo (no evidence-based beneficial effect).

B18(i):FurmanJM, Barton JJS.Treatment of vertigo. In: UpToDate, Post, TW (Ed), UpToDate, Waltham, MA, 2019 last accessed date 28 October 2019.

B18(ii): Aman Nanda, Richard W. Besdine. Dizziness in Hazzards Geriatric Medicine and Gerontology Seventh edition. Eds. Halter J B, Ouslander J G, Studenski S, High K P, Asthana S, Ritchie C S, Supiano M A,; 2017 page 1086.

#### B19. Cinnarizine use (extrapyramidal side effects, limited use).

B19(i):Shin HW. Drug-induced parkinsonism. J Clin Neurol. 2012 Mar;8(1):15-21.

#### B20. Piracetam except for myoclonic convulsion therapy (with no proven clinical efficacy, cost burden and side effect potential). *Piracetam *can be used in patients who are believed to have symptomatic benefit from piracetam treatment, taking into account the benefit and harm balance.* **There are studies suggesting that piracetam may have limited benefit in the treatment of acute aphasia after stroke.*

B20(i):Piracetam for Aphasia in Post-stroke Patients: A Systematic Review and Meta-analysis of Randomized Controlled Trials. CNS Drugs. 2016 Jul;30(7):575-87.)

B20(ii):Wright CB. Treatment and prevention of vascular dementia. In: UpToDate, Post, TW (Ed), UpToDate, Waltham, MA, 2019 last accessed date 28 October 2019.

B20(iii).Flicker L, Grimley Evans G. Piracetam for dementia or cognitive impairment. Cochrane Database Syst Rev. 2001;(2):CD001011. Review.

#### B21. Carbamazepine, phenytoin, phenobarbital or valproate for chronic treatment of epilepsy as first step therapy (negative effects on vitamin D, enzyme induction, risk of falls, also safer alternatives available). **New agents such as levetiracetam, lamotrigine, gabapentin may be preferred in the treatment of chronic epilepsy in older adults.*

B21(i):Snih T. Seizures and epilepsy in older adults: Treatment and prognosis. In: UpToDate, Post, TW (Ed), UpToDate, Waltham, MA, 2019 last accessed date 23 October 2019.

B21(ii): Vestergaard P, Rejnmark L, Mosekilde L. Fracture risk associated with use of antiepileptic drugs. Epilepsia. 2004 Nov;45(11):1330-7.

B21(iii): Vestergaard P, Tigaran S, Rejnmark L, Tigaran C, Dam M, Mosekilde L. Fracture risk is increased in epilepsy. Acta Neurol Scand. 1999 May;99(5):269-75.

B21(iv): Koppel BS, Harden CL, Nikolov BG, Labar DR. An analysis of lifetime fractures in women with epilepsy. Acta Neurol Scand. 2005 Apr;111(4):225-8.

B21(v): Nakken KO, Sætre E, Markhus R, Lossius MI. [Epilepsy in the elderly]. Tidsskr Nor Laegeforen. 2013 Mar 5;133(5):528-31.

#### B22.Tramadol, neuroleptics/antipsychotics (clozapine, olanzapine, chlorpromazine, thioridazine), bupropion or maprotiline in epilepsy patients.

B22(i): By the American Geriatrics Society 2015 Beers Criteria Update Expert Panel.American Geriatrics Society 2015 Updated Beers Criteria for Potentially Inappropriate Medication Use in Older Adults. J Am Geriatr Soc. 2015 Nov;63(11):2227-46.

B22(ii): Habibi M, Hart F, Bainbridge J. The Impact of Psychoactive Drugs on Seizures and Antiepileptic Drugs. Curr Neurol Neurosci Rep. 2016 Aug;16(8):71.

#### B23. Antiepileptic treatment for seizure prophylaxis due to the presence of ischemic / hemorrhagic stroke in a patient without prior seizure.

B23(i): Hemphill JC 3rd, Greenberg SM, Anderson CS, Becker K, Bendok BR, Cushman M, Fung GL, Goldstein JN, Macdonald L, Mitchell PH, Scott PA, Selim MH, Woo D; American Heart Association Stroke Council; Council on Cardiovascular and Stroke Nursing; Council on Clinical Cardiology. Guidelines for the Management of Spontaneous Intracerebral Hemorrhage: A Guideline for Healthcare Professionals From the American Heart Association/American Stroke Association. Stroke. 2015Jul;46(7):2032-60.

B23(ii): Adams HP Jr, del Zoppo G, Alberts MJ, Bhatt DL, Brass L, Furlan A, Grubb RL,Higashida RT, Jauch EC, Kidwell C, Lyden PD, Morgenstern LB, Qureshi AI,Rosenwasser RH, Scott PA, Wijdicks EF; American Heart Association/American StrokeAssociation Stroke Council; American Heart Association/American StrokeAssociation Clinical Cardiology Council; American Heart Association/AmericanStroke Association Cardiovascular Radiology and Intervention Council;Atherosclerotic Peripheral Vascular Disease Working Group; Quality of CareOutcomes in Research Interdisciplinary Working Group. Guidelines for the earlymanagement of adults with ischemic stroke: a guideline from the American HeartAssociation/American Stroke Association Stroke Council, Clinical CardiologyCouncil, Cardiovascular Radiology and Intervention Council, and theAtherosclerotic Peripheral Vascular Disease and Quality of Care Outcomes inResearch Interdisciplinary Working Groups: The American Academy of Neurologyaffirms the value of this guideline as an educational tool for neurologists.Circulation. 2007 May 22;115(20):e478-534.

#### B24. Citalopram >20 mg / day and escitalopram >10 mg / day  (risk of QTc elongation).

B24(i): Citalopram: Drug information, Lexicomp Online. Last accessed date 22 October 2019.

B24(ii): U.S. Food and Drug Administration. Escitalopram: HIGHLIGHTS OF PRESCRIBING INFORMATION by FDA. Available at: <https://www.accessdata.fda.gov/drugsatfda_docs/label/2017/021323s047lbl.pdf> (last accessed date 28 October 2019.)

B24(iIi): October November reports of the European Pharmacovigilance Working Party. Drug Safety Update Vol 5 Issue 5, Dec 2011: A1.

**Section C: Gastrointestinal System criteria.**

#### C1. NSAIDs and OACs (vitamin K antagonist, direct thrombin inhibitor, factor Xa inhibitors) in combination (risk of gastrointestinal bleeding).

C1(i): Knijff-Dutmer EA, Van der Palen J, Schut G, Van de Laar MA. The influence of cyclo-oxygenase specificity of non-steroidal anti-inflammatory drugs on bleeding complications in concomitant coumarine users. QJM 2003; 96(7):513-20.

C1(ii): Peng S, Duggan A. Gastrointestinal adverse effects of non-steroidal anti-inflammatory drugs. Expert Opin Drug Saf 2005; 4(2):157-69. Review.

C1(iii): O'Mahony D, O'Sullivan D, Byrne S, O'Connor MN, Ryan C, Gallagher P. STOPP/START criteria for potentially inappropriate prescribing in older people: version 2. Age Ageing. 2015 Mar;44(2):213-8. doi: 10.1093/ageing/afu145. Epub 2014 Oct 16. Review.

C1(iv): Solomon DH. Nonselective NSAIDs: Overview of adverse effects.In: UpToDate, Post, TW (Ed), UpToDate, Waltham, MA, 2019 last accessed date 28 October 2019.

C1(v): Melcarne L, García-Iglesias P, Calvet X. Management of NSAID-associated peptic ulcer disease. Expert Rev Gastroenterol Hepatol. 2016 Jun;10(6):723-33.

C1(vi): Chinese Rheumatism Data Center; Chinese Systemic Lupus Erythematosus Treatment and Research Group. [Recommendation for the prevention and treatment of non-steroidal anti-inflammatory drug-induced gastrointestinal ulcers and its complications]. Zhonghua Nei Ke Za Zhi. 2017 Jan 1;56(1):81-85.

C1(vii): Masclee GM, Valkhoff VE, Coloma PM, de Ridder M, Romio S, Schuemie MJ, Herings R, Gini R, Mazzaglia G, Picelli G, Scotti L, Pedersen L, Kuipers EJ, van der Lei J, Sturkenboom MC. Risk of upper gastrointestinal bleeding from different drug combinations. Gastroenterology. 2014 Oct;147(4):784-792.e9; quiz e13-4.

#### C2. Aspirin, clopidogrel, NSAIDs or corticosteroids in patients with peptic ulcer history/dyspepsia-gastroesophageal reflux symptoms or with concurrent antiplatelet/anticoagulant/corticosteroid treatment(s) without PPI prophylaxis. **The recommended PPI dose for prophylaxis is the high dose of the preferred PPI once a day.*

#### **In the comorbid conditions other than dyspepsia, it is appropriate to continue with the high dose of PPI.*

#### **In indication due to dyspepsia, following a period of using high dose PPI, it may be appropriate to reduce the PPI to the lowest dose at which the patient's symptoms do not relapse in the follow-up*

#### **In older adults under chronic NSAID treatment, PPI is required even if there is no accompanying risk factor mentioned above.*

#### **In older adults using short-term NSAIDs, it may be appropriate to prescribe PPI simultaneously even if the above risk factors are not present.*

#### **In older adults using only aspirin or clopidogrel at antiaggregant dose, there is probably no need to use PPI if none of the above risk factors exist.*

#### **It is suggested that clopidogrel may be less effective in combination with PPIs. However, no clear recommendation exists in this regard.* **In patients using warfarin, omeprazole should not be used if there is PPI indication (omeprazole increases the level of warfarin).* **Instead of PPI therapy, misoprostol or high-dose H2 receptor blockers may also be used.*

C2(i): Lanza FL, Chan FK, Quigley EM; Practice Parameters Committee of the American College of Gastroenterology. Guidelines for prevention of NSAID-related ulcer complications. Am J Gastroenterol 2009; 104(3):728-38.

C2(ii): Nardulli G, Lanas A. Risk of gastrointestinal bleeding with aspirin and platelet antiaggregants. Gastroenterol Hepatol 2009; 32(1):36-43. Review.

C2(iii): Zullo A, Hassan C, Campo SM, Morini S. Bleeding peptic ulcer in the elderly-risk factors and prevention strategies. Drugs Aging 2007; 24(10): 815-28. Review.

C2(iv): Abraham NS, Hlatky MA, Antman EM, Bhatt DL, Bjorkman DJ, Clark CB, Furberg CD, Johnson DA, Kahi CJ, Laine L, Mahaffey KW, Quigley EM, Scheiman J, Sperling LS, Tomaselli GF; ACCF/ACG/AHA. ACCF/ACG/AHA 2010 expert consensus document on the concomitant use of proton pump inhibitors and thienopyridines: a focused update of the ACCF/ACG/AHA 2008 expert consensus document on reducing the gastrointestinal risks of antiplatelet therapy and NSAID use. Am J Gastroenterol. 2010 Dec;105(12):2533-49. doi: 10.1038/ajg.2010.445. Review.

C2(v): Vaduganathan M, Cannon CP, Cryer BL, Liu Y, Hsieh WH, Doros G, Cohen M, Lanas A, Schnitzer TJ, Shook TL, Lapuerta P, Goldsmith MA, Laine L, Bhatt DL; COGENT Investigators. Efficacy and Safety of Proton-Pump Inhibitors in High-Risk Cardiovascular Subsets of the COGENT Trial. Am J Med. 2016 Sep;129(9):1002-5.

C2(vi): Vaduganathan M, Bhatt DL, Cryer BL, Liu Y, Hsieh WH, Doros G, Cohen M, Lanas A, Schnitzer TJ, Shook TL, Lapuerta P, Goldsmith MA, Laine L, Cannon CP; COGENT Investigators. Proton-Pump Inhibitors Reduce Gastrointestinal Events Regardless of Aspirin Dose in Patients Requiring Dual Antiplatelet Therapy. J Am Coll Cardiol. 2016 Apr 12;67(14):1661-71.

C2(vii): O'Mahony D, O'Sullivan D, Byrne S, O'Connor MN, Ryan C, Gallagher P. STOPP/START criteria for potentially inappropriate prescribing in older people: version 2. Age Ageing. 2015 Mar;44(2):213-8. doi: 10.1093/ageing/afu145. Epub 2014 Oct 16. Review.

C2(viii):Feldman M, Das S.NSAIDs (including aspirin): Primary prevention of gastroduodenal toxicity. In: UpToDate, Post, TW (Ed), UpToDate, Waltham, MA, 2019 last accessed date 28 October 2019.

C2(ix): Bundhun PK, Teeluck AR, Bhurtu A, Huang WQ. Is the concomitant use of clopidogrel and Proton Pump Inhibitors still associated with increased adverse cardiovascular outcomes following coronary angioplasty?: a systematic review and meta-analysis of recently published studies (2012 - 2016). BMC Cardiovasc Disord. 2017 Jan 5;17(1):3. doi: 10.1186/s12872-016-0453-6. Review.

C2(x):Celebi A, Yilmaz H. When proton pump inhibitors are compared, are there specific cases in which a certain proton pump inhibitors should be particularly preferred? Turk J Gastroenterol. 2017 Dec;28(Suppl 1):S68-S70. doi: 10.5152/tjg.2017.17.

C2(xi): Sutfin T, Balmer K, Boström H, Eriksson S, Höglund P, Paulsen O. Stereoselective interaction of omeprazole with warfarin in healthy men. Ther Drug Monit. 1989;11(2):176-84.

C2(x): Satoh K, Yoshino J, Akamatsu T, Itoh T, Kato M, Kamada T, Takagi A, Chiba T,Nomura S, Mizokami Y, Murakami K, Sakamoto C, Hiraishi H, Ichinose M, Uemura N,Goto H, Joh T, Miwa H, Sugano K, Shimosegawa T. Evidence-based clinical practice guidelines for peptic ulcer disease 2015. J Gastroenterol. 2016 Mar;51(3):177-94.

C2(xi): British National Formulary vol. 76, September 2018-March 2019: p 1385.

#### C3. Initiation of chronic aspirin or NSAID use without testing for H. pylori in patients with a history of peptic ulcer (complicated or uncomplicated, gastric or duodenal). **If H. pylori is detectedpositive, eradication therapy should be given.* **If the patient has been taking aspirin/ NSAID for a while, the expected benefit from H. pylori eradication therapy is low. However, in practice, if it is screened and found positive, eradication is generally preferred.* **Even if the patient has not peptic ulcer history, in populations with high H. pylori prevalence, "test-treat" approach may be appropriate before initiation of chronic aspirin/NSAID use.* **In patients who will start chronic use of clopidogrel; even if a history of peptic ulcer (complicated or uncomplicated, gastric or duodenal) is present, H. pylori testing and/or H. pylori eradicationtherapy is not recommended (theoretical or in practical practice).*

C3(i):Feldman M, Das S. NSAIDs (including aspirin): Primary prevention of gastroduodenal toxicity. In: UpToDate, Post, TW (Ed), UpToDate, Waltham, MA, 2019 last accessed date 28 October 2019.

C3(ii): Yazıcı A, Akyuz F, Issever H, Pinarbasi B, Demir K, Ozdil S, Besisik F, Boztas G, Mungan ZA, Kaymakoğlu S, Peptic ulcer disease: Whay did change in Turkey? Gastroenterology 2008;134(4):A328-329.

C3(iii): Malfertheiner P, Megraud F, O'Morain CA, Atherton J, Axon AT, Bazzoli F, Gensini GF, Gisbert JP, Graham DY, Rokkas T, El-Omar EM, Kuipers EJ; European Helicobacter Study Group. Management of Helicobacter pylori infection—the Maastricht IV/ Florence Consensus Report. Gut. 2012 May;61(5):646-64.

C3 (iv): Kocazeybek B, Tokman HB. Prevalence of Primary Antimicrobial Resistance of H. pylori in Turkey: A Systematic Review. Helicobacter. 2016 Aug;21(4):251-60. doi: 10.1111/hel.12272. Epub 2015 Sep 23.

C3(v):Thung I, Aramin H, Vavinskaya V, Gupta S, Park JY, Crowe SE, Valasek MA. Review article: the global emergence of Helicobacter pylori antibiotic resistance. Aliment Pharmacol Ther. 2016 Feb;43(4):514-33. doi: 10.1111/apt.13497. Epub 2015 Dec 23. Review.

#### C4. PPIs for uncomplicated peptic ulcer disease or erosive peptic esophagitis at full therapeutic dose for> 8-12 weeks (dose reduction or earlier discontinuation indicated).

#### *The therapeutic dose duration of 8-12 weeks does not include the PPI treatment time for the H. pylori eradication.

#### *While discontinuing PPI treatment, it is appropriate to reduce the PPI dose gradually to avoid rebound effect (for example, a half-dose for 1-week, alternate days for 1 week, and discontinuation afterwards).

C4 (i): British National Formulary vol. 76, September 2018-March 2019: p 78-83.

C4 (ii): Gastro-oesophageal reflux disease and dyspepsia in adults: investigation and management. Clinical guideline. Published: 3 September 2014.Available at:

[www.nice.org.uk/guidance/cg184](http://www.nice.org.uk/guidance/cg184) last accessed date 29 October 2019.

C4(iii): O'Mahony D, O'Sullivan D, Byrne S, O'Connor MN, Ryan C, Gallagher P. STOPP/START criteria for potentially inappropriate prescribing in older people: version 2. Age Ageing. 2015 Mar;44(2):213-8. doi: 10.1093/ageing/afu145. Epub 2014 Oct 16. Review.

#### C5. PPIs for multiple drug use indication (no benefit, potential harm). **There is no “multiple drug use” indication among PPI indications. On the other hand, chronic use of PPI increases the risk of chronic kidney failure, fractures, dementia and the frequency of C. difficile infections, and is a risk factor for vitamin B12 deficiency, hypomagnesemia, and enteric infections-bacterial overgrowth.*

C5(i): Xie Y. et al. Proton Pump Inhibitors and Risk of Incident CKD and Progression to ESRD. J Am Soc Nephrol.2016 Oct;27(10):3153-3163.

C5(ii): Lazarus B. et al. Proton Pump Inhibitor Use and the Risk of Chronic Kidney Disease. JAMA Intern Med. 2016 Feb;176(2):238-46.;

C5(iii): Gomm W. et al. Association of Proton Pump Inhibitors With Risk of Dementia: A Pharmacoepidemiological Claims Data Analysis. JAMA Neurol. 2016 Apr;73(4):410-6.

C5(iv): Cai D, Feng W, Jiang Q. Acid-suppressive medications and risk of fracture: an updated meta-analysis. Int J Clin Exp Med. 2015 Jun 15;8(6):8893-904. eCollection 2015.

C5(v): Trifan A, Stanciu C, Girleanu I, Stoica OC, Singeap AM, Maxim R, Chiriac SA, Ciobica A, Boiculese L. Proton pump inhibitors therapy and risk of Clostridium difficile infection: Systematic review and meta-analysis. World J Gastroenterol. 2017 Sep 21;23(35):6500-6515. doi: 10.3748/wjg.v23.i35.6500. Review.

C5(vi): Scarpignato C, Gatta L, Zullo A, Blandizzi C; SIF-AIGO-FIMMG Group; Italian Society of Pharmacology, the Italian Association of Hospital Gastroenterologists, and the Italian Federation of General Practitioners. Effective and safe proton pump inhibitor therapy in acid-related diseases - A position paper addressing benefits and potential harms of acid suppression. BMC Med. 2016 Nov 9;14(1):179. Review.

#### C6. Anticholinergic GIS antispasmodics (e.g. hyoscyamine) [increased anticholinergic side effect in older adults (dizziness, decreased cognitive function, blurred vision, arrhythmia, flatulence-constipation) and limited benefit].

C6(i):By the 2019 American Geriatrics Society Beers Criteria® Update Expert Panel. American Geriatrics Society 2019 Updated AGS Beers Criteria® for Potentially Inappropriate Medication Use in Older Adults. J Am Geriatr Soc. 2019Apr;67(4):674-694.

C6(ii):Hyoscyamine: Drug information, Lexicomp Online. Last accessed date 22 October 2019.

C6(iii):Wald A.Treatment of irritable bowel syndrome in adults. In: UpToDate, Post, TW (Ed), UpToDate, Waltham, MA, 2019 last accessed date 23 October 2019.

#### C7. Drugs likely to cause constipation (e.g. antimuscarinic/anticholinergic drugs, oral iron, opioids, verapamil, aluminum antacids) in patients with chronic constipation where non-constipating alternatives are available (risk of exacerbation of constipation). **Calcium channel blocker antihypertensives other than verapamil may also cause constipation. However, this effect is more pronounced with verapamil and nifedipine in descending order.*

C7(i): Meek PD, Evang SD, Tadrous M, Roux-Lirange D, Triller DM, Gumustop B. Overactive bladder drugs and constipation: a meta-analysis of randomized, placebo-controlled trials. Dig Dis Sci 2011; 56(1): 7-18. Review.

C7 (ii): Müller-Lissner S. General geriatrics and gastroenterology: constipation and faecal incontinence. Best Pract Res Clin Gastroenterol 2002; 16(1): 115-33. Review.

C7 (iii): Harari D, Gurwitz JH, Avorn J, Choodnovskiy I, Minaker KL. Correlates of regular laxative use by frail elderly persons. Am J Med 1995; 99(5): 513-8.

C7 (iv): Opie LH. Choosing the correct drug for the individual hypertensive patient. Drugs 1992; 44 Suppl 1: 147-55. Review.

C7(v): Opie LH. Calcium channel antagonists. Part IV: Side effects and contraindications drug interactions and combinations. Cardiovasc Drugs Ther. 1988 Jul;2(2):177-89. Review.

C7(vi): Russell RP. Side effects of calcium channel blockers. Hypertension. 1988 Mar;11(3 Pt 2):II42-4. Review.

C7(vii): Poole-Wilson PA, Kirwan BA, Vokó Z, de Brouwer S, van Dalen FJ, Lubsen J; ACTION Investigators. Safety of nifedipine GITS in stable angina: the ACTION trial. Cardiovasc Drugs Ther. 2006 Feb;20(1):45-54.

C7(viii): Acosta A, Tangalos E G, Harari D. Constipation in Hazzards Geriatric Medicine and Gerontology Seventh edition. Eds. Halter J B, Ouslander J G, Studenski S, High K P, Asthana S, Ritchie C S, Supiano M A,; 2017. Page 1956.

C7(ix): O'Mahony D, O'Sullivan D, Byrne S, O'Connor MN, Ryan C, Gallagher P. STOPP/START criteria for potentially inappropriate prescribing in older people: version 2. Age Ageing. 2015 Mar;44(2):213-8. doi: 10.1093/ageing/afu145. Epub 2014 Oct 16. Review.

C7(x): Bulpitt CJ, Connor M, Schulte M, Fletcher AE. Bisoprolol and nifedipine retard in elderly hypertensive patients: effect on quality of life. J Hum Hypertens. 2000 Mar;14(3):205-12.

C7(xi): Elliott WJ, Ram CV. Calcium channel blockers. J Clin Hypertens (Greenwich).2011 Sep;13(9):687-9.

#### C8. Metoclopramide or trimethobenzamide as the first line antiemetic treatment of older adults (due to the extrapyramidal side effects and restlessness). **Serotonin 5-HT3 receptor antagonists are the safest antiemetic agents in older adults.* **Metoclopramide and trimethobenzamide are contraindicated in patients with Parkinson's disease.*

C8(i):Glare P, Miller J, Nikolova T, Tickoo R. Treating nausea and vomiting in palliative care: a review. Clin Interv Aging. 2011;6:243-59. doi: 10.2147/CIA.S13109. Epub 2011 Sep 12. Review.

C8(ii): Stephen PJ, Williamson J. Drug-induced parkinsonism in the elderly. Lancet 1984; 2(8411): 1082-3.

C8(iii): Ganzini L, Casey DE, Hoffman WF, McCall AL. The prevalence of metoclopramide-induced tardive dyskinesia and acute extrapyramidal movement disorders. Arch Intern Med 1993; 153(12): 1469-75.

C8(iv): Pasricha PJ, Pehlivanov N, Sugumar A, Jankovic J. Drug Insight: from disturbed motility to disordered movement - a review of the clinical benefits and medicolegal risks of metoclopramide. Nat Clin Pract Gastroenterol Hepatol 2006; 3(3): 138-48. Review.

C8(v): By the 2019 American Geriatrics Society Beers Criteria® Update Expert Panel. American Geriatrics Society 2019 Updated AGS Beers Criteria® for Potentially Inappropriate Medication Use in Older Adults. J Am Geriatr Soc. 2019Apr;67(4):674-694.

C8(vi): O'Mahony D, O'Sullivan D, Byrne S, O'Connor MN, Ryan C, Gallagher P. STOPP/START criteria for potentially inappropriate prescribing in older people: version 2. Age Ageing. 2015 Mar;44(2):213-8. doi: 10.1093/ageing/afu145. Epub 2014 Oct 16. Review.

#### C9. Magnesium preparations as laxative or antacid if eGFR<30 ml/min/1.73m2 (risk of hypermagnesemia).

C9(i):Yu ASL, Gupta A.Causes, symptoms, and treatment of hypermagnesemia. In: UpToDate, Post, TW (Ed), UpToDate, Waltham, MA, 2019 last accessed date 29 October 2019.

C9(ii): Navarro-González JF, Mora-Fernández C, García-Pérez J. Clinical implications of disordered magnesium homeostasis in chronic renal failure and dialysis. Semin Dial. 2009 Jan-Feb;22(1):37-44. doi: 10.1111/j.1525-139X.2008.00530.x. Review.

C9(iii):Rao SSC. Constipation in the older adult. In: UpToDate, Post, TW (Ed), UpToDate, Waltham, MA, 2019 last accessed date 29 October 2019.

C9(iv): Magnesium hydroxide: Drug information, Lexicomp Online. Last accessed date 22 October 2019.

C9(v): Magnesium carbonate: Drug information, Lexicomp Online. Last accessed date 22 October 2019.

**Section D: Respiratory System criteria.**

#### D1. Antimuscarinic bronchodilators (e.g. ipratropium, tiotropium) with a history of narrow angle glaucoma (may exacerbate glaucoma) or bladder outflow obstruction (may cause urinary retention). **Symptoms of obstructive lower urinary tract symptoms (LUTS) associated with benign prostatic hyperplasia are common in older men. In cases with mild obstructive symptoms, the clinician may use antimuscarinic bronchodilator drugs with close clinical follow-up. Patients with post-voiding residue (PVR)> 150 ml are particularly at risk for urinary retention and therefore antimuscarinic bronchodilator drugs should not be used if PVR> 150 ml.*

D1(i): Gupta P, O'Mahony MS. Potential adverse effects of bronchodilators in the treatment of airways obstruction in older people: recommendations for prescribing. Drugs Aging 2008; 25(5): 415-43. Review.

D1(ii): Oba Y, Zaza T, Thameem DM. Safety, tolerability and risk benefit analysis of tiotropium in COPD. Int J Chron Obstruct Pulmon Dis 2008; 3(4): 575-84. Review.

D1(iii): O'Mahony D, O'Sullivan D, Byrne S, O'Connor MN, Ryan C, Gallagher P. STOPP/START criteria for potentially inappropriate prescribing in older people: version 2. Age Ageing. 2015 Mar;44(2):213-8. doi: 10.1093/ageing/afu145. Epub 2014 Oct 16. Review.

D1(iv):Ah-Kee EY, Egong E, Shafi A, Lim LT, Yim JL. A review of drug-induced acute angle closure glaucoma for non-ophthalmologists. Qatar Med J. 2015 May 10;2015(1):6. doi: 10.5339/qmj.2015.6. eCollection 2015. Review.

D1(v):Vande Griend JP, Linnebur SA. Inhaled anticholinergic agents and acute urinary retention in men with lower urinary tract symptoms or benign prostatic hyperplasia. Ann Pharmacother. 2012 Sep;46(9):1245-9. doi: 10.1345/aph.1R282. Epub 2012 Jul 31. Review.

D1(vi): Carlos Andrés Celis Preciado, Horacio Giraldo, Dario Londoño, Ingrid Rodriguez. Glaucoma risk due to antimuscarinics, not a class effect: A systematic review. European Respiratory Journal 2016 48: PA4069.

D1(vii): British National Formulary vol. 76, September 2018-March 2019: p 247.

D1(viii): Hashimoto M, Hashimoto K, Ando F, Kimura Y, Nagase K, Arai K. Prescription rate of medications potentially contributing to lower urinary tract symptoms and detection of adverse reactions by prescription sequence symmetry analysis. J Pharm Health Care Sci. 2015 Feb 15;1:7.

#### D2. Theophylline in the maintenance treatment of COPD or asthma bronchiale (narrow therapeutic index, high insomnia and arrhythmia risk).

D2(i): Rabe KF, Hurd S, Anzueto A, Barnes PJ, Buist SA, Calverley P, Fukuchi Y, Jenkins C, Rodriguez-Roisin R, van Weel C, Zielinski J; Global Initiative for Chronic Obstructive Lung Disease. Global strategy for the diagnosis, management, and prevention of chronic obstructive pulmonary disease: GOLD executive summary. Am J Respir Crit Care Med 2007; 176(6): 532-55. Review.

D2(ii): Ramsdell J. Use of theophylline in the treatment of COPD. Chest 1995; 107(5 Suppl): 206S-209S. Review.

D2(iii):Fragoso CAV. Diagnosis and management of asthma in older adults. In: UpToDate, Post, TW (Ed), UpToDate, Waltham, MA, 2019 last accessed date 29 October 2019.

D2(iv): O'Mahony D, O'Sullivan D, Byrne S, O'Connor MN, Ryan C, Gallagher P. STOPP/START criteria for potentially inappropriate prescribing in older people: version 2. Age Ageing. 2015 Mar;44(2):213-8. doi: 10.1093/ageing/afu145. Epub 2014 Oct 16. Review.

D2(V): By the 2019 American Geriatrics Society Beers Criteria® Update Expert Panel. American Geriatrics Society 2019 Updated AGS Beers Criteria® for Potentially Inappropriate Medication Use in Older Adults. J Am Geriatr Soc. 2019 Apr;67(4):674-694. doi: 10.1111/jgs.15767.

#### D3. Systemic corticosteroids instead of inhaled corticosteroids for maintenance therapy in moderate-severe COPD (unnecessary exposure to long-term side-effects of systemic corticosteroids and effective inhaled therapies are available).

D3(i):Hess MW. The 2017 Global Initiative for Chronic Obstructive Lung Disease Report and Practice Implications for the Respiratory Therapist. Respir Care. 2017 Nov;62(11):1492-1500.

D3(ii): Wood-Baker R, Walters J, Walters EH. Systemic corticosteroids in chronic obstructive pulmonary disease: an overview of Cochrane systematic reviews. Respir Med 2007; 101(3): 371-7. Review.

D3(iii): O'Mahony D, O'Sullivan D, Byrne S, O'Connor MN, Ryan C, Gallagher P. STOPP/START criteria for potentially inappropriate prescribing in older people: version 2. Age Ageing. 2015 Mar;44(2):213-8. doi: 10.1093/ageing/afu145. Epub 2014 Oct 16. Review.

**Section E: Musculoskeletal System criteria and analgesic drugs.**

#### E1. Long-term use of NSAIDs (>3 months) in the presence of alternative treatment.  **Paracetamol should be used as first line for osteoarthritis pain or simple pain (headache, musculoskeletal, …etc.) (combinations of paracetamol with metamizole and low dose codeine/ tramadol might also be considered).* **Patients using NSAIDs should be followed up for possible side effects (nephropathy, hypertension, heart failure, cardiovascular events).* **Indomethacin should not be preferred if NSAID is indicated (more side effects on cenral nervous system and other systems than other NSAIDs in older adults).* **In cases where chronic NSAID use is required, PPI/ misoprostol should be used in addition to NSAIDs.*

E1(i): Nikles CJ, Yelland M, Del Mar C, Wilkinson D. The role of paracetamol in chronic pain: an evidence-based approach. Am J Ther 2005; 12(1): 80-91. Review.

E1(ii): Seed SM, Dunican KC, Lynch AM. Osteoarthritis: a review of treatment options. Geriatrics 2009; 64(10): 20-9. Review.

E1(iii): Jawad AS. Analgesics and osteoarthritis: are treatment guidelines reflected in clinical practice? Am J Ther 2005; 12(1): 98-103. Review.

E1(iv): By the 2019 American Geriatrics Society Beers Criteria® Update Expert Panel. American Geriatrics Society 2019 Updated AGS Beers Criteria® for Potentially Inappropriate Medication Use in Older Adults. J Am Geriatr Soc. 2019Apr;67(4):674-694.

E1(v): O'Mahony D, O'Sullivan D, Byrne S, O'Connor MN, Ryan C, Gallagher P. STOPP/START criteria for potentially inappropriate prescribing in older people: version 2. Age Ageing. 2015 Mar;44(2):213-8. doi: 10.1093/ageing/afu145. Epub 2014 Oct 16. Review.

#### E2. NSAIDs if eGFR< 50 ml/min/1.73m2 (risk of deterioration in renal function).

E2(i): Harirforoosh S, Jamali F. Renal adverse effects of non-steroidal anti-inflammatory drugs. Expert Opin Drug Saf 2009; 8(6): 669-81. Review.

E2(ii): Cheng HF, Harris RC. Renal effects of non-steroidal anti-inflammatory drugs and selective cyclooxygenase-2 inhibitors. Curr Pharm Des 2005; 11(14): 1795-804. Review.

E2 (iii): O'Mahony D, O'Sullivan D, Byrne S, O'Connor MN, Ryan C, Gallagher P. STOPP/START criteria for potentially inappropriate prescribing in older people: version 2. Age Ageing. 2015 Mar;44(2):213-8. doi: 10.1093/ageing/afu145. Epub 2014 Oct 16. Review.

#### E3. Systemic corticosteroids for osteoarthritis (risk of systemic corticosteroid side-effects).

E3(i): British National Formulary vol. 76, September 2018-March 2019: p 1058.

E3(ii): Recommendations for the medical management of osteoarthritis of the hip and knee: 2000 update. American College of Rheumatology Subcommittee on Osteoarthritis Guidelines. Arthritis Rheum 2000; 43(9): 1905-15.

E3(iii): O'Mahony D, O'Sullivan D, Byrne S, O'Connor MN, Ryan C, Gallagher P. STOPP/START criteria for potentially inappropriate prescribing in older people: version 2. Age Ageing. 2015 Mar;44(2):213-8. doi: 10.1093/ageing/afu145. Epub 2014 Oct 16. Review.

#### E4. Long-term corticosteroids (>3 months) as monotherapy for rheumatoid arthritis (risk of systemic corticosteroid side-effects).

E4(i): Onishi S, Iwmoto M, Minota S. Management of elderly-onset rheumatoid arthritis. J Clin Immunol 2010; 33(1): 1-7.

E4(ii): American College of Rheumatology Subcommittee on Rheumatoid Arthritis Guidelines. Guidelines for the management of rheumatoid arthritis: 2002 Update. Arthritis Rheum 2002; 46(2): 28-46.

E4(iii): Soubrier M, Mathieu S, Payet S, Dubost JJ, Ristori JM. Elderly-onset rheumatoid arthritis. Joint Bone Spine 2010; 77(4): 290-6. Review.

E4(iv): O'Mahony D, O'Sullivan D, Byrne S, O'Connor MN, Ryan C, Gallagher P. STOPP/START criteria for potentially inappropriate prescribing in older people: version 2. Age Ageing. 2015 Mar;44(2):213-8. doi: 10.1093/ageing/afu145. Epub 2014 Oct 16. Review.

#### E4(v):Burmester GR, Pope JE. Novel treatment strategies in rheumatoid arthritis.Lancet. 2017 Jun 10;389(10086):2338-2348.

#### E4( vi):Roubille C, Richer V, Starnino T, McCourt C, McFarlane A, Fleming P, Siu S, Kraft J, Lynde C, Pope J, Gulliver W, Keeling S, Dutz J, Bessette L, Bissonnette R, Haraoui B. The effects of tumour necrosis factor inhibitors, methotrexate, non-steroidal anti-inflammatory drugs and corticosteroids on cardiovascular events in rheumatoid arthritis, psoriasis and psoriatic arthritis: a systematic review and meta-analysis. Ann Rheum Dis. 2015 Mar;74(3):480-9.

#### E5. Long-term NSAID or colchicine for chronic treatment of gout where there is no contraindication to a xanthine-oxidase inhibitor (e.g. allopurinol, febuxostat) (xanthine-oxidase inhibitors are first choice prophylactic drugs in gout).

E5(i): De Leonardis F, Govoni M, Colina M, Bruschi M, Trotta F. Elderly-onset gout: a review. Rheumatol Int 2007; 28(1): 1-6. Review.

E5(ii): Hoskison KT, Wortmann RL. Management of gout in older adults: barriers to optimal control. Drugs Aging 2007; 24(1): 21-36. Review.

E5 (iii): O'Mahony D, O'Sullivan D, Byrne S, O'Connor MN, Ryan C, Gallagher P. STOPP/START criteria for potentially inappropriate prescribing in older people: version 2. Age Ageing. 2015 Mar;44(2):213-8. doi: 10.1093/ageing/afu145. Epub 2014 Oct 16. Review.

E5(iv): British National Formulary vol. 76, September 2018-March 2019: p 1085-87.

#### E6. Colchicine if eGFR <10 ml/min/1.73m2 (risk of colchicine toxicity).

E6(i): Hoskison KT, Wortmann RL. Management of gout in older adults: barriers to optimal control. Drugs Aging 2007; 24(1): 21-36. Review.

E6(ii): Hanlon JT, Aspinall SL, Semla TP, Weisbord SD, Fried LF, Good CB, Fine MJ, Stone RA, Pugh MJ, Rossi MI, Handler SM. Consensus guidelines for oral dosing of primarily renally cleared medications in older adults. J Am Geriatr Soc 2009; 57(2):335-40. Erratum in: J Am Geriatr Soc 2009; 57(11): 2179. Dosage error in article text.

E6(iii): O'Mahony D, O'Sullivan D, Byrne S, O'Connor MN, Ryan C, Gallagher P. STOPP/START criteria for potentially inappropriate prescribing in older people: version 2. Age Ageing. 2015 Mar;44(2):213-8. doi: 10.1093/ageing/afu145. Epub 2014 Oct 16. Review.

E6(iv): BritishNational Formulary vol. 76, September 2018-March 2019: p 1085-86.

#### E7. Methotrexate if eGFR <30 ml/min/1.73m2.

E7(i): Seyffart's Directory of Drug Dosage in Kidney Disease; (1st ed., 2011) by GünterSeyffart Publisher: Dustri‐Verlag Dr. Karl Feistle GmbH & Co. KG, Munich‐Orlando; pp: 476-477.

E7(ii): Methotrexate : Drug information, Lexicomp Online. Last accessed date 22 October 2019.

#### E8. Meperidine in the treatment of pain (increased neurotoxicity, delirium risk, safer alternatives are available. Increased risk in especially patients with renal failure).

E8(i): By the 2019 American Geriatrics Society Beers Criteria® Update Expert Panel. American Geriatrics Society 2019 Updated AGS Beers Criteria® for Potentially Inappropriate Medication Use in Older Adults. J Am Geriatr Soc. 2019Apr;67(4):674-694.

E8(ii):Meperidine (pethidine) : Drug information, Lexicomp Online. Last accessed date 22 October 2019.

#### E9. Extended-release tramadol if eGFR <30 ml/min/1.73m2.

#### **Dose reduction should be performed for rapid release tramadol.*

E9(i): By the 2019 American Geriatrics Society Beers Criteria® Update Expert Panel. American Geriatrics Society 2019 Updated AGS Beers Criteria® for Potentially Inappropriate Medication Use in Older Adults. J Am Geriatr Soc. 2019Apr;67(4):674-694.

E9(ii):Tramadol: Drug information, Lexicomp Online. Last accessed date 22 October 2019.

#### E10. Regular opioids without concomitant laxative (risk of severe constipation).

E10(i): Forman WB. Opioid analgesic drugs in the elderly. Clin Geriatr Med 1996; 12(3): 489-500. Review.

E10(ii): Kalso E, Edwards JE, Moore RA, McQuay HJ. Opioids in chronic non-cancer pain: systematic review of efficacy and safety. Pain 2004; 112(3): 372-80.

E10(iii):O'Mahony D, O'Sullivan D, Byrne S, O'Connor MN, Ryan C, Gallagher P. STOPP/START criteria for potentially inappropriate prescribing in older people: version 2. Age Ageing. 2015 Mar;44(2):213-8. doi: 10.1093/ageing/afu145. Epub 2014 Oct 16. Review.

E10(iv):Galicia-Castillo MC, Weiner DK. Treatment of persistent pain in older adults. In: UpToDate, Post, TW (Ed), UpToDate, Waltham, MA, 2019 last accessed date 23 October 2019.

#### E11. Systemic skeletal muscle relaxant agents (thiocolchicoside, tizanidine, chlorzoxazone, carisoprodol, chlorfenese carbamate, cyclobenzaprine, metaxalone, methocarbamol and orphenadrine) for musculoskeletal pain (sedation, dizziness, dry mouth, constipation, cognitive impairment). **The risk of hypotension with tizanidine is also prominent.*

E11(i): By the 2019 American Geriatrics Society Beers Criteria® Update Expert Panel. American Geriatrics Society 2019 Updated AGS Beers Criteria® for Potentially Inappropriate Medication Use in Older Adults. J Am Geriatr Soc. 2019Apr;67(4):674-694.

E11(ii): A.R Umarkar, S.R Bavaskar and P.N.Yewale.Thiocolchicoside as muscle relaxant: a review; International Journal of Pharmacy and Biological Sciences (eISSN: 2230-7605).Volume 1, Issue 3, JULY-SEPT, 2011;364-371.Available at: <https://www.ijpbs.com/ijpbsadmin/upload/ijpbs_50c8471a463c9.pdf>last accessed date 29 October 2019.

E11(iii): Thiocolchicoside: review of adverse effects. Prescrire Int. 2016; Feb;25(168):41-3

E11 (iv): Tizanidine: Drug information, Lexicomp Online. Last accessed date 22 October 2019.

#### E12. Initiation of osteoporosis treatment without excluding osteomalacia diagnosis.

E12(i): Cosman F, de Beur SJ, LeBoff MS, Lewiecki EM, Tanner B, Randall S, Lindsay R; National Osteoporosis Foundation. Clinician's Guide to Prevention and Treatment of Osteoporosis. Osteoporos Int. 2014 Oct;25(10):2359-81.

#### E13. Conventional vitamin D at high intermittent doses (300,000 IU) as ‘maintenance’ vitamin D therapy (increased fall risk, no additional benefit on the musculoskeletal system). *[Unfavourable](https://www.seslisozluk.net/unfavourable-nedir-ne-demek/) *effects of high dose conventional vitamin D have been shown in ''maintenance'' vitamin D therapy.*

#### **Such adverse effects have not been shown in“replacement” vitamin Dtherapy. However, due to the “start low- go slow” principle generally valid in drug use in older adults, gradual vitamin D treatment may also be appropriate in “replacement“ therapy.*

E13(i): Sanders KM, Stuart AL, Williamson EJ, Simpson JA, Kotowicz MA, Young D, Nicholson GC. Annual high-dose oral vitamin D and falls and fractures in older women: a randomized controlled trial. JAMA. 2010 May 12;303(18):1815-22.

E13(ii): Bischoff-Ferrari HA, Dawson-Hughes B, Orav EJ, Staehelin HB, Meyer OW, Theiler R, Dick W, Willett WC, Egli A. Monthly High-Dose Vitamin D Treatment for the Prevention of Functional Decline: A Randomized Clinical Trial. JAMA Intern Med. 2016 Feb;176(2):175-83.

E13(iii): Smith H, Anderson F, Raphael H, Maslin P, Crozier S, Cooper C. Effect of annual intramuscular vitamin D on fracture risk in elderly men and women—a population-based, randomized, double-blind, placebo-controlled trial. Rheumatology (Oxford). 2007 Dec;46(12):1852-7.

E13(iv): Heikinheimo RJ, Inkovaara JA, Harju EJ, Haavisto MV, Kaarela RH, Kataja JM, Kokko AM, Kolho LA, Rajala SA. Annual injection of vitamin D and fractures of aged bones. Calcif Tissue Int. 1992 Aug;51(2):105-10.

E13(v):Cosman F, de Beur SJ, LeBoff MS, Lewiecki EM, Tanner B, Randall S, Lindsay R; National Osteoporosis Foundation. Clinician's Guide to Prevention and Treatment of Osteoporosis. Osteoporos Int. 2014 Oct;25(10):2359-81. doi: 10.1007/s00198-014-2794-2. Epub 2014 Aug 15. Erratum in: Osteoporos Int. 2015 Jul;26(7):2045-7.

E13(vi): Shah S, Chiang C, Sikaris K, Lu Z, Bui M, Zebaze R, Seeman E. Serum 25-Hydroxyvitamin D Insufficiency in Search of a Bone Disease. J Clin Endocrinol Metab. 2017 Jul 1;102(7):2321-2328.

#### E14. Active vitamin D (1-25 (OH)2 cholecalciferol) (calcitriol) or conventional  vitamin D (25 (OH) cholecalciferol)  in those with hyperphosphatemia and/ or hypercalcemia.

## E14(i): Seyffart's Directory of Drug Dosage in Kidney Disease; (1st ed., 2011) by GünterSeyffart Publisher: Dustri‐Verlag Dr. Karl Feistle GmbH & Co. KG, Munich‐Orlando; pp: 476-477.

E14(ii): Kidney Disease: Improving Global Outcomes (KDIGO) CKD-MBD Update Work Group. KDIGO 2017 Clinical Practice Guideline Update for the Diagnosis, Evaluation, Prevention, and Treatment of Chronic Kidney Disease–Mineral and Bone Disorder (CKD-MBD). Kidney Int Suppl. 2017;7:1–59.

#### E15. Oral bisphosphonates in patients with a history ofupper gastrointestinal disease (i.e. dysphagia, esophagitis, peptic ulcer disease, upper gastrointestinal bleeding or gastroesophageal reflux that cannot be controlled by treatment) and/or those who cannot stand or sit in up right position (risk of relapse/exacerbation of esophagitis, esophageal ulcer, esophageal stricture). **Oral bisphosphonates can be used with caution in case of well controlled* gastroesophageal reflux.

E15(i): Pazianas M, Abrahamsen B. Safety of bisphosphonates. Bone 2011; 49(1): 103-10. Review.

E15(ii): Civitelli R, Napoli N, Armamento-Villareal R. Use of intravenous bisphosphonates in osteoporosis. Curr Osteoporos Rep 2007;5(1): 8-13.

E15(iii): Gaudio A, Morabito N. Pharmacological management of severe postmenopausal osteoporosis. Drugs Aging 2005; 22(5): 405-17. Review.

E15(iv):Lewiecki EM. Bisphosphonates for the treatment of osteoporosis: insights forclinicians. Ther Adv Chronic Dis. 2010 May;1(3):115-28.

E15(v): Kennel KA, Drake MT. Adverse effects of bisphosphonates: implications forosteoporosis management. Mayo Clin Proc. 2009 Jul;84(7):632-7; quiz 638.

E15 (vi): O'Mahony D, O'Sullivan D, Byrne S, O'Connor MN, Ryan C, Gallagher P. STOPP/START criteria for potentially inappropriate prescribing in older people: version 2. Age Ageing. 2015 Mar;44(2):213-8. doi: 10.1093/ageing/afu145. Epub 2014 Oct 16. Review.

E15 (vii):Alendronate: Drug information, Lexicomp Online. Last accessed date 29 October 2019.

E15 (viii):Risedronate: Drug information, Lexicomp Online. Last accessed date 29 October 2019.

E15(ix):Ibandronate: Drug information, Lexicomp Online. Last accessed date 29 October 2019.

E15(x):RosenHN**.**The use of bisphosphonates in postmenopausal women with osteoporosis***.*** In: UpToDate, Post, TW (Ed), UpToDate, Waltham, MA, 2019 last accessed date 23 October 2019.

#### E16. Bisphosphonates if eGFR<30 ml/min/1.73m2 (increased risk of acute renal failure). **The threshold eGFR for alendronat and zoledronate use is higher (<35 ml/min/1.73m2).*

E16(i): Cosman F, de Beur SJ, LeBoff MS, Lewiecki EM, Tanner B, Randall S, Lindsay R; National Osteoporosis Foundation. Clinician's Guide to Prevention and Treatment of Osteoporosis. Osteoporos Int. 2014 Oct;25(10):2359-81.

E16(ii):Alendronate: Drug information, Lexicomp Online. Last accessed date 29 October 2019.

E16(iii):Risedronate: Drug information, Lexicomp Online. Last accessed date 29 October 2019.

E16(iv):Ibandronate: Drug information, Lexicomp Online. Last accessed date 29 October 2019.

E16(v):RosenHN**.**The use of bisphosphonates in postmenopausal women with osteoporosis***.*** In: UpToDate, Post, TW (Ed), UpToDate, Waltham, MA, 2019 last accessed date 23 October 2019.

#### E17. Zoledronate, denosumab or teriparatide without monitoring serum calcium level and assuring adequate calcium/ vitamin D intake prior to the treatment.

E17(i):Denosumab: Drug information, Lexicomp Online. Last accessed date 22 October 2019.

E17(ii):Zoledronic acid: Drug information, Lexicomp Online. Last accessed date 22 October 2019.

E17(iii): Teriparatide (recombinant human parathyroid hormone [1-34]) : Drug information, Lexicomp Online. Last accessed date 22 October 2019.

**Section F: Urogenital System criteria.**

#### F1. Bladder anticholinergic drugs if PVR>150 mlin men with symptoms of LUTS due to benign prostatic hyperplasia. **In older men, the symptoms of LUTS should be questioned before prescribing bladder anticholinergic drugs for overactive bladder therapy and PVR measurement should be performed in all cases.*

F1(i): McVary KT, Roehrborn CG, Avins AL, Barry MJ, Bruskewitz RC, Donnell RF, FosterHE Jr, Gonzalez CM, Kaplan SA, Penson DF, Ulchaker JC, Wei JT. Update on AUAguideline on the management of benign prostatic hyperplasia. J Urol. 2011May;185(5):1793-803.

F1(ii): O'Mahony D, O'Sullivan D, Byrne S, O'Connor MN, Ryan C, Gallagher P. STOPP/START criteria for potentially inappropriate prescribing in older people: version 2. Age Ageing. 2015 Mar;44(2):213-8. doi: 10.1093/ageing/afu145. Epub 2014 Oct 16. Review.

F1(iii): S. Gravas (Chair), J.N. Cornu, M.J. Drake, M. Gacci, C. Gratzke, T.R.W. Herrmann, S. Madersbacher, C. Mamoulakis, K.A.O. Tikkinen Guidelines Associates: M. Karavitakis, I. Kyriazis, S. Malde, V. Sakkalis, R. Umbach. EAU Guidelines on Management of Non-Neurogenic Male Lower Urinary Tract Symptoms (LUTS), incl. Benign Prostatic Obstruction (BPO). European Association of Urology 2018. Page 25.

#### F2. Bladder anticholinergics in patients with chronic narrow-angle glaucoma. **The history of glaucoma should be questioned before starting bladder anticholinergics.* **Bladder anticholinergics are not contraindicated if narrow-angle glaucoma is treated with laser iridotomy.*

#### **Bladder anticholinergics are not contraindicated in open-angle glaucoma.*

F2(i): O'Mahony D, O'Sullivan D, Byrne S, O'Connor MN, Ryan C, Gallagher P. STOPP/START criteria for potentially inappropriate prescribing in older people: version 2. Age Ageing. 2015 Mar;44(2):213-8. doi: 10.1093/ageing/afu145. Epub 2014 Oct 16. Review.

F2(ii). Gani J, Perlis N, Radomski SB. Urologic medications and ophthalmologic side effects: a review. Can Urol Assoc J. 2012 Feb;6(1):53-8. doi: 10.5489/cuaj.11037.

F2(iii): Kato K, Yoshida K, Suzuki K, Murase T, Gotoh M. Managing patients with an overactive bladder and glaucoma: a questionnaire survey of Japanese urologists on the use of anticholinergics. BJU Int. 2005 Jan;95(1):98-101.

F2(iv): Oxybutynin: Drug information, Lexicomp Online. Last accessed date 29 October 2019.

F2(v):Darifenacin: Drug information, Lexicomp Online. Last accessed date 29 October 2019.

F2(vi):Tolterodine: Drug information, Lexicomp Online. Last accessed date 29 October 2019.

F2(vii):Trospium: Drug information, Lexicomp Online. Last accessed date 29 October 2019.

F2(viii):Fesoterodine: Drug information, Lexicomp Online. Last accessed date 29 October 2019.

F2(ix):Solifenacin: Drug information, Lexicomp Online. Last accessed date 29 October 2019.

#### F3. Bladder anticholinergics without PVR determination with concurrent prostatic hyperplasia (risk of obstruction) or diabetes mellitus with complications (risk for neurogenic bladder) or frailty (risk for reduced contractility with detrusor hyperactivity). [Risk of urinary retention and post-renal renal failure].

F3(i): Taylor JA 3rd, Kuchel GA. Detrusor underactivity: Clinical features and pathogenesis of an underdiagnosed geriatric condition. J Am Geriatr Soc. 2006 Dec;54(12):1920-32. Review.

F3(ii): S. Gravas (Chair), J.N. Cornu, M.J. Drake, M. Gacci, C. Gratzke, T.R.W. Herrmann, S. Madersbacher, C. Mamoulakis, K.A.O. Tikkinen Guidelines Associates: M. Karavitakis, I. Kyriazis, S. Malde, V. Sakkalis, R. Umbach. EAU Guidelines on Management of Non-Neurogenic Male Lower Urinary Tract Symptoms (LUTS), incl. Benign Prostatic Obstruction (BPO). European Association of Urology 2018. Page 25.

F3(iii): Golbidi S, Laher I. Bladder dysfunction in diabetes mellitus. Front Pharmacol.2010 Nov 16;1:136. doi: 10.3389/fphar.2010.00136. eCollection 2010.

F3(iv): O'Mahony D, O'Sullivan D, Byrne S, O'Connor MN, Ryan C, Gallagher P. STOPP/START criteria for potentially inappropriate prescribing in older people: version 2. Age Ageing. 2015 Mar;44(2):213-8. doi: 10.1093/ageing/afu145. Epub 2014 Oct 16. Review.

#### F4. Phosphodiesterase type-5 inhibitors (e.g. sildenafil, tadalafil, vardenafil) in patients with blood pressure <90/50 mmHg or >170/100 mmHg/unstable angina/angina during sexual intercourse/NYHA class IV heart failure/receiving nitrate for angina/ receiving alpha-1 blocker/ recent myocardial infarction (< three months)/ recent stroke (< six months).

F4(i): Kloner RA, Hutter AM, Emmick JT, Mitchell MI, Denne J, Jackson G. Time course of the interaction between tadalafil and nitrates. J Am Coll Cardiol 2003; 42 (10): 1855-60.

F4(ii): O'Mahony D, O'Sullivan D, Byrne S, O'Connor MN, Ryan C, Gallagher P. STOPP/START criteria for potentially inappropriate prescribing in older people: version 2. Age Ageing. 2015 Mar;44(2):213-8. doi: 10.1093/ageing/afu145. Epub 2014 Oct 16. Review.

F4(iii): S. Gravas (Chair), J.N. Cornu, M.J. Drake, M. Gacci, C. Gratzke, T.R.W. Herrmann, S. Madersbacher, C. Mamoulakis, K.A.O. Tikkinen Guidelines Associates: M. Karavitakis, I. Kyriazis, S. Malde, V. Sakkalis, R. Umbach. EAU Guidelines on Management of Non-Neurogenic Male Lower Urinary Tract Symptoms (LUTS), incl. Benign Prostatic Obstruction (BPO). European Association of Urology 2018. Page 21.

F4(iv): K. Hatzimouratidis (Chair), F. Giuliano, I. Moncada, A. Muneer, A. Salonia (Vice-chair), P. Verze Guideline Associates: A. Parnham, E.C. Serefoglu. EAU Guidelines on Erectile Dysfunction, Premature Ejaculation, Penile Curvature and Priapism. European Association of Urology 2018. Page 20-21.

#### F5. Non-uroselective alpha-1 blockers (e.g. doxazosin, terazosin) for the treatment of LUTS symptoms related to benign prostatic hyperplasia in patients with orthostatic hypotension. **The risk of orthostatic hypotension is lowest with uroselective agent silodosin and is suggested similar to placebo.* **Older adults who are treated with non-uroselective alpha-1 blockers should be informed about the risk of orthostatic hypotension.*

F5(i): S. Gravas (Chair), J.N. Cornu, M.J. Drake, M. Gacci, C. Gratzke, T.R.W. Herrmann, S. Madersbacher, C. Mamoulakis, K.A.O. Tikkinen Guidelines Associates: M. Karavitakis, I. Kyriazis, S. Malde, V. Sakkalis, R. Umbach. EAU Guidelines on Management of Non-Neurogenic Male Lower Urinary Tract Symptoms (LUTS), incl. Benign Prostatic Obstruction (BPO). European Association of Urology 2018. Page 17.

F5(ii):Chapple CR, Montorsi F, Tammela TL, Wirth M, Koldewijn E, Fernández Fernández E; European Silodosin Study Group. Silodosin therapy for lower urinary tractsymptoms in men with suspected benign prostatic hyperplasia: results of aninternational, randomized, double-blind, placebo- and active-controlled clinical trial performed in Europe. Eur Urol. 2011 Mar;59(3):342-52.

F5(iii): Welk B, McArthur E, Fraser LA, Hayward J, Dixon S, Hwang YJ, Ordon M. The riskof fall and fracture with the initiation of a prostate-selective α antagonist: a population based cohort study. BMJ. 2015 Oct 26;351:h5398.

F5(iv): O'Mahony D, O'Sullivan D, Byrne S, O'Connor MN, Ryan C, Gallagher P. STOPP/START criteria for potentially inappropriate prescribing in older people: version 2. Age Ageing. 2015 Mar;44(2):213-8. doi: 10.1093/ageing/afu145. Epub 2014 Oct 16. Review.

F5(v): Cho HJ, Yoo TK. Silodosin for the treatment of clinical benign prostatic hyperplasia: safety, efficacy, and patient acceptability. Res Rep Urol. 2014 Sep 26;6:113-9. doi: 10.2147/RRU.S41618. eCollection 2014. Review.

F5(vi): S. Gravas (Chair), T. Bach, M. Drake, M. Gacci, C. Gratzke, T.R.W. Herrmann, S. Madersbacher, C. Mamoulakis, K.A.O. Tikkinen Guidelines Associates: M. Karavitakis, S. Malde, V. Sakkalis, R. Umbach. EAU Guidelines on Management of Non-Neurogenic Male Lower Urinary Tract Symptoms (LUTS), incl. Benign Prostatic Obstruction (BPO). European Association of Urology 2017; Page 14-15.

F5(vii):Chatziralli IP, Sergentanis TN. Risk factors for intraoperative floppy iris syndrome: a meta-analysis. Ophthalmology. 2011 Apr;118(4):730-5.

F5(viii): Cantrell MA, Bream-Rouwenhorst HR, Steffensmeier A, Hemerson P, Rogers M,Stamper B. Intraoperative floppy iris syndrome associated with alpha1-adrenergic receptor antagonists. Ann Pharmacother. 2008 Apr;42(4):558-63. doi:10.1345/aph.1K679. Epub 2008 Mar 25. Review.

F5(ix):Chang DF, Campbell JR. Intraoperative floppy iris syndrome associated with tamsulosin. J Cataract Refract Surg. 2005 Apr;31(4):664-73.

F5(x):Chang DF, Osher RH, Wang L, Koch DD. Prospective multicenter evaluation ofcataract surgery in patients taking tamsulosin (Flomax). Ophthalmology. 2007May;114(5):957-64.

F5(xi):Bell CM, Hatch WV, Fischer HD, Cernat G, Paterson JM, Gruneir A, Gill SS,Bronskill SE, Anderson GM, Rochon PA. Association between tamsulosin and serious ophthalmic adverse events in older men following cataract surgery. JAMA. 2009 May20;301(19):1991-6.

F5(xii):Gani J, Perlis N, Radomski SB. Urologic medications and ophthalmologic side effects: a review. Can Urol Assoc J. 2012 Feb;6(1):53-8.

#### F6. Antibiotic use in asymptomatic bacteriuria except during urological interventions that may damage the mucosa. **Asymptomatic bacteriuria should be screened and treated prior to urologic interventions that may damage the mucosa.* **Screening and/ or treatment of asymptomatic bacteriuria in the following patient groups are not recommended:*

#### *Patients without risk factors* *Patients with well-regulated diabetes* *Patients living in nursing homes* *Patients with lower urinary tract dysfunction/ reconstruction* *Patients with recurrent urinary tract infections* *Before arthroplasty operations* *Patients with urinary catheter*

F6(i): Lindsay E. Nicolle, Suzanne Bradley, Richard Colgan, James C. Rice, Anthony Schaeffer, Thomas M. Hooton, Infectious Diseases Society of America Guidelines for the Diagnosis and Treatment of Asymptomatic Bacteriuria in Adults, Clinical Infectious Diseases, Volume 40, Issue 5, 1 March 2005, Pages 643–654.

F6(ii): G. Bonkat (Co-chair), R. Pickard (Co-chair), R. Bartoletti, T. Cai, F. Bruyère, S.E. Geerlings, B. Köves, F. Wagenlehner Guidelines Associates: A. Pilatz, B. Pradere, R. Veeratterapillay. EAU Guidelines onUrological Infections. European Association of Urology 2018.

#### F7. Nitrofurantoin if eGFR <30 ml/min/1.73m2. **Long-term use of nitrofurantoin for suppression treatment of urinary infections is not appropriate for older adults (risk of irreversible pulmonary fibrosis, liver toxicity and peripheral neuropathy with prolonged use).*

F7(i): By the 2019 American Geriatrics Society Beers Criteria® Update Expert Panel. American Geriatrics Society 2019 Updated AGS Beers Criteria® for Potentially Inappropriate Medication Use in Older Adults. J Am Geriatr Soc. 2019Apr;67(4):674-694.

F7(ii) G. Bonkat (Co-chair), R. Pickard (Co-chair), R. Bartoletti, T. Cai, F. Bruyère, S.E. Geerlings, B. Köves, F. Wagenlehner Guidelines Associates: A. Pilatz, B. Pradere, R. Veeratterapillay. EAU Guidelines onUrological Infections. European Association of Urology 2018.

**Section J: Endocrine System criteria.**

#### G1. Intensive glycemic control (HbA1C< 7%) in patients with limited life expectancy (<5 years) or history of falls or cognitive impairment.

G1(i): Onder G, Landi F, Fusco D, Corsonello A, Tosato M, Battaglia M, Mastropaolo S, Settanni S, Antocicco M, Lattanzio F. Recommendations to prescribe in complex older adults: results of the CRIteria to assess appropriate Medication use among Elderly complex patients (CRIME) project. Drugs Aging. 2014 Jan;31(1):33-45.

G1(ii): Medha M. Treatment of type 2 diabetes mellitus in the older patient. In: UpToDate, Post, TW (Ed), UpToDate, Waltham, MA, 2019 last accessed date 23 October 2019

#### G2. Metformin in malnourished/frail patients (due to GIS side effects and loss of appetite). **In cases with malnutrition risk, the use of metformin should be decided by taking the profit and loss balance into consideration.*

G2(i): Haas L. Management of diabetes mellitus medications in the nursing home. *Drugs Aging*. 2005;22:209–218.

G2(ii): Onder G, Landi F, Fusco D, Corsonello A, Tosato M, Battaglia M, Mastropaolo S, Settanni S, Antocicco M, Lattanzio F. Recommendations to prescribe in complex older adults: results of the CRIteria to assess appropriate Medication use among Elderly complex patients (CRIME) project. Drugs Aging. 2014 Jan;31(1):33-45.

G3(iii): Bahat G, Erdogan T, Karan MA. Need for increased awareness for avoiding metformin treatment in malnourished older adults with diabetes mellitus. Clinical Nutrition, 2019.

#### G3.Metformin if eGFR <30 ml/min/ 1.73 m2 (risk of lactic acidosis). **Metformin dose should be reduced by 50% in patients with GFR: 30-45 ml/min/1.73m2.* **Metformin use is not appropriate also in conditions that increase the risk of lactic acidosis (heart failure, liver failure, shock or permanent hemodynamic instability, COPD, hypoxia).*

G3(i):Metformin: Drug information, Lexicomp Online. Last accessed date 22 October 2019.

G3(ii): Germino FW. Non-insulin treatment of type 2 diabetes mellitus in geriatric patients: a review. Clin Ther 2011; 33(12): 1868-82.

G3(iii): Lalau JD.Lactic acidosis induced by metformin: Incidence, management and prevention. Drug Safety 2010; 33(9): 727-40.

G3(iv): O'Mahony D, O'Sullivan D, Byrne S, O'Connor MN, Ryan C, Gallagher P. STOPP/START criteria for potentially inappropriate prescribing in older people: version 2. Age Ageing. 2015 Mar;44(2):213-8.

#### G4. Sulphonylureas with long duration of action (i.e. glibenclamide or chlorpropamide) in patients with type 2 diabetes mellitus (risk of prolonged hypoglycemia).

G4(i): David K M. Sulfonylureas and meglitinides in the treatment of diabetes mellitus. In: UpToDate, Post, TW (Ed), UpToDate, Waltham, MA, 2019 last accessed date 29 October 2019

G4(ii): Graal MB, Wolffenbuttel BH. The use of sulphonylureas in the elderly. Drugs Aging 1999; 15(6): 471-81.

G4(iii): Langtry HD, Balfour JA. Glimepiride. A review of its use in the management of type 2 diabetes mellitus. Drugs 1998; 55(4): 563-84.

G4(iv): O'Mahony D, O'Sullivan D, Byrne S, O'Connor MN, Ryan C, Gallagher P. STOPP/START criteria for potentially inappropriate prescribing in older people: version 2. Age Ageing. 2015 Mar;44(2):213-8.

#### G5. Thiazolidinediones (i.e. rosiglitazone, pioglitazone) in patients with heart failure/ a history of fracture/ increased fracture risk/ a history of bladder cancer or on insulin treatment (risk of exacerbation of heart failure, increase in the risk of fracture and bladder cancer).

G5(i): David K M.Thiazolidinediones in the treatment of diabetes mellitus. In: UpToDate, Post, TW (Ed), UpToDate, Waltham, MA, 2019 last accessed date 29 October 2019

G5(ii): Rosiglitazone: Drug information, Lexicomp Online. Last accessed date 04 November 2019.

G5(iii): Germino FW. Noninsulin treatment of type 2 diabetes mellitus in geriatric patients: a review. Clin Ther 2011; 33(12): 1868-82.

G5(iv): Lago RM, Singh PP, Nesto RW. Congestive heart failure and cardiovascular death in patients with prediabetes and type 2 diabetes given thiazolidinediones: a meta-analysis of randomised clinical trials. Lancet 2007; 370(9593): 1129-36.

G5(v): O'Mahony D, O'Sullivan D, Byrne S, O'Connor MN, Ryan C, Gallagher P. STOPP/START criteria for potentially inappropriate prescribing in older people: version 2. Age Ageing. 2015 Mar;44(2):213-8.

#### G6. Saxagliptin in patients with heart failure.

G6(i): LeRoith D, Biessels GJ, Braithwaite SS, Casanueva FF, Draznin B, Halter JB, Hirsch IB, McDonnell ME, Molitch ME, Murad MH, Sinclair AJ. Treatment of Diabetes in Older Adults: An Endocrine Society* Clinical Practice Guideline. J Clin Endocrinol Metab. 2019 May 1;104(5):1520-1574.

G6(ii): Scirica BM, Braunwald E, Raz I, Cavender MA, Morrow DA, Jarolim P, Udell JA, Mosenzon O, Im K, Umez-Eronini AA, Pollack PS, Hirshberg B, Frederich R, Lewis BS, McGuire DK, Davidson J, Steg PG, Bhatt DL; SAVOR-TIMI 53 Steering Committee and Investigators. Heart Failure, Saxagliptin, and Diabetes Mellitus: Observations from the SAVOR-TIMI 53 Randomized Trial. Circulation. 2015 Oct 13;132(15):e198.

#### G7. Canagliflozin in patients with fracture/ recurrent urinary tract infection/ genitourinary infection/ severe peripheric artery disease/ lower extremity amputation due to diabetes. **SGLT-2 inhibitors may cause dehydration, urgency incontinence and diabetic ketoacidosis; they should be used with caution in older adults.*

G7(i): U.S. Food and Drug Administration. FDA confirms increased risk of leg and foot amputations with the diabetes medicine canagliflozin (Invokana, Invokamet, Invokamet XR). Available at: [www.fda.gov/downloads/Drugs/DrugSafety/UCM558427.pdf](http://www.fda.gov/downloads/Drugs/DrugSafety/UCM558427.pdf). (last accesed date 30 October 2019)

G7(ii): Sinclair AJ, Bode B, Harris S, Vijapurkar U, Shaw W, Desai M, Meininger G. Efficacy and Safety of Canagliflozin in Individuals Aged 75 and Older with Type 2 Diabetes Mellitus: A Pooled Analysis. J Am Geriatr Soc. 2016 Mar;64(3):543-52.

#### G7(iii): Watts NB, Bilezikian JP, Usiskin K, Edwards R, Desai M, Law G, Meininger G. Effects of Canagliflozin on Fracture Risk in Patients With Type 2 Diabetes Mellitus. J Clin Endocrinol Metab. 2016 Jan;101(1):157-66.

G7(iv): Anthony D. Sodium-glucose co-transporter 2 inhibitors for the treatment of hyperglycemia in type 2 diabetes mellitus. In: UpToDate, Post, TW (Ed), UpToDate, Waltham, MA, 2019 last accessed date 29 October 2019

G7(v): Lupsa BC, Inzucchi SE. Use of SGLT2 inhibitors in type 2 diabetes: weighing the risks and benefits. Diabetologia. 2018 Oct;61(10):2118-2125.

#### G8. SGLT-2 inhibitors for glycemic regulation if eGFR<45 mL/min/1.73 m.

**Canagliflozin,*empagliflozin, and ertugliflozin cannot be used if eGFR <45mL/min/1.73m2, primarily because of a decrease in efficacy.

*Dapagliflozin cannot be used if eGFR <60 mL/ min /1.73 m2, *primarily because of a decrease in efficacy.*

**For the treatment of nephropathy (urine albumin excretion >300 mg/day), however, SGLT2 inhibitors have established renal benefits in patients with eGFR 30-60 mL/min/1.73m2.*

G8(i): LeRoith D, Biessels GJ, Braithwaite SS, Casanueva FF, Draznin B, Halter JB, Hirsch IB, McDonnell ME, Molitch ME, Murad MH, Sinclair AJ. Treatment of Diabetes in Older Adults: An Endocrine Society* Clinical Practice Guideline. J Clin Endocrinol Metab. 2019 May 1;104(5):1520-1574.

G8(ii): George L B. Treatment of diabetic kidney disease. In: UpToDate, Post, TW (Ed), UpToDate, Waltham, MA, 2019 last accessed date 29 October 2019

#### G9. Androgens (male sex hormones) in the presence of low serum testosterone levels with no signs and symptoms associated with androgen deficiency. **Hypogonadism is diagnosed in the presence of persistent low serum testosterone levels with signs and symptoms of androgen insufficiency*.

G9(i): O'Mahony D, O'Sullivan D, Byrne S, O'Connor MN, Ryan C, Gallagher P. STOPP/START criteria for potentially inappropriate prescribing in older people: version 2. Age Ageing. 2015 Mar;44(2):213-8.

G9(ii): Bhasin S, Brito JP, Cunningham GR, Hayes FJ, Hodis HN, Matsumoto AM, Snyder PJ, Swerdloff RS, Wu FC, Yialamas MA. Testosterone Therapy in Men With Hypogonadism: An Endocrine Society Clinical Practice Guideline. J Clin Endocrinol Metab. 2018 May 1;103(5):1715-1744.

G9(iii): G.R. Dohle, S. Arver, C. Bettocchi, T.H. Jones, S. Kliesch. EAU Guidelines on Male Hypogonadism. In: European Association of Urology 2018, last accessed date 29 October 2019.

#### G10.  Systemic estrogens with a history of breast cancer or venous thromboembolism (increased risk of recurrence). *Vaginal estrogen can be given after non-hormone treatments in the treatment of urogenital symptoms such as atrophic vaginitis, *considering the benefit and harm balance in patients with a history of breast cancer or venous thromboembolism.*

G10(i):American College of Obstetricians and Gynecologists’ Committee on Gynecologic Practice, Farrell R. ACOG Committee Opinion No. 659: The Use of Vaginal Estrogen in Women With a History of Estrogen-Dependent Breast Cancer. Obstet Gynecol. 2016 Mar;127(3):e93-6.

G10(ii): The NAMS 2017 Hormone Therapy Position Statement Advisory Panel. The 2017 hormone therapy position statement of The North American Menopause Society. Menopause. 2017 Jul;24(7):728-753.

G10(iii): Bergendal A, Kieler H, Sundström A, Hirschberg AL, Kocoska-Maras L. Risk of venous thromboembolism associated with local and systemic use of hormone therapy in peri- and postmenopausal women and in relation to type and route of administration. Menopause. 2016 Jun;23(6):593-9.

G10(iv): Calle EE, Feigelson HS, Hildebrand JS, Teras LR, Thun MJ, Rodriguez C. Postmenopausal hormone use and breast cancer associations differ by hormone regimen and histologic subtype. Cancer 2009; 115(5): 936-45. Erratum in: Cancer 2009; 115(7): 1587.

G10(v): Diergaarde B, Potter JD, Jupe ER, Manjeshwar S, Shimasaki CD, Pugh TW, Defreese DC, Gramling BA, Evans I, White E. Polymorphisms in genes involved in sex hormone metabolism, estrogen plus progestin hormone therapy use, and risk of postmenopausal breast cancer. Cancer Epidemiol Biomarkers Prev 2008; 17(7): 1751-9.

G10(vi): O'Mahony D, O'Sullivan D, Byrne S, O'Connor MN, Ryan C, Gallagher P. STOPP/START criteria for potentially inappropriate prescribing in older people: version 2. Age Ageing. 2015 Mar;44(2):213-8.

#### G11. Oral estrogens without progestogen in patients with intact uterus (risk of endometrial cancer).

G11(i): Management of symptomatic vulvovaginal atrophy: 2013 position statement of The North American Menopause Society. Menopause. 2013 Sep;20(9):888-902; quiz 903-4.

G11(ii): Dick SE, DeWitt DE, Anawalt BD. Postmenopausal hormone replacement therapy and major clinical outcomes: a focus on cardiovascular disease, osteoporosis, dementia, and breast and endometrial neoplasia. Am J Manag Care 2002; 8(1): 95-104.

G11(iii): Furness S, Roberts H, Marjoribanks J, Lethaby A. Hormone therapy in postmenopausal women and risk of endometrial hyperplasia. Cochrane Database Syst Rev 2012 Aug 15;8:CD000402.

G11(iv): Marjoribanks J, Farquhar C, Roberts H, Lethaby A. Long term hormone therapy for perimenopausal and postmenopausal women. Cochrane Database Syst Rev 2012 Jul 11;7:CD004143.

G11(v): O'Mahony D, O'Sullivan D, Byrne S, O'Connor MN, Ryan C, Gallagher P. STOPP/START criteria for potentially inappropriate prescribing in older people: version 2. Age Ageing. 2015 Mar;44(2):213-8.

G11(vi): Lindahl SH. Reviewing the options for local estrogen treatment of vaginal atrophy. Int J Womens Health. 2014 Mar 13;6:307-12.

G11(vii): US Preventive Services Task Force, Grossman DC, Curry SJ, Owens DK, Barry MJ, Davidson KW, Doubeni CA, Epling JW Jr, Kemper AR, Krist AH, Kurth AE, Landefeld CS, Mangione CM, Phipps MG, Silverstein M, Simon MA, Tseng CW. Hormone Therapy for the Primary Prevention of Chronic Conditions in Postmenopausal Women: US Preventive Services Task Force Recommendation Statement. JAMA. 2017 Dec12;318(22):2224-2233.

#### G12. Megestrol as an appetite enhancer (minimal effect on weight, prothrombotic side effect).

G12(i): By the 2019 American Geriatrics Society Beers Criteria® Update Expert Panel. American Geriatrics Society 2019 Updated AGS Beers Criteria® for Potentially Inappropriate Medication Use in Older Adults. J Am Geriatr Soc. 2019 Apr;67(4):674-694.

G12(ii): Wen FK, Millar J, Oberst-Walsh L, Nashelsky J. Clinical Inquiry: Is megestrol acetate safe and effective for malnourished nursing home residents? J Fam Pract. 2018 Feb;67(2):112-113.

#### G13. Thyroid hormone in patients with subclinical hypothyroidism (TSH: 4-10 mIU/L; free T4: normal) (no additional benefit, risk of potential side effects such as atrial fibrillation, osteoporosis)

G13(i): Stott DJ, Rodondi N, Kearney PM, Ford I, Westendorp RGJ, Mooijaart SP, Sattar N, Aubert CE, Aujesky D, Bauer DC, Baumgartner C, Blum MR, Browne JP, Byrne S, Collet TH, Dekkers OM, den Elzen WPJ, Du Puy RS, Ellis G, Feller M, Floriani C, Hendry K, Hurley C, Jukema JW, Kean S, Kelly M, Krebs D, Langhorne P, McCarthy G, McCarthy V, McConnachie A, McDade M, Messow M, O'Flynn A, O'Riordan D, Poortvliet RKE, Quinn TJ, Russell A, Sinnott C, Smit JWA, Van Dorland HA, Walsh KA, Walsh EK, Watt T, Wilson R, Gussekloo J; TRUST Study Group. Thyroid Hormone Therapy for Older Adults with Subclinical Hypothyroidism. N Engl J Med. 2017 Jun 29;376(26):2534-2544.

G13(ii): Selmer C, Olesen JB, Hansen ML, von Kappelgaard LM, Madsen JC, Hansen PR, Pedersen OD, Faber J, Torp-Pedersen C, Gislason GH. Subclinical and overt thyroid dysfunction and risk of all-cause mortality and cardiovascular events: a large population study. J Clin Endocrinol Metab. 2014 Jul;99(7):2372-82.

G13(iii): Waring AC, Arnold AM, Newman AB, Bùzková P, Hirsch C, Cappola AR. Longitudinal changes in thyroid function in the oldest old and survival: the cardiovascular health study all-stars study. J Clin Endocrinol Metab. 2012 Nov;97(11):3944-50.

G13(iv): Garber JR, Cobin RH, Gharib H, Hennessey JV, Klein I, Mechanick JI, Pessah-Pollack R, Singer PA, Woeber KA; American Association of Clinical Endocrinologists and American Thyroid Association Taskforce on Hypothyroidism in Adults. Clinical practice guidelines for hypothyroidism in adults: cosponsored by the American Association of Clinical Endocrinologists and the American Thyroid Association. Endocr Pract. 2012 Nov Dec;18(6):988-1028. Erratum in: Endocr Pract. 2013 Jan-Feb;19(1):175.

G13(v): Razvi S, Weaver JU, Butler TJ, Pearce SH. Levothyroxine treatment of subclinical hypothyroidism, fatal and nonfatal cardiovascular events, and mortality. Arch Intern Med. 2012 May 28;172(10):811-7.

G13(vi): Pearce SH, Brabant G, Duntas LH, Monzani F, Peeters RP, Razvi S, Wemeau JL. 2013 ETA Guideline: Management of Subclinical Hypothyroidism. Eur Thyroid J. 2013 Dec;2(4):215-28.

**Section H: Antimuscarinic/anticholinergic drug burden.**

#### H1. High potency anticholinergic drugs [e.g. tricyclic antidepressants, chlorpromazine, thioridazine, clozapine, olanzapine, hyoscine, oral oxybutynin, first generation antihistamines (pheniramine, chlorpheniramine, hydroxyzine, cyproheptadine, dimenhydrinate, diphenhydramine, meclizine etc.), paroxetine] in patients with falls/ constipation/ narrow angle glaucoma/ delirium/ dementia/ urinary retention/ obstructive LUTS symptoms/ concurrent use of anticholinergic drugs. * High potency anticholinergic drugs *should preferably be avoided in older adults even if the patient has none of the above charactheristics.*

**Parenteral* use of the first-generation antihistamines are appropriate in acute allergic reactions.

H1(i): Verhamme KM, Sturkenboom MC, Stricker BH, Bosch R. Drug-induced urinary retention: incidence, management and prevention. Drug Saf 2008; 31(5):373-88.

H1(ii): Feinberg M. The problems of anticholinergic adverse effects in older patients. Drugs Aging 1993; 3(4): 335-48.

H1(iii): Gerretsen P, Pollock BG. Drugs with anticholinergic properties: a current perspective on use and safety. Expert Opin Drug Saf 2011; 10(5): 751-65.

H1(iv): Karimi S, Dharia SP, Flora DS, Slattum PW. Anticholinergic burden: clinical implications for seniors and strategies for clinicians. Consult Pharm 2012; 27(8): 564-82.

H1(v): O'Mahony D, O'Sullivan D, Byrne S, O'Connor MN, Ryan C, Gallagher P. STOPP/START criteria for potentially inappropriate prescribing in older people: version 2. Age Ageing. 2015 Mar;44(2):213-8.

H1(vi):Collamati A, Martone AM, Poscia A, Brandi V, Celi M, Marzetti E, Cherubini A, Landi F. Anticholinergic drugs and negative outcomes in the older population: from biological plausibility to clinical evidence. Aging Clin Exp Res. 2016 Feb;28(1):25-35.

H1(vii): Salahudeen MS, Duffull SB, Nishtala PS. Anticholinergic burden quantified by anticholinergic risk scales and adverse outcomes in older people: a systematic review. BMC Geriatr. 2015 Mar 25;15:31. doi: 10.1186/s12877-015-0029-9.

**Section J: Supplements criteria.**

#### J1. Gingko biloba extract in patients with increased bleeding risk (use of anticoagulants, NSAIDs, history of significant bleeding). **The risk of bleeding increases with use of ginkgo biloba with aspirin; it may be more appropriate not to use them in combination.*

J1(i): Robert B S. Clinical use of ginkgo biloba. In: UpToDate, Post, TW (Ed), UpToDate, Waltham, MA, 2019 last accessed date 29 October 2019

J1(ii): Clinton B W. Treatment and prevention of vascular dementia. In: UpToDate, Post, TW (Ed), UpToDate, Waltham, MA, 2019 last accessed date 29 October 2019

J1(iii): Bent S, Goldberg H, Padula A, Avins AL. Spontaneous bleeding associated with ginkgo biloba: a case report and systematic review of the literature: a case report and systematic review of the literature. J Gen Intern Med 2005; 20:657.

J1(iv): Rosenblatt M, Mindel J. Spontaneous hyphema associated with ingestion of Ginkgo biloba extract. N Engl J Med 1997; 336:1108.

J1(v): Rowin J, Lewis SL. Spontaneous bilateral subdural hematomas associated with chronic Ginkgo biloba ingestion. Neurology 1996; 46:1775.

J1(vi): Gilbert GJ. Ginkgo biloba. Neurology. 1997 Apr;48(4):1137.

J1(vii): Vale S. Subarachnoid haemorrhage associated with Ginkgo biloba. Lancet. 1998 Jul 4;352(9121):36.

J1(viii):Pedroso JL, Henriques Aquino CC, Escórcio Bezerra ML, Baiense RF, Suarez MM, Dutra LA, Braga-Neto P, Povoas Barsottini OG. Ginkgo biloba and cerebral bleeding: a case report and critical review. Neurologist. 2011 Mar;17(2):89-90.

#### J2. Systemic use of Hypericum perforatum (St. John’s Wort) in combination with antidepressants (the risk of serotonergic syndrome especially with SSRI) and drugs metabolized with cytochrome p450 (e.g. digoxin, theophylline, warfarin, carbamazepine, phenytoin, phenobarbital) (Hypericum perforatum (St. John’s Wort) activates cytochrome p450).

J2(i): Robert B Saper. Clinical use of St. John's wort. In: UpToDate, Poat, TW(Ed), Waltham, MA, 2019 last accessed date 29 October 2019.

J2(ii) Lantz MS, Buchalter E, Giambanco V. St. John's wort and antidepressant drug interactions in the elderly. J Geriatr Psychiatry Neurol. 1999;12(1):7.

J2(iii): Henderson L, Yue QY, Bergquist C, Gerden B, Arlett P. St John’s wort (Hypericum perforatum): drug interactions and clinical outcomes. British Journal of Clinical Pharmacology. 2002;54(4):349-356.

#### J3. Supplements with concurrent warfarin (high risk of interaction, increased risk of bleeding). **Many oral supplements (e.g. ginseng, garlic, turmeric, ginger, devil's claw, hypericum perforatum, coenzyme Q10, green tea, etc.) may increase the anticoagulant effect of warfarin and cause an increased risk of bleeding.*

J3(i): Ge B, Zhang Z, Zuo Z. Updates on the clinical evidenced herb-warfarin interactions. Evid Based Complement Alternat Med. 2014;2014:957362.

J3(ii): Holbrook AM, Pereira JA, Labiris R, McDonald H, Douketis JD, Crowther M, Wells PS. Systematic overview of warfarin and its drug and food interactions. Arch Intern Med. 2005 May 23;165(10):1095-106.

| **ABBREVIATIONS**  ACEI: Angiotensin converting enzyme inhibitors |
| --- |
| ARB: Angiotensin receptor blockers |
| BPSD: Behavioral and psychological symptoms of dementia |
| ChEIs:Acetylcholinesterase inhibitors |
| COPD: Chronic obstructive pulmonary disease |
| EF: Ejection fraction |
| eGFR: Estimated Glomerular Filtration Rate |
| FDA: Food and Drug Administration |
| GIS: Gastrointestinal system |
| H1 receptor: Histamine 1 receptor |
| INR: International Normalized Ratio |
| LUTS: Lower urinary tract symptoms |
| MI: myocardial infarction |
| NSAID: Non steroidal anti inflammatory drug |
| NYHA: New York Heart Association |
| OAC: Oral anticoagulant |
| pO2: Partial pressure of oxygen |
| PPI: Proton pump inhibitors |
| PVR: Post void residual |
| QTc: Corrected QT Interval |
| RAS: Renin angiotensin system |
| SGLT-2: Sodium-glucose cotransporter-2 |
| SNRIs: Serotonin-norepinephrine reuptake inhibitors |
| SSRIs: Selective serotonin reuptake inhibitors |
| TIA: Transient ischemic attack |
| TSH: Thyroid stimulating hormone |
