# Supplementary material for: Turkish inappropriate medication use in the elderly (TIME) criteria to improve prescribing in older adults: TIME-to-STOP/TIME-to-START
Source: Eur Geriatr Med. 2020 Mar 5;11(3):491–8. doi: 10.1007/s41999-020-00297-z (PMC7280176; doi:10.1007/s41999-020-00297-z)
Supplement: Supplementary file 6 — Supplementary file6 (DOCX 104 kb) [file 41999_2020_297_MOESM6_ESM.docx]

**Turkish Inappropriate Medication Use in the Elderly (TIME) criteria to improve prescribing in older adults: TIME to STOP/TIME to START**

**Journal name:** European Geriatric Medicine

**Gulistan Bahat**^1^**, Birkan Ilhan**^1^**,** Tugba Erdogan^1^**, Meltem Halil**^2^**, Sumru Savas**^3^**, Zekeriya Ulger**^4^**, Filiz Akyuz**^5^**, Ahmet Kaya Bilge**^6^**, Sibel Cakir**^7^**, Kutay Demirkan** ^8^**, Mustafa Erelel^9^, Kerim Guler**^10^**, Hasmet Hanagasi**^11^**, Belgin Izgi**^12^**, Ates Kadioglu**^13^**, Ayse Karan**^14^**, Isin Baral Kulaksizoglu**^7^**, Ali Mert**^15^**, Savas Ozturk**^16^**, Ilhan Satman**^17^**, Mehmet Sukru Sever**^18^**, Tufan Tukek**^10^**, Yagiz Uresin**^19^**, Onay Yalcin**^20^**, Nilufer Yesilot**^11^**, Meryem Merve Oren^21^, Mehmet Akif Karan**^1^

^1^*Istanbul University, Istanbul Medical School, Department of Internal Medicine, Division of Geriatrics, Istanbul, Turkey*

^2^ *Hacettepe University Faculty of Medicine, Department of Internal Medicine, Division of Geriatric Medicine, Ankara, Turkey.*

^3^ *Ege University Faculty of Medicine, Department of Internal Medicine, Division of Geriatrics, Izmir, Turkey.*

^4^ *Kirikkale University Medical School, Department of Internal Medicine, Kirikkale, Turkey*

^5^*Istanbul University Istanbul Medical School, Department of Internal Medicine, Division of Gastroenterology, Istanbul, Turkey*

^6^*Istanbul University Istanbul Medical School, Department of Cardiology, Istanbul, Turkey*

^7^*Istanbul University Istanbul Medical School, Department of Psychiatry, Istanbul, Turkey*

*^8^Hacettepe University Faculty of Pharmacy, Department of Clinical Pharmacy, Ankara, Turkey.*

^9^ *Istanbul University Istanbul Medical School, Department of Pulmonary Medicine, Istanbul, Turkey*

^10^*Istanbul University Istanbul Medical School, Department of Internal Medicine, Istanbul, Turkey*

^11^*Istanbul University Istanbul Medical School, Department of Neurology, Istanbul, Turkey*

*^12^Istanbul University Istanbul Medical School, Department of Ophthalmology, Istanbul, Turkey*

^13^*Istanbul University Istanbul Medical School, Department of Urology, Istanbul, Turkey*

^14^*Istanbul University Istanbul Medical School, Department of Physical Therapy and Rehabilitation, Istanbul, Turkey*

^15^*Istanbul Medipol University, Infectious Diseases and Clinical Microbiology, Faculty of Medicine, Istanbul, Turkey*

^16^*Haseki Training and Research Hospital, Department of Nephrology, Istanbul, Turkey*

^17^*Istanbul University Istanbul Medical School, Department of Internal Medicine, Division of Endocrinology, Istanbul, Turkey*

^18^*Istanbul University Istanbul Medical School, Department of Internal Medicine, Division of Nephrology, Istanbul, Turkey*

^19^*Istanbul University Istanbul Medical School, Department of Pharmacology, Istanbul, Turkey*

^20^*Istanbul University Istanbul Medical School, Department of Obstetrics and Gynecology, Istanbul, Turkey*

^21^*Istanbul University Istanbul Medical School, Department of Public Health, Istanbul, Turkey*

**Corresponding author:** Gulistan Bahat (**For Reprint**)

**Address:** Istanbul University, Istanbul Medical School, Department of Internal Medicine, Division of Geriatrics, Capa, 34390, Istanbul, Turkey

**Telephone:** + 90 212 414 20 00-33204

**Fax:** + 90 212 532 42 08

**E-mail address:**gbahatozturk@yahoo.com

**TIME-to-START Criteria with References and Explanations**

The use of this group of medications in the context of the specific criterion posseses indications and potential benefit in older adults, but can often be overlooked in clinical practice, or not prescribed due to advanced age, with no additional valid reason. Not using these drugs in the context of the criterion is considered as “potential inappropriate drug use’’. 

Clinicians should decide on all aspects of the patient, taking into account the potential benefits and harms of the drug in patient (benefit and harm balance) and the treatment goals determined in accordance with the expected life expectancy and patient / caregiver preferences. Clinicians may still find it appropriate not to use these drugs in their patients.

#### Explanations that are added to some criteria to aid in clinical use are given in italics right after the criterion in italics with the prefix *.

#### The references include references of the criteria and the explanations if present.

**Section A: Cardiovascular System criteria.**

**A1. Antiplatelet therapy (aspirin or clopidogrel) for secondary prevention in patients with documented atherosclerotic coronary artery disease (previous acute coronary syndrome/ coronary artery angioplasty or stenting/ coronary artery bypass grafting/ abdominal aortic aneurysm), documented atherosclerotic cerebrovascular disease (presence of ischemic stroke/TIA/ previous carotid endarterectomy or stenting) or symptomatic lower extremity artery disease.****Initiation of aspirin for primary cardiovascular protection is not appropriate in most cases (increased risk of intracranial and GIS bleeding, limited benefit)*

A1(i): Zuckerman IH, Yin X, Rattinger GB, Gottlieb SS, Simoni-Wastila L, Pierce SA, Huang TY, Shenolikar R, Stuart B. Effect of exposure to evidence-based pharmacotherapy on outcomes after acute myocardial infarction in older adults. J Am Geriatr Soc 2012; 60(10): 1854-61.

A1(ii): Alonso-Coello P, Bellmunt S, McGorrian C, Anand SS, Guzman R, Criqui MH, AklEA, Olav Vandvik P, Lansberg MG, Guyatt GH, Spencer FA; American College of Chest Physicians. Antithrombotic therapy in peripheral artery disease: Antithrombotic Therapy and Prevention of Thrombosis, 9th ed: American College of Chest Physicians Evidence-Based Clinical Practice Guidelines. Chest 2012; 141(2Suppl): e669S-90S.

A1(iii): Fleg JL, Aronow WS, Frishman WH. Cardiovascular drug therapy in the elderly: benefits and challenges. Nat Rev Cardiol 2011; 8(1): 13-28.

A1(iv): Vandvik PO, Lincoff AM, Gore JM, Gutterman DD, Sonnenberg FA, Alonso-Coello P,Akl EA, Lansberg MG, Guyatt GH, Spencer FA; American College of Chest Physicians. Primary and secondary prevention of cardiovascular disease: Antithrombotic Therapy and Prevention of Thrombosis, 9th ed: American College of Chest Physicians Evidence-Based Clinical Practice Guidelines. Chest 2012; 141(2Suppl): e637S-68S. Erratum in: Chest 2012; 141(4): 1129. Dosage error in article text.

A1(v): O'Mahony D, O'Sullivan D, Byrne S, O'Connor MN, Ryan C, Gallagher P. STOPP/START criteria for potentially inappropriate prescribing in older people: version 2. Age Ageing. 2015 Mar;44(2):213-8. doi: 10.1093/ageing/afu145. Epub 2014 Oct 16.

A1(vi): McNeil JJ, Wolfe R, Woods RL, Tonkin AM, Donnan GA, Nelson MR, Reid CM, Lockery JE, Kirpach B, Storey E, Shah RC, Williamson JD, Margolis KL, Ernst ME, Abhayaratna WP, Stocks N, Fitzgerald SM, Orchard SG, Trevaks RE, Beilin LJ, Johnston CI, Ryan J, Radziszewska B, Jelinek M, Malik M, Eaton CB, Brauer D, Cloud G, Wood EM, Mahady SE, Satterfield S, Grimm R, Murray AM; ASPREE Investigator Group. Effect of Aspirin on Cardiovascular Events and Bleeding in the Healthy Elderly. N Engl J Med. 2018 Oct 18;379(16):1509-1518.

A1(vii): ASCEND Study Collaborative Group, Bowman L, Mafham M, Wallendszus K, Stevens W, Buck G, Barton J, Murphy K, Aung T, Haynes R, Cox J, Murawska A, Young A, Lay M, Chen F, Sammons E, Waters E, Adler A, Bodansky J, Farmer A, McPherson R, Neil A, Simpson D, Peto R, Baigent C, Collins R, Parish S, Armitage J. Effects of Aspirin for Primary Prevention in Persons with Diabetes Mellitus. N Engl J Med. 2018 Oct 18;379(16):1529-1539.

A1(viii): Aboyans V, Ricco JB, Bartelink MEL, Björck M, Brodmann M, Cohnert T, Collet JP, Czerny M, De Carlo M, Debus S, Espinola-Klein C, Kahan T, Kownator S, Mazzolai L, Naylor AR, Roffi M, Röther J, Sprynger M, Tendera M, Tepe G, Venermo M, Vlachopoulos C, Desormais I; ESC Scientific Document Group. 2017 ESC Guidelines on the Diagnosis and Treatment of Peripheral Arterial Diseases, in collaboration with the European Society for Vascular Surgery (ESVS): Document covering atherosclerotic disease of extracranial carotid and vertebral, mesenteric, renal, upper and lower extremity arteriesEndorsed by: the European Stroke Organization (ESO)The Task Force for the Diagnosis and Treatment of Peripheral Arterial Diseases of the European Society of Cardiology (ESC) and of the European Society for Vascular Surgery (ESVS). Eur Heart J. 2018 Mar 1;39(9):763-816.

A1(ix): Authors/Task Force Members:, Piepoli MF, Hoes AW, Agewall S, Albus C, Brotons C, Catapano AL, Cooney MT, Corrà U, Cosyns B, Deaton C, Graham I, Hall MS, Hobbs FDR, Løchen ML, Löllgen H, Marques-Vidal P, Perk J, Prescott E, Redon J, Richter DJ, Sattar N, Smulders Y, Tiberi M, Bart van der Worp H, van Dis I, Verschuren WMM. 2016 European Guidelines on cardiovascular disease prevention in clinical practice: The Sixth Joint Task Force of the European Society of Cardiology and Other Societies on Cardiovascular Disease Prevention in Clinical Practice (constituted by representatives of 10 societies and by invited experts) Developed with the special contribution of the European Association for Cardiovascular Prevention & Rehabilitation (EACPR). Atherosclerosis. 2016 Sep;252:207-274.

**A2. Statin therapy for secondary prevention in patients with documented atherosclerotic coronary artery disease (previous acute coronary syndrome/ coronary artery angioplasty or stenting/ coronary artery bypass grafting/ abdominal aortic aneurysm), documented atherosclerotic cerebrovascular disease (presence of ischemic stroke/ TIA/ previous carotid endarterectomy or stenting) or peripheral arterial disease***.***Patients with a life expectancy of <2 years, terminal dementia, and > 85 years of age are less likely to benefit from statins, side effects (myopathy, liver toxicity…etc.) are more prevalent.*

**In those cases, statin treatment should be decided by informing the patient/ relatives with the shared decision-making principle.*

A2(i): Mills EJ, Wu P, Chong G, Ghement I, Singh S, Akl EA, Eyawo O, Guyatt G, Berwanger O, Briel M. Efficacy and safety of statin treatment for cardiovascular disease: a network meta-analysis of 170,255 patients from 76 randomized trials. QJM 2011; 104(2): 109-24. Review.

A2(ii): Brugts JJ, Yetgin T, Hoeks SE, Gotto AM, Shepherd J, Westendorp RG, de CraenAJ, Knopp RH, Nakamura H, Ridker P, van Domburg R, Deckers JW. The benefits of statins in people without established cardiovascular disease but with cardiovascular risk factors: meta-analysis of randomised controlled trials. BMJ 2009; 338: b2376. Review

A2(iii): Amarenco P, Labreuche J. Lipid management in the prevention of stroke: review and updated meta-analysis of statins for stroke prevention. Lancet Neurol 2009; 8(5): 453-63. Review.

A2(iv): Onder G, Landi F, Fusco D, Corsonello A, Tosato M, Battaglia M, Mastropaolo S, Settanni S, Antocicco M, Lattanzio F. Recommendations to prescribe in complex older adults: results of the CRIteria to assess appropriate Medication use among Elderly complex patients (CRIME) project. Drugs Aging. 2014 Jan;31(1):33-45. Review.

A2(v): O'Mahony D, O'Sullivan D, Byrne S, O'Connor MN, Ryan C, Gallagher P. STOPP/START criteria for potentially inappropriate prescribing in older people: version 2. Age Ageing. 2015 Mar;44(2):213-8. doi: 10.1093/ageing/afu145. Epub 2014 Oct 16. Review.

A2(vi): Aboyans V, Ricco JB, Bartelink MEL, Björck M, Brodmann M, Cohnert T, Collet JP, Czerny M, De Carlo M, Debus S, Espinola-Klein C, Kahan T, Kownator S, Mazzolai L, Naylor AR, Roffi M, Röther J, Sprynger M, Tendera M, Tepe G, Venermo M, Vlachopoulos C, Desormais I; ESC Scientific Document Group. 2017 ESC Guidelines on the Diagnosis and Treatment of Peripheral Arterial Diseases, in collaboration with the European Society for Vascular Surgery (ESVS): Document covering atherosclerotic disease of extracranial carotid and vertebral, mesenteric, renal, upper and lower extremity arteriesEndorsed by: the European Stroke Organization (ESO)The Task Force for the Diagnosis and Treatment of Peripheral Arterial Diseases of the European Society of Cardiology (ESC) and of the European Society for Vascular Surgery (ESVS). Eur Heart J. 2018 Mar 1;39(9):763-816.

A2(vii): Authors/Task Force Members:, Piepoli MF, Hoes AW, Agewall S, Albus C, Brotons C, Catapano AL, Cooney MT, Corrà U, Cosyns B, Deaton C, Graham I, Hall MS, Hobbs FDR, Løchen ML, Löllgen H, Marques-Vidal P, Perk J, Prescott E, Redon J, Richter DJ, Sattar N, Smulders Y, Tiberi M, Bart van der Worp H, van Dis I, Verschuren WMM. 2016 European Guidelines on cardiovascular disease prevention in clinical practice: The Sixth Joint Task Force of the European Society of Cardiology and Other Societies on Cardiovascular Disease Prevention in Clinical Practice (constituted by representatives of 10 societies and by invited experts) Developed with the special contribution of the European Association for Cardiovascular Prevention & Rehabilitation (EACPR). Atherosclerosis. 2016 Sep;252:207-274.

A2(viii): Fleg JL, Forman DE, Berra K, Bittner V, Blumenthal JA, Chen MA, Cheng S, Kitzman DW, Maurer MS, Rich MW, Shen WK, Williams MA, Zieman SJ; American Heart Association Committees on Older Populations and Exercise Cardiac Rehabilitation and Prevention of the Council on Clinical Cardiology, Council on Cardiovascular and Stroke Nursing, Council on Lifestyle and Cardiometabolic He. Secondary prevention of atherosclerotic cardiovascular disease in older adults: a scientific statement from the American Heart Association. Circulation. 2013 Nov 26;128(22):2422-46.

**A3. Antihypertensive therapy where systolic blood pressure consistently >160 mmHg and/or diastolic blood pressure consistently >90 mmHg.**

A3(i): Williams B, Poulter NR, Brown MJ, Davis M, McInnes GT, Potter JF, Sever PS, Thom SM; BHS guidelines working party, for the British Hypertension Society.British Hypertension Society guidelines for hypertension management 2004 (BHS-IV): summary. BMJ 2004; 328(7440):634-40. Erratum in: BMJ 2004; 328(7445): 926.

A3(ii): Papademetriou V, Farsang C, Elmfeldt D, Hofman A, Lithell H, Olofsson B, Skoog I, Trenkwalder P, Zanchetti A; Study on Cognition and Prognosis in the Elderly study group. Stroke prevention with the angiotensin II type 1-receptor blocker candesartan in elderly patients with isolated systolic hypertension: the Study on Cognition and Prognosis in the Elderly (SCOPE). J Am Coll Cardiol 2004; 44(6): 1175-80.

A3(iii): Bejan-Angoulvant T, Saadatian-Elahi M, Wright JM, Schron EB, Lindholm LH, Fagard R, Staessen JA, Gueyffier F. Treatment of hypertension in patients 80years and older: the lower the better? A meta-analysis of randomized controlled trials. J Hypertens 2010; 28(7): 1366-72.

A3(iv): George L B. Treatment of hypertension in patients with diabetes mellitus. In: UpToDate, Post, TW (Ed), UpToDate, Waltham, MA, 2019 last accessed date 23 October 2019

A3(v): Williams B, Mancia G, Spiering W, Agabiti Rosei E, Azizi M, Burnier M, Clement D, Coca A, De Simone G, Dominiczak A, Kahan T, Mahfoud F, Redon J, Ruilope L, Zanchetti A, Kerins M, Kjeldsen S, Kreutz R, Laurent S, Lip GYH, McManus R, Narkiewicz K, Ruschitzka F, Schmieder R, Shlyakhto E, Tsioufis K, Aboyans V, Desormais I. 2018 Practice guidelines for the management of arterial hypertension of the European Society of Hypertension (ESH) and the European Society of Cardiology (ESC). Blood Press. 2018 Dec;27(6):314-340.

A3(vi): Paul K. Whelton, Robert M. Carey, Wilbert S. Aronow, Donald E. Casey Jr., Karen J. Collins, Cheryl Dennison Himmelfarb, Sondra M. DePalma, Samuel Gidding, Kenneth A. Jamerson, Daniel W. Jones, Eric J. MacLaughlin, Paul Muntner, Bruce Ovbiagele, Sidney C. Smith Jr., Crystal C. Spencer, Randall S. Stafford, Sandra J. Taler, Randal J. Thomas, Kim A. Williams Sr., Jeff D. Williamson and Jackson T. Wright Jr. 2017 ACC/AHA/AAPA/ABC/ACPM/ AGS/APhA/ASH/ ASPC /NMA/PCNA Guideline for the Prevention, Detection, Evaluation, and Management of High Blood Pressure in Adults A Report of the American College of Cardiology/American Heart Association Task Force on Clinical Practice Guidelines. . J Am Coll Cardiol 2018;71:e127-e248

A3(vii): O'Mahony D, O'Sullivan D, Byrne S, O'Connor MN, Ryan C, Gallagher P. STOPP/START criteria for potentially inappropriate prescribing in older people: version 2. Age Ageing. 2015 Mar;44(2):213-8. doi: 10.1093/ageing/afu145. Epub 2014 Oct 16. Review.

**A4. OACs (vitamin K antagonists, direct thrombin inhibitors or factor Xa inhibitors) in the presence of chronic non-valvular atrial fibrillation, taking the CHA2DS2-VASc score into account***.
*A non vitamin K antagonist (NOAC) is recommended in preference to a vitamin K antagonist.*

A4(i): Hughes M, Lip GY; Guideline Development Group, National Clinical Guideline forManagement of Atrial Fibrillation in Primary and Secondary Care, NationalInstitute for Health and Clinical Excellence. Stroke and thromboembolism inatrial fibrillation: a systematic review of stroke risk factors, riskstratification schema and cost effectiveness data. Thromb Haemost 2008; 99(2): 295-304Review.

A4 (ii): Dentali F, Riva N, Crowther M, Turpie AG, Lip GY, Ageno W. Efficacy and safetyof the novel oral anticoagulants in atrial fibrillation: a systematic review and meta-analysis of the literature. Circulation 2012; 126(20): 2381-91. Review.

A4(iii): Hart RG, Pearce LA, Aguilar MI. Meta-analysis: antithrombotic therapy to prevent stroke in patients who have non-valvular atrial fibrillation. Ann Intern Med 2007; 146(12): 857-67.

A4 (iv): Aguilar MI, Hart R. Oral anticoagulants for preventing stroke in patients with non-valvular atrial fibrillation and no previous history of stroke or transient ischemic attacks. Cochrane Database of Systematic Reviews 2005, Issue 3. Art. No.: CD001927.

A4(v): Kirchhof P, Benussi S, Kotecha D, Ahlsson A, Atar D, Casadei B, Castella M, Diener HC, Heidbuchel H, Hendriks J, Hindricks G, Manolis AS, Oldgren J, Popescu BA, Schotten U, Van Putte B, Vardas P, Agewall S, Camm J, Baron Esquivias G,Budts W, Carerj S, Casselman F, Coca A, De Caterina R, Deftereos S, Dobrev D, Ferro JM, Filippatos G, Fitzsimons D, Gorenek B, Guenoun M, Hohnloser SH, Kolh P, Lip GY, Manolis A, McMurray J, Ponikowski P, Rosenhek R, Ruschitzka F, Savelieva I, Sharma S, Suwalski P, Tamargo JL, Taylor CJ, Van Gelder IC, Voors AA, Windecker S, Zamorano JL, Zeppenfeld K. 2016 ESC Guidelines for the management of atrial fibrillation developed in collaboration with EACTS. Eur J Cardiothorac Surg. 2016 Nov;50(5):e1-e88.

A4(vi): O'Mahony D, O'Sullivan D, Byrne S, O'Connor MN, Ryan C, Gallagher P. STOPP/START criteria for potentially inappropriate prescribing in older people: version 2. Age Ageing. 2015 Mar;44(2):213-8.

**A5. ACEI with systolic heart failure (EF<= 40%) or ST-elevation myocardial infarction.**

A5(i): Fleg JL, Aronow WS, Frishman WH. Cardiovascular drug therapy in the elderly: benefits and challenges. Nat Rev Cardiol 2011; 8(1):13-28. Review.

A5(ii): Arif SA, Mergenhagen KA, Del Carpio RO, Ho C. Treatment of systolic heartfailure in the elderly: an evidence-based review. Ann Pharmacother 2010; 44(10): 1604-14. Review.

A5(iii): Lahoud R, Howe M, Krishnan SM, Zacharias S, Jackson EA. Effect of use of combination evidence-based medical therapy after acute coronary syndromes on long-term outcomes. Am J Cardiol 2012; 109(2): 159-64.

A5(iv): Ibanez B, James S, Agewall S, Antunes MJ, Bucciarelli-Ducci C, Bueno H, Caforio ALP, Crea F, Goudevenos JA, Halvorsen S, Hindricks G, Kastrati A, Lenzen MJ, Prescott E, Roffi M, Valgimigli M, Varenhorst C, Vranckx P, Widimský P. [2017 ESC Guidelines for the management of acute myocardial infarction in patients presenting with ST-segment elevation.]. Kardiol Pol. 2018;76(2):229-313.

A5(v): Marco Roffi, Carlo Patrono, Jean-Philippe Collet, Christian Mueller, Marco Valgimigli, Felicita Andreotti, Jeroen J. Bax, Michael A. Borger, Carlos Brotons, Derek P. Chew, Baris Gencer, Gerd Hasenfuss, Keld Kjeldsen, Patrizio Lancellotti, Ulf Landmesser, Julinda Mehilli, Debabrata Mukherjee, Robert F. Storey, Stephan Windecker, ESC Scientific Document Group, 2015 ESC Guidelines for the management of acute coronary syndromes in patients presenting without persistent ST-segment elevation: Task Force for the Management of Acute Coronary Syndromes in Patients Presenting without Persistent ST-Segment Elevation of the European Society of Cardiology (ESC), *European Heart Journal*, Volume 37, Issue 3, 14 January 2016, Pages 267–315.

A5(vi): Task Force Members, Montalescot G, Sechtem U, Achenbach S, Andreotti F, Arden C, Budaj A, Bugiardini R, Crea F, Cuisset T, Di Mario C, Ferreira JR, Gersh BJ, Gitt AK, Hulot JS, Marx N, Opie LH, Pfisterer M, Prescott E, Ruschitzka F, Sabaté M, Senior R, Taggart DP, van der Wall EE, Vrints CJ; ESC Committee for Practice Guidelines, Zamorano JL, Achenbach S, Baumgartner H, Bax JJ, Bueno H, Dean V, Deaton C, Erol C, Fagard R, Ferrari R, Hasdai D, Hoes AW, Kirchhof P, Knuuti J, Kolh P, Lancellotti P, Linhart A, Nihoyannopoulos P, Piepoli MF, Ponikowski P, Sirnes PA, Tamargo JL, Tendera M, Torbicki A, Wijns W, Windecker S; Document Reviewers, Knuuti J, Valgimigli M, Bueno H, Claeys MJ, Donner-Banzhoff N, Erol C, Frank H, Funck-Brentano C, Gaemperli O, Gonzalez-Juanatey JR, Hamilos M, Hasdai D, Husted S, James SK, Kervinen K, Kolh P, Kristensen SD, Lancellotti P, Maggioni AP, Piepoli MF, Pries AR, Romeo F, Rydén L, Simoons ML, Sirnes PA, Steg PG, Timmis A, Wijns W, Windecker S, Yildirir A, Zamorano JL. 2013 ESC guidelines on the management of stable coronary artery disease: the Task Force on the management of stable coronary artery disease of the European Society of Cardiology. Eur Heart J. 2013 Oct;34(38):2949-3003.

A5(vii): Fihn SD, Gardin JM, Abrams J, Berra K, Blankenship JC, Dallas AP, Douglas PS, Foody JM, Gerber TC, Hinderliter AL, King SB 3rd, Kligfield PD, Krumholz HM, Kwong RY, Lim MJ, Linderbaum JA, Mack MJ, Munger MA, Prager RL, Sabik JF, Shaw LJ, Sikkema JD, Smith CR Jr, Smith SC Jr, Spertus JA, Williams SV; American College of Cardiology Foundation; American Heart Association Task Force on Practice Guidelines; American College of Physicians; American Association for Thoracic Surgery; Preventive Cardiovascular Nurses Association; Society for Cardiovascular Angiography and Interventions; Society of Thoracic Surgeons. 2012 ACCF/AHA/ACP/AATS/PCNA/SCAI/STS Guideline for the diagnosis and management of patients with stable ischemic heart disease: a report of the American College of Cardiology Foundation/American Heart Association Task Force on Practice Guidelines, and the American College of Physicians, American Association for Thoracic Surgery, Preventive Cardiovascular Nurses Association, Society for Cardiovascular Angiography and Interventions, and Society of Thoracic Surgeons. J Am Coll Cardiol. 2012 Dec 18;60(24):e44-e164.

A5(viii): O'Mahony D, O'Sullivan D, Byrne S, O'Connor MN, Ryan C, Gallagher P. STOPP/START criteria for potentially inappropriate prescribing in older people: version 2. Age Ageing. 2015 Mar;44(2):213-8. doi: 10.1093/ageing/afu145. Epub 2014 Oct 16. Review.

**A6. Beta-blocker with ischemic heart disease (antianginal effect in chronic ischemic heart disease/ mortality reduction effect in post-MI era) or systolic heart failure (EF<=%40) (bisoprolol/prolonged release metoprolol succinate/carvedilol/nebivolol in systolic heart failure; any beta blocker in ischemic heart disease)***.
*After 3 years following myocardial infarction, beta-blocker therapy may be discontinued by taking benefit and harm balance into account.*

A6(i): Theo E M. Initial pharmacologic therapy of heart failure with reduced ejection fraction in adults. Last accessed date 11 November 2019

A6(ii): Flather MD, Shibata MC, Coats AJ, et al. Randomized trial to determine the effect of nebivolol on mortality and cardiovascular hospital admission in elderly patients with heart failure (SENIORS). Eur Heart J 2005; 26:215.

A6(iii): van Veldhuisen DJ, Cohen-Solal A, Böhm M, et al. Beta-blockade with nebivolol in elderly heart failure patients with impaired and preserved left ventricular ejection fraction: Data From SENIORS (Study of Effects of Nebivolol Intervention on Outcomes and Rehospitalization in Seniors With Heart Failure). J Am Coll Cardiol 2009; 53:2150.

A6(iv): Veldhuisen DJ. Nebivolol in chronic heart failure: current evidence and future perspectives. Expert Opin Pharmacother. 2010 Apr;11(6):983-92.

A6(v): Ponikowski P, Voors AA, Anker SD, Bueno H, Cleland JGF, Coats AJS, Falk V,González-Juanatey JR, Harjola VP, Jankowska EA, Jessup M, Linde C, Nihoyannopoulos P, Parissis JT, Pieske B, Riley JP, Rosano GMC, Ruilope LM, Ruschitzka F, Rutten FH, van der Meer P; ESC Scientific Document Group. 2016 ESC Guidelines for the diagnosis and treatment of acute and chronic heart failure: The Task Force for the diagnosis and treatment of acute and chronic heart failure of the European Society of Cardiology (ESC)Developed with the special contribution of the Heart Failure Association (HFA) of the ESC. Eur Heart J. 2016 Jul 14;37(27):2129-2200.

A6(vi): Joseph P K, Julian M A, Bernard J G. Stable ischemic heart disease: Overview of care. Last accessed date 23 October 2019.

A6(vii): Smith SC Jr, Benjamin EJ, Bonow RO, Braun LT, Creager MA, Franklin BA, Gibbons RJ, Grundy SM, Hiratzka LF, Jones DW, Lloyd-Jones DM, Minissian M, Mosca L, Peterson ED, Sacco RL, Spertus J, Stein JH, Taubert KA; World Heart Federation and the Preventive Cardiovascular Nurses Association. AHA/ACCF Secondary Prevention and Risk Reduction Therapy for Patients with Coronary and other Atherosclerotic Vascular Disease: 2011 update: a guideline from the American Heart Association and American College of Cardiology Foundation. Circulation. 2011 Nov 29;124(22):2458-73.

A6(viii): O'Mahony D, O'Sullivan D, Byrne S, O'Connor MN, Ryan C, Gallagher P. STOPP/START criteria for potentially inappropriate prescribing in older people: version 2. Age Ageing. 2015 Mar;44(2):213-8.

**Section B: Central Nervous System criteria.**

#### B1. Antidepressant treatment in the presence of major depressive disorder.

B1(i): Lebowitz BD, Pearson JL, Schneider LS, Reynolds CF 3rd, Alexopoulos GS, Bruce ML, Conwell Y, Katz IR, Meyers BS, Morrison MF, Mossey J, Niederehe G, ParmeleeP. Diagnosis and treatment of depression in late life.Consensus statementupdate. JAMA 1997; 278(14): 1186-90. Review.

B1(ii): Mottram P, Wilson K, Strobl J. Antidepressants for depressed elderly. Cochrane Database Syst Rev. 2006 Jan 25;(1):CD003491. Review.

B1(iii): O'Mahony D, O'Sullivan D, Byrne S, O'Connor MN, Ryan C, Gallagher P. STOPP/START criteria for potentially inappropriate prescribing in older people: version 2. Age Ageing. 2015 Mar;44(2):213-8. doi: 10.1093/ageing/afu145. Epub 2014 Oct 16. Review.

B1(iv): Charles F. Reynolds. Evidence-Based Treatment and Prevention of Major Depressive Episodes in Later Life in Hazzards Geriatric Medicine and Gerontology Seventh edition. Eds. Halter J B, Ouslander J G, Studenski S, High K P, Asthana S, Ritchie C S, Supiano M A,; 2017 page1487-1503.

#### B2. SSRI (or SNRI or pregabalin if SSRI contraindicated) for persistent severe anxiety that interferes with functioning.  **For treatment of anxiety, if there is no accompanying depression, buspirone monotherapy may be used in patients who do not benefit from SSRIs/SNRIs or who cannot tolerate.*

B2 (i): Allgulander C, Hartford J, Russell J, Ball S, Erickson J, Raskin J, Rynn M.Pharmacotherapy of generalized anxiety disorder: results of duloxetine treatment from a pooled analysis of three clinical trials. Curr Med Res Opin 2007; 23(6): 1245-52.

B2 (ii): National Institute for Health and Clinical Excellence. Generalized anxiety disorder and panic disorder (with or without agoraphobia) in adults. Clinical Guideline 113. 2011. <http://guidance.nice.org.uk/CG113> (last accessed date 12 November 2019).

B2(iii): O'Mahony D, O'Sullivan D, Byrne S, O'Connor MN, Ryan C, Gallagher P. STOPP/START criteria for potentially inappropriate prescribing in older people: version 2. Age Ageing. 2015 Mar;44(2):213-8. doi: 10.1093/ageing/afu145. Epub 2014 Oct 16. Review.

B2(iv): Bystritsky A. Pharmacotherapy for generalized anxiety disorder in adults. In: UpToDate, Post, TW (Ed), UpToDate, Waltham, MA, 2019 last accesed date 11 November 2019

B2(v): Brawman-Mintzer O, Knapp RG, Rynn M, et al. Sertraline treatment for generalized anxiety disorder: a randomized, double-blind, placebo-controlled study. J Clin Psychiatry 2006; 67:874.

B2(vi): Dahl AA, Ravindran A, Allgulander C, et al. Sertraline in generalized anxiety disorder: efficacy in treating the psychic and somatic anxiety factors. Acta Psychiatr Scand 2005; 111:429.

B2(vii): Davidson JR, Bose A, Korotzer A, Zheng H. Escitalopram in the treatment of generalized anxiety disorder: double-blind, placebo controlled, flexible-dose study. Depress Anxiety 2004; 19:234.

B2(viii): Davidson JR, Bose A, Wang Q. Safety and efficacy of escitalopram in the long-term treatment of generalized anxiety disorder. J Clin Psychiatry 2005; 66:1441.

B2(ix): Goodman WK, Bose A, Wang Q. Treatment of generalized anxiety disorder with escitalopram: pooled results from double-blind, placebo-controlled trials. J Affect Disord 2005; 87:161.

B2(x): Craske M, Bystritsky A. Approach to treating generalized anxiety disorder in adults. In: UpToDate, Post, TW (Ed), UpToDate, Waltham, MA, 2019 last accessed date 11 November 2019

B2(xi): Baldwin DS, Anderson IM, Nutt DJ, Allgulander C, Bandelow B, den Boer JA, Christmas DM, Davies S, Fineberg N, Lidbetter N, Malizia A, McCrone P, Nabarro D, O'Neill C, Scott J, van der Wee N, Wittchen HU. Evidence-based pharmacological treatment of anxiety disorders, post-traumatic stress disorder and obsessive-compulsive disorder: a revision of the 2005 guidelines from the British Association for Psychopharmacology. J Psychopharmacol. 2014 May;28(5):403-39.

B2(xii): Lenze EJ, Rollman BL, Shear MK, Dew MA, Pollock BG, Ciliberti C, Costantino M, Snyder S, Shi P, Spitznagel E, Andreescu C, Butters MA, Reynolds CF 3rd. Escitalopram for older adults with generalized anxiety disorder: a randomized controlled trial. JAMA. 2009 Jan 21;301(3):295-303.

B2(xiii): Andreescu C, Varon D. New research on anxiety disorders in the elderly and an update on evidence-based treatments. Curr Psychiatry Rep. 2015 Jul;17(7):53.

B2(xiv): Daniel D. Sewell, Steve Koh, Jeanne Maglione, Ryan Greytak, Laura Marrone, Dilip V. Jeste. General Topics in Geriatric Psychiatry, ANXIETY DISORDERS. in Hazzards Geriatric Medicine and Gerontology Seventh edition. Eds. Halter J B, Ouslander J G, Studenski S, High K P, Asthana S, Ritchie C S, Supiano M A,; 2017 pages 1532-36.

#### B3. Acetylcholinesterase inhibitors for mild-moderate Alzheimer’s disease.  **There is evidence for initiation of ChEI therapy in Alzheimer's disease.* **There is evidence for all three cholinesterase inhibitors (donepezil, galantamine, rivastigmine) in mild-moderate Alzheimer's disease and FDA approval is available.* ** There is evidence for donepezil in late Alzheimer's disease and FDA approval is available.* ** There is evidence for initiation of rivastigmine in Parkinson's disease dementia and FDA approval is available. There are studies suggesting that donepezil may be beneficial in dementia of Parkinson's disease.* ** There is no conclusive evidence for use of ChEIs in other dementia types such as Lewy body dementia and vascular dementia; but they may be considered.* ** There are studies suggesting that donepezil and rivastigmine may be beneficial in Lewy body dementia. For Lewy body dementia, FDA approval is not present for any of the ChEIs.* ** There are studies suggesting that ChEIs may be beneficial in vascular dementia. For vascular dementia,FDA approval is not present for any of the ChEIs.*

B3(i): Raina P, Santaguida P, Ismaila A, Patterson C, Cowan D, Levine M, Booker L, Oremus M. Effectiveness of cholinesterase inhibitors and memantine for treating dementia: evidence review for a clinical practice guideline. Ann Intern Med 2008; 148(5): 379-97. Review.

B3(ii): Birks J. Cholinesterase inhibitors for Alzheimer's disease. Cochrane Database Syst Rev 2006 Jan 25;(1):CD005593. Review.

B3(iii): Rolinski M, Fox C, Maidment I, McShane R. Cholinesterase inhibitors for dementia with Lewy bodies, Parkinson's disease dementia and cognitive impairment in Parkinson's disease. Cochrane Database Syst Rev 2012 Mar 14;3:CD006504.

B3(iv): O'Mahony D, O'Sullivan D, Byrne S, O'Connor MN, Ryan C, Gallagher P. STOPP/START criteria for potentially inappropriate prescribing in older people: version 2. Age Ageing. 2015 Mar;44(2):213-8. doi: 10.1093/ageing/afu145. Epub 2014 Oct 16. Review.

B3(v): Press D, Alexander M. Cholinesterase inhibitors in the treatment of dementia. In: UpToDate, Post, TW (Ed), UpToDate, Waltham, MA, 2019 last accessed date 11 November 2019.

B3(vi):Press D, Alexander M. Treatment of dementia. In: UpToDate, Post, TW (Ed), UpToDate, Waltham, MA, 2019 last accessed date 11 November 2019

B3(vii):Farlow MR. Prognosis and treatment of dementia with Lewy bodies. In: UpToDate, Post, TW (Ed), UpToDate, Waltham, MA, 2019 last accessed date 11 November 2019

B3(viii): Efficacy of rivastigmine in dementia with Lewy bodies: a randomised, double-blind, placebo-controlled international study. McKeith I, Del Ser T, Spano P, Emre M, Wesnes K, Anand R, Cicin-Sain A, Ferrara R, Spiegel R Lancet. 2000;356(9247):2031;

B3(ix): Mori E, Ikeda M, Kosaka K, Donepezil-DLB Study Investigators. Donepezil for dementia with Lewy bodies: a randomized, placebo-controlled trial. Ann Neurol 2012; 72:41.

B3(x): Ikeda M, Mori E, Matsuo K, et al. Donepezil for dementia with Lewy bodies: a randomized, placebo-controlled, confirmatory phase III trial. Alzheimers Res Ther 2015; 7:4.

B3(xi): Emre M, Aarsland D, Albanese A, et al. Rivastigmine for dementia associated with Parkinson's disease. N Engl J Med 2004; 351:2509.;

B3(xii): Dubois B, Tolosa E, Katzenschlager R, Emre M, Lees AJ, Schumann G, Pourcher E, Gray J, Thomas G, Swartz J, Hsu T, Moline ML. Donepezil in Parkinson's disease dementia: a randomized, double-blind efficacy and safety study. Mov Disord. 2012 Sep 1;27(10):1230-8.

B3(xiii): Malouf R, Birks J. Donepezil for vascular cognitive impairment. Cochrane Database Syst Rev. 2004;(1):CD004395. Review.

B3(xiv): Erkinjuntti T, Kurz A, Gauthier S, Bullock R, Lilienfeld S, Damaraju CV. Efficacy of galantamine in probable vascular dementia and Alzheimer's disease combined with cerebrovascular disease: a randomised trial. Lancet. 2002 Apr 13;359(9314):1283-90.

B3(xv): Auchus AP, Brashear HR, Salloway S, Korczyn AD, De Deyn PP, Gassmann-Mayer C; GAL-INT-26 Study Group. Galantamine treatment of vascular dementia: a randomized trial. Neurology. 2007 Jul 31;69(5):448-58.

B3(xvi): Birks J, McGuinness B, Craig D. Rivastigmine for vascular cognitive impairment. Cochrane Database Syst Rev. 2013 May 31;(5):CD004744. doi:10.1002/14651858.CD004744.pub3. Review.

B3 (xvii): Ballard C, Sauter M, Scheltens P, He Y, Barkhof F, van Straaten EC, van der Flier WM, Hsu C, Wu S, Lane R. Efficacy, safety and tolerability of rivastigmine capsules in patients with probable vascular dementia: the VantagE study. Curr Med Res Opin. 2008 Sep;24(9):2561-74.

B3(xviii): Mok V, Wong A, Ho S, Leung T, Lam WW, Wong KS. Rivastigmine in Chinese patients with subcortical vascular dementia. Neuropsychiatr Dis Treat. 2007 Dec;3(6):943-8.

B3(xix): Narasimhalu K, Effendy S, Sim CH, Lee JM, Chen I, Hia SB, Xue HL, Corrales MP, Chang HM, Wong MC, Chen CP, Tan EK. A randomized controlled trial of rivastigmine in patients with cognitive impairment no dementia because of cerebrovascular disease. Acta Neurol Scand. 2010 Apr;121(4):217-24.

B3(xx): U.S. Food and Drug Administration. Donepezil hydrochloride: HIGHLIGHTS OF PRESCRIBING INFORMATION by FDA. Available at: <https://www.accessdata.fda.gov/drugsatfda_docs/label/2012/020690s035,021720s008,022568s005lbl.pdf> last accessed date 11 November 2019.

B3(xxi): U.S. Food and Drug Administration. Rivastigmine tartrate: HIGHLIGHTS OF PRESCRIBING INFORMATION by FDA. Available at: <https://www.accessdata.fda.gov/drugsatfda_docs/label/2006/020823s016,021025s008lbl.pdf> last accessed date 11 November 2019.

B3(xxii): U.S. Food and Drug Administration. Galantamine hydrobromide: HIGHLIGHTS OF PRESCRIBING INFORMATION by FDA. Available at: <https://www.accessdata.fda.gov/drugsatfda_docs/label/2017/021169Orig1s032,021224Orig1s030,021615Orig1s023lbl.pdf> last accessed date 11 November 2019

B3(xxiii): Birks JS, Harvey RJ. Donepezil for dementia due to Alzheimer's disease. Cochrane Database Syst Rev. 2018 Jun 18;6:CD001190.

B3(xxiv): Birks JS, Grimley Evans J. Rivastigmine for Alzheimer's disease. Cochrane Database Syst Rev. 2015 Apr 10;(4):CD001191.

#### B4. Memantine for moderate-severe Alzheimer’s disease. **Memantine may also be effective in vascular dementia.* **Memantine may be beneficial for BPSD of dementia.*

B4(i):Memantine: Drug information, Lexicomp Online. Last accessed date 22 October 2019.

B4(ii): California ADS Tx guideline; Geldmacher DS. Treatment Guidelines for Alzheimer’s Disease: Redefining Perceptions in Primary Care. Primary Care Companion to The Journal of Clinical Psychiatry. 2007;9(2):113-121

B4(iii): Press D, Alexander M. Treatment of dementia. In: UpToDate, Post, TW (Ed), UpToDate, Waltham, MA, 2019 last accessed date 11 November 2019

B4(iv): Reisberg B, Doody R, Stöffler A, Schmitt F, Ferris S, Möbius HJ; Memantine Study Group. Memantine in moderate-to-severe Alzheimer's disease. N Engl J Med. 2003 Apr 3;348(14):1333-41.

B4(v): Howard R, McShane R, Lindesay J, Ritchie C, Baldwin A, Barber R, Burns A, Dening T, Findlay D, Holmes C, Hughes A, Jacoby R, Jones R, Jones R, McKeith I, Macharouthu A, O'Brien J, Passmore P, Sheehan B, Juszczak E, Katona C, Hills R, Knapp M, Ballard C, Brown R, Banerjee S, Onions C, Griffin M, Adams J, Gray R, Johnson T, Bentham P, Phillips P. Donepezil and memantine for moderate-to-severe Alzheimer's disease. N Engl J Med. 2012 Mar 8;366(10):893-903.

B4(vi): Chen R, Chan PT, Chu H, Lin YC, Chang PC, Chen CY, Chou KR. Treatment effects between monotherapy of donepezil versus combination with memantine for Alzheimer disease: A meta-analysis. PLoS One. 2017 Aug 21;12(8):e0183586.

B4(vii): Orgogozo JM, Rigaud AS, Stöffler A, Möbius HJ, Forette F. Efficacy and safety of memantine in patients with mild to moderate vascular dementia: a randomized, placebo-controlled trial (MMM 300). Stroke. 2002 Jul;33(7):1834-9.

B4(viii): Wilcock G, Möbius HJ, Stöffler A; MMM 500 group. A double-blind, placebo-controlled multicentre study of memantine in mild to moderate vascular dementia (MMM500). Int Clin Psychopharmacol. 2002 Nov;17(6):297-305.

B4(ix): GUIDELINES FOR THE TREATMENT OF ALZHEIMER’S DISEASE. NHS Foundation Trust. Review Jan 2012. Available at: http://www.humber.nhs.uk/Downloads/Services/Pharmacy/Guidelines/Alzheimer%20disease%20treatment%20guidelines.pdf (last accessed date 11 November 2019)

B4(x): McShane  R, Westby  MJ, Roberts  E, Minakaran  N, Schneider  L, Farrimond  LE, Maayan  N, Ware  J, Debarros  J. Memantine for dementia. Cochrane Database of Systematic Reviews 2019, Issue 3. Art. No.: CD003154. DOI: 10.1002/14651858.CD003154.pub6.

#### B5. Propranolol or primidone for essential tremor that interferes with functioning. **Primidone has not FDA approval.*

#### **Side effects of primidone (sedation, vertigo and nausea) are prevalent. Initiate at the lowest available dose and increase gradually on need.*

B5(i):Zesiewicz TA, Evidence-based guideline update: treatment of essential tremor: report of the Quality Standards subcommittee of the American Academy of Neurology. Neurology. 2011 Nov 8;77(19):1752-5.

B5(ii): Reich SG. Essential Tremor. Med Clin North Am. 2019 Mar;103(2):351-356. doi: 10.1016/j.mcna.2018.10.016. Review.

B5(iii): Haubenberger D, Hallett M. Essential Tremor. N Engl J Med. 2018 May 10;378(19):1802-1810. doi: 10.1056/NEJMcp1707928. Review.

B5(iv): Primidone: Drug information, Lexicomp online. Last accessed date 12 November 2019.

#### B6. L-dopa in idiopathic Parkinson’s disease with functional impairment and disability.

B6(i): Marjama-Lyons JM, Koller WC. Parkinson's disease. Update in diagnosis and symptom management. Geriatrics 2001; 56(8): 24-5, 29-30, 33-5. Review.

B6(ii): Danisi F. Parkinson's disease. Therapeutic strategies to improve patient function and quality of life. Geriatrics 2002; 57(3): 46-50; quiz 52. Review.

B6(iii): O'Mahony D, O'Sullivan D, Byrne S, O'Connor MN, Ryan C, Gallagher P. STOPP/START criteria for potentially inappropriate prescribing in older people: version 2. Age Ageing. 2015 Mar;44(2):213-8. doi: 10.1093/ageing/afu145. Epub 2014 Oct 16. Review.

B6(iv): Ferreira JJ,Summary of the recommendations of the EFNS/MDS-ES review on therapeutic management of Parkinson's disease. Eur J Neurol. 2013 Jan;20(1):5-15

B6(v): Spindler MA, Tarsy D. Initial pharmacologic treatment of Parkinson disease. In: UpToDate, Post, TW (Ed), UpToDate, Waltham, MA, 2019 last accessed date 12 November 2019

B6(vi): Kotagal V, Bohnen NI. Parkinson Disease and Related Disorders in Hazzards Geriatric Medicine and Gerontology Seventh edition. Eds. Halter J B, Ouslander J G, Studenski S, High K P, Asthana S, Ritchie C S, Supiano M A,; 2017. Pages 1422-28.

B6(vii): Connolly BS, Lang AE. Pharmacological treatment of Parkinson disease: a review. JAMA. 2014 Apr 23-30;311(16):1670-83. doi: 10.1001/jama.2014.3654. Review.

#### B7. Addition of a MAO-B inhibitor or COMT inhibitor to L-dopa treatment when on-off motor fluctuations start in idiopathic Parkinson's disease.

B7(i): Ferreira JJ,Summary of the recommendations of the EFNS/MDS-ES review on therapeutic management of Parkinson's disease. Eur J Neurol. 2013 Jan;20(1):5-15.

B7(ii): Kotagal V, Bohnen NI. Parkinson Disease and Related Disorders in Hazzards Geriatric Medicine and Gerontology Seventh edition. Eds. Halter J B, Ouslander J G, Studenski S, High K P, Asthana S, Ritchie C S, Supiano M A,; 2017. Pages 1428-30.

B7(iii): Connolly BS, Lang AE. Pharmacological treatment of Parkinson disease: a review. JAMA. 2014 Apr 23-30;311(16):1670-83. doi: 10.1001/jama.2014.3654. Review.

#### B8. Dopamine agonists (ropinirole/pramipexole/rotigotine) or alpha-2-delta calcium channel blockers (pregabalin, gabapentin) for restless legs syndrome if the symptoms affect quality of life adversely and if iron deficiency and severe renal failure have been excluded. **L-dopa treatment (50-200 mg) may be appropriate especially in patients with intermittent symptoms. It may also be preferred in symptomatic patients with renal failure.*

B8(i): Zintzaras E, Kitsios GD, Papathanasiou AA, Konitsiotis S, Miligkos M, Rodopoulou P, Hadjigeorgiou GM. Randomized trials of dopamine agonists in restless legs syndrome: a systematic review, quality assessment, and meta-analysis. Clin Ther 2010; 32(2): 221-37. Review.

B8(ii): Hansen RA, Song L, Moore CG, Gilsenan AW, Kim MM, Calloway MO, Murray MD. Effect of ropinirole on sleep outcomes in patients with restless legs syndrome: meta-analysis of pooled individual patient data from randomized controlled trials. Pharmacotherapy 2009; 29(3): 255-62.

B8(iii): Scholz H, Trenkwalder C, Kohnen R, Riemann D, Kriston L, Hornyak M. Dopamine agonists for restless legs syndrome. Cochrane Database Syst Rev. 2011 Mar 16;(3):CD006009. doi: 10.1002/14651858.CD006009.pub2. Review.

B8(iv): Garcia-Borreguero D, Stillman P, Benes H, Buschmann H, Chaudhuri KR, Gonzalez Rodríguez VM, Högl B, Kohnen R, Monti GC, Stiasny-Kolster K, Trenkwalder C,Williams AM, Zucconi M. Algorithms for the diagnosis and treatment of restless legs syndrome in primary care. BMC Neurol. 2011 Feb 27;11:28

B8(v): O'Mahony D, O'Sullivan D, Byrne S, O'Connor MN, Ryan C, Gallagher P. STOPP/START criteria for potentially inappropriate prescribing in older people: version 2. Age Ageing. 2015 Mar;44(2):213-8. doi: 10.1093/ageing/afu145. Epub 2014 Oct 16. Review.

B8(vi): Silber MH. Treatment of restless legs syndrome and periodic limb movement disorder in adults. In: UpToDate, Post, TW (Ed), UpToDate, Waltham, MA, 2019 last accessed date 11 November 2019

B8(vii): Trenkwalder C, Stiasny K, Pollmächer T, Wetter T, Schwarz J, Kohnen R, Kazenwadel J, Krüger HP, Ramm S, Künzel M, et al. L-dopa therapy of uremic and idiopathic restless legs syndrome: a double-blind, crossover trial. Sleep. 1995Oct;18(8):681-8.

B8(viii): Garcia-Borreguero D, Silber MH, Winkelman JW, Högl B, Bainbridge J, Buchfuhrer M, Hadjigeorgiou G, Inoue Y, Manconi M, Oertel W, Ondo W, Winkelmann J, Allen RP. Guidelines for the first-line treatment of restless legs syndrome/Willis-Ekbom disease, prevention and treatment of dopaminergic augmentation: a combined task force of the IRLSSG, EURLSSG, and the RLS-foundation. Sleep Med. 2016 May;21:1-11.

B8(ix): Winkelman JW, Armstrong MJ, Allen RP, Chaudhuri KR, Ondo W, Trenkwalder C, Zee PC, Gronseth GS, Gloss D, Zesiewicz T. Practice guideline summary: Treatment of restless legs syndrome in adults: Report of the Guideline Development, Dissemination, and Implementation Subcommittee of the American Academy of Neurology. Neurology. 2016 Dec 13;87(24):2585-2593.

**Section C: Gastrointestinal System criteria.**

#### C1. Fiber supplement (psyllium, methylcellulose, polycarbophil, wheat dextrin) or polyethylene glycol for symptomatic constipation without response to lifestyle changes (diet-exercise) after excluding fecal impaction.

C1(i): Rao SSC. Constipation in the older adult.In: UpToDate, Post, TW (Ed), UpToDate, Waltham, MA, 2019 last accessed date 11 November 2019

C1(ii): O'Mahony D, O'Sullivan D, Byrne S, O'Connor MN, Ryan C, Gallagher P. STOPP/START criteria for potentially inappropriate prescribing in older people: version 2. Age Ageing. 2015 Mar;44(2):213-8. doi: 10.1093/ageing/afu145. Epub 2014 Oct 16. Review.

C1(iii): Emmanuel A, Mattace-Raso F, Neri MC, Petersen KU, Rey E, Rogers J.Constipation in older people: A consensus statement. Int J Clin Pract. 2017Jan;71(1).

C1(iv): Bharucha AE, Pemberton JH, Locke GR 3rd. American GastroenterologicalAssociation technical review on constipation. Gastroenterology. 2013Jan;144(1):218-38. doi:10.1053/j.gastro.2012.10.028. Review.

**Section D: Respiratory System criteria.**

#### D1. Regular inhaled beta2 agonist or antimuscarinic bronchodilator (e.g. ipratropium, tiotropium) for mild to moderate asthma or COPD.

D1(i): Pauwels RA, Buist AS, Ma P, Jenkins CR, Hurd SS; GOLD Scientific Committee. Global strategy for the diagnosis, management, and prevention of chronic obstructive pulmonary disease: National Heart, Lung, and Blood Institute and World Health Organization Global Initiative for Chronic Obstructive Lung Disease (GOLD): executive summary. Respir Care 2001; 46(8): 798-825. Review.

D1(ii): Keating GM. Tiotropium bromide inhalation powder: a review of its use in the management of chronic obstructive pulmonary disease. Drugs 2012; 72(2):273-300. Review.

D1(iii): Yohannes AM, Hardy CC. Treatment of chronic obstructive pulmonary disease in older patients: a practical guide. Drugs Aging 2003; 20(3): 209-28. Review.

D1(iv): McCrory DC, Brown CD. Anti-cholinergic bronchodilators versusbeta2-sympathomimetic agents for acute exacerbations of chronic obstructivepulmonary disease. Cochrane Database Syst Rev. 2002;(4):CD003900. Review.

D1(v):O'Mahony D, O'Sullivan D, Byrne S, O'Connor MN, Ryan C, Gallagher P. STOPP/START criteria for potentially inappropriate prescribing in older people: version 2. Age Ageing. 2015 Mar;44(2):213-8. doi: 10.1093/ageing/afu145. Epub 2014 Oct 16. Review.

D1(vi): Global Initiative For Chronic Obstructive Lung Disease. Global Strategy for the Diagnosis, Management, and Prevention of Chronic Obstructive Pulmonary Disease.2018 Report.Available at: <https://goldcopd.org/wp-content/uploads/2017/11/GOLD-2018-v6.0-FINAL-revised-20-Nov_WMS.pdf> lastaccessed date 23 October 2019.

D1(vii): [Anderson GP](https://www.ncbi.nlm.nih.gov/pubmed/?term=Anderson%20GP%5BAuthor%5D&cauthor=true&cauthor_uid=17085788)1.Current issues with beta2-adrenoceptor agonists: pharmacology and molecular and cellular mechanisms.[Clin Rev Allergy Immunol.](https://www.ncbi.nlm.nih.gov/pubmed/?term=anderson+gp.+current+issues) 2006 Oct-Dec;31(2-3): 119-30.

#### D2. Regular inhaled corticosteroid for moderate-severe asthma or COPD, where FEV1<50% of predicted value and repeated exacerbations requiring treatment with oral corticosteroids.

D2(i):Global Initiative For Chronic Obstructive Lung Disease. Global Strategy for the Diagnosis, Management, and Prevention of Chronic Obstructive Pulmonary Disease.2018 Report. Available at: <https://goldcopd.org/wp-content/uploads/2017/11/GOLD-2018-v6.0-FINAL-revised-20-Nov_WMS.pdf> lastaccessed date 23 October 2019.

D2(ii): O'Mahony D, O'Sullivan D, Byrne S, O'Connor MN, Ryan C, Gallagher P. STOPP/START criteria for potentially inappropriate prescribing in older people: version 2. Age Ageing. 2015 Mar;44(2):213-8. doi: 10.1093/ageing/afu145. Epub 2014 Oct 16. Review.

#### D3. Home continuous oxygen with documented chronic hypoxemia (i.e. pO2 <=55 mmHg or SaO2 <=88%)

D3(i):Tiep BL, Carter R. Long-term supplemental oxygen therapy.In: UpToDate, Post, TW (Ed), UpToDate, Waltham, MA, 2019 last accessed date 11 November 2019

D3(ii): O'Mahony D, O'Sullivan D, Byrne S, O'Connor MN, Ryan C, Gallagher P. STOPP/START criteria for potentially inappropriate prescribing in older people: version 2. Age Ageing. 2015 Mar;44(2):213-8. doi: 10.1093/ageing/afu145. Epub 2014 Oct 16. Review.

D3(iii):Croxton TL, Bailey WC. Long-term oxygen treatment in chronic obstructive pulmonary.disease: recommendations for future research: an NHLBI workshop report. Am J Respir Crit Care Med 2006; 174:373.

D3(iv): Sjöberg F, Singer M. The medical use of oxygen: a time for critical reappraisal. J Intern Med2013; 274:505.

D3(v): Global Initiative For Chronic Obstructive Lung Disease. Global Strategy for the Diagnosis, Management, and Prevention of Chronic Obstructive Pulmonary Disease.2018 Report. Available at: <https://goldcopd.org/wp-content/uploads/2017/11/GOLD-2018-v6.0-FINAL-revised-20-Nov_WMS.pdf> lastaccessed date 23 October 2019.

**Section E: Musculoskeletal System criteriaand Analgesic drugs.**

#### E1. Vitamin D if vitamin D intake <800-1000 IU per day and/or calcium if elementary calcium intake <1000-1200 mg per day.

E1(i): Cosman F, de Beur SJ, LeBoff MS, Lewiecki EM, Tanner B, Randall S, Lindsay R; National Osteoporosis Foundation. Clinician's Guide to Prevention and Treatment of Osteoporosis. Osteoporos Int. 2014 Oct;25(10):2359-81. doi:10.1007/s00198-014-2794-2. Epub 2014 Aug 15. Erratum in: Osteoporos Int. 2015 Jul;26(7):2045-7.

E1(ii):Rosen HN. Calcium and vitamin D supplementation in osteoporosis.In: UpToDate, Post, TW (Ed), UpToDate, Waltham, MA, 2019 last accessed date 11 November 2019

E1(iii): O'Mahony D, O'Sullivan D, Byrne S, O'Connor MN, Ryan C, Gallagher P. STOPP/START criteria for potentially inappropriate prescribing in older people: version 2. Age Ageing. 2015 Mar;44(2):213-8. doi: 10.1093/ageing/afu145. Epub 2014 Oct 16. Review.

E1(iv): Camacho PM, Petak SM, Binkley N, Clarke BL, Harris ST, Hurley DL, Kleerekoper M, Lewiecki EM, Miller PD, Narula HS, Pessah-Pollack R, Tangpricha V, WimalawansaSJ, Watts NB. AMERICAN ASSOCIATION OF CLINICAL ENDOCRINOLOGISTS AND AMERICANCOLLEGE OF ENDOCRINOLOGY CLINICAL PRACTICE GUIDELINES FOR THE DIAGNOSIS ANDTREATMENT OF POSTMENOPAUSAL OSTEOPOROSIS - 2016. Endocr Pract. 2016 Sep2;22(Suppl 4):1-42.

E1(v): Heflin MT. Geriatric health maintenance.In: UpToDate, Post, TW (Ed), UpToDate, Waltham, MA, 2019 last accessed date 11 November 2019

E1(vi): Stephen R. Lord. Falls.in Hazzards Geriatric Medicine and Gerontology Seventh edition. Eds. Halter J B, Ouslander J G, Studenski S, High K P, Asthana S, Ritchie C S, Supiano M A,; 2017 pages 1032-42.

E1(vii): Dennis H. Sullivan, Larry E. Johnson. Nutrition and Obesityin Hazzards Geriatric Medicine and Gerontology Seventh edition. Eds. Halter J B, Ouslander J G, Studenski S, High K P, Asthana S, Ritchie C S, Supiano M A,; 2017 page 723-24.

#### E2. Bone anti-resorptive (bisphosphonate, denosumab) or anabolic therapy (parathormone analog) in patients with documented osteoporosis [fragility fracture and/or bone mineral density T-scores (femur total, femoral neck or total lumbar)<-2.5].

**Treatment should also include adequate vitamin D and elementary calcium intake.*

E2(i): Cosman F, de Beur SJ, LeBoff MS, Lewiecki EM, Tanner B, Randall S, Lindsay R; National Osteoporosis Foundation. Clinician's Guide to Prevention and Treatment of Osteoporosis.Osteoporos Int. 2014 Oct;25(10):2359-81. doi: 10.1007/s00198-014-2794-2. Epub 2014 Aug 15. Erratum in: Osteoporos Int. 2015 Jul;26(7):2045-7.

E2(ii): O'Donnell S, Cranney A, Wells GA, Adachi JD, Reginster JY. Strontium ranelate for preventing and treating postmenopausal osteoporosis. Cochrane Database SystRev. 2006 Jul 19;(3):CD005326. Review. Update in: Cochrane Database Syst Rev.2006;(4):CD005326.

E2(iii):Rosen HN, Drezner MK. Overview of the management of osteoporosis in postmenopausal women. In: UpToDate, Post, TW (Ed), UpToDate, Waltham, MA, 2019 last accessed date 11 November 2019

E2(iv): O'Mahony D, O'Sullivan D, Byrne S, O'Connor MN, Ryan C, Gallagher P. STOPP/START criteria for potentially inappropriate prescribing in older people: version 2. Age Ageing. 2015 Mar;44(2):213-8. doi: 10.1093/ageing/afu145. Epub 2014 Oct 16. Review.

E2(v): Camacho PM, Petak SM, Binkley N, Clarke BL, Harris ST, Hurley DL, Kleerekoper M, Lewiecki EM, Miller PD, Narula HS, Pessah-Pollack R, Tangpricha V, WimalawansaSJ, Watts NB. AMERICAN ASSOCIATION OF CLINICAL ENDOCRINOLOGISTS AND AMERICANCOLLEGE OF ENDOCRINOLOGY CLINICAL PRACTICE GUIDELINES FOR THE DIAGNOSIS ANDTREATMENT OF POSTMENOPAUSAL OSTEOPOROSIS - 2016. Endocr Pract. 2016 Sep2;22(Suppl 4):1-42.

E2(vi): Eastell R, Rosen CJ, Black DM, Cheung AM, Murad MH, Shoback D. Pharmacological Management of Osteoporosis in Postmenopausal Women: An Endocrine Society*Clinical Practice Guideline. J Clin Endocrinol Metab. 2019 May 1;104(5):1595-1622.

#### E3. Bisphosphonates in patients started long-term systemic corticosteroid therapy (an anticipated duration of ≥3 months): i) if >= 7.5 mg/day prednisolone or equivalent dose is given,  ii) at any dose if T score is <-1.

**Bisphosphonate therapy may be appropriate in all patients ≥70 years of age who will receive long-term (≥3 months) corticosteroid therapy independent of dose.*

**Treatment should also include adequate vitamin D and elementary calcium intake.*

E3(i): Homik J, Cranney A, Shea B, Tugwell P, Wells G, Adachi R, Suarez-Almazor M. Bisphosphonates for steroid induced osteoporosis. Cochrane Database Syst Rev 2000; (2):CD001347. Review.

E3(ii): Iwamoto J, Takeda T, Sato Y. Effects of antifracture drugs in postmenopausal, male and glucocorticoid-induced osteoporosis--usefulness of alendronate and risedronate. Expert Opin Pharmacother 2007; 8(16): 2743-56. Review.

E3(iii): Glucocorticoid Induced Osteoporosis. Osteoporosis and metabolic bone disease diagnosis and treatment guidelines of the Society of Endocrinology and Metabolism of Turkey • 2018. Page 59-61. Available at: <http://www.temd.org.tr/admin/uploads/tbl_gruplar/20180517113533-2018-05-17tbl_gruplar113531.pdf> last accessed date 11 November 2019. (article in Turkish)

E3(iv):Allen CS, Yeung JH, Vandermeer B, Homik J. Bisphosphonates for steroid-inducedosteoporosis. Cochrane Database Syst Rev. 2016 Oct 5;10:CD001347. Review.

E3(v):Rosen HN, Saag KG.Prevention and treatment of glucocorticoid-induced osteoporosis.In: UpToDate, Post, TW (Ed), UpToDate, Waltham, MA, 2019 last accessed date 11 November 2019

E3(vi): O'Mahony D, O'Sullivan D, Byrne S, O'Connor MN, Ryan C, Gallagher P. STOPP/START criteria for potentially inappropriate prescribing in older people: version 2. Age Ageing. 2015 Mar;44(2):213-8. doi: 10.1093/ageing/afu145. Epub 2014 Oct 16. Review.

E3(vii): Compston J, Cooper A, Cooper C, Gittoes N, Gregson C, Harvey N, Hope S, Kanis JA, McCloskey EV, Poole KES, Reid DM, Selby P, Thompson F, Thurston A, Vine N; National Osteoporosis Guideline Group (NOGG). UK clinical guideline for the prevention and treatment of osteoporosis. Arch Osteoporos. 2017 Dec;12(1):43.

E3(viii): Lekamwasam S, Adachi JD, Agnusdei D, Bilezikian J, Boonen S, Borgström F, Cooper C, Diez Perez A, Eastell R, Hofbauer LC, Kanis JA, Langdahl BL, Lesnyak O, Lorenc R, McCloskey E, Messina OD, Napoli N, Obermayer-Pietsch B, Ralston SH, Sambrook PN, Silverman S, Sosa M, Stepan J, Suppan G, Wahl DA, Compston JE; Joint IOF-ECTS GIO Guidelines Working Group. A framework for the development of guidelines for the management of glucocorticoid-induced osteoporosis. Osteoporos Int. 2012 Sep;23(9):2257-76.

#### E4. Long-acting anti-resorptive treatment after discontinuation of at least two doses of denosumab (rebound increased BTMs, BMD loss and increased risk of vertebral fracture following denosumab discontinuation).

**Rebound effect is more pronounced in cases where denosumab treatment is discontinued after two doses.*

E4(i): Eastell R, Rosen CJ, Black DM, Cheung AM, Murad MH, Shoback D. Pharmacological Management of Osteoporosis in Postmenopausal Women: An Endocrine Society* Clinical Practice Guideline. J Clin Endocrinol Metab. 2019 May 1;104(5):1595-1622.

E4(ii): TsourdiE et al. Discontinuation of denosumab therapy for osteoporosis: a systematic review and position statement by ECTS. Bone2017;105:11–17.

E4(iii) Horne AM, Mihov B, Reid IR. Bone loss after romosozumab/denosumab: effects of bisphosphonatesCalcif Tissue Int 2018;103:55–61.

E4(iv): Reid IR, Horne AM, Mihov B, Gamble GD. Bone loss after denosumab: only partial protection with zoledronateCalcif Tissue Int . 2017;101:371–374.

#### E5. Antiresorptive treatment after teriparatide treatment.

E5(i): Camacho PM, Petak SM, Binkley N, Clarke BL, Harris ST, Hurley DL, Kleerekoper M, Lewiecki EM, Miller PD, Narula HS, Pessah-Pollack R, Tangpricha V, WimalawansaSJ, Watts NB. AMERICAN ASSOCIATION OF CLINICAL ENDOCRINOLOGISTS AND AMERICAN COLLEGE OF ENDOCRINOLOGY CLINICAL PRACTICE GUIDELINES FOR THE DIAGNOSIS AND TREATMENT OF POSTMENOPAUSAL OSTEOPOROSIS - 2016. Endocr Pract. 2016 Sep2;22(Suppl 4):1-42.

E5(ii): Meier C, Uebelhart B, Aubry-Rozier B, Birkhäuser M, Bischoff-Ferrari HA, Frey D, Kressig RW, Lamy O, Lippuner K, Stute P, Suhm N, Ferrari S. Osteoporosis drug treatment: duration and management after discontinuation. A position statement from the SVGO/ASCO. Swiss Med Wkly. 2017 Aug 16;147:w14484.

E5(iii): Eastell R, Rosen CJ, Black DM, Cheung AM, Murad MH, Shoback D. Pharmacological Management of Osteoporosis in Postmenopausal Women: An Endocrine Society* Clinical Practice Guideline. J Clin Endocrinol Metab. 2019 May 1;104(5):1595-1622.

#### E6. Disease-modifying anti-rheumatic drug with active, chronic rheumatoid disease.

E6(i): Saag KG, Teng GG, Patkar NM, Anuntiyo J, Finney C, Curtis JR, Paulus HE,Mudano A, Pisu M, Elkins-Melton M, Outman R, Allison JJ, Suarez Almazor M,Bridges SL Jr, Chatham WW, Hochberg M, MacLean C, Mikuls T, Moreland LW, O'DellJ, Turkiewicz AM, Furst DE; American College of Rheumatology. American College of Rheumatology 2008 recommendations for the use of nonbiologic and biologic disease-modifying antirheumatic drugs in rheumatoid arthritis. Arthritis Rheum 2008; 59(6): 762-84.

E6(ii): Köller MD, Aletaha D, Funovits J, Pangan A, Baker D, Smolen JS. Response of elderly patients with rheumatoid arthritis to methotrexate or TNF inhibitors compared with younger patients. Rheumatology (Oxford) 2009; 48(12): 1575-80.

E6(iii): Fleischmann R, Baumgartner SW, Weisman MH, Liu T, White B, Peloso P. Long term safety of etanercept in elderly subjects with rheumatic diseases. Ann Rheum Dis 2006; 65(3): 379-84.

E6(iv): O'Mahony D, O'Sullivan D, Byrne S, O'Connor MN, Ryan C, Gallagher P. STOPP/START criteria for potentially inappropriate prescribing in older people: version 2. Age Ageing. 2015 Mar;44(2):213-8. doi: 10.1093/ageing/afu145. Epub 2014 Oct 16. Review.

E6(v): Smolen JS, Landewé R, Bijlsma J, Burmester G, Chatzidionysiou K, Dougados M, Nam J, Ramiro S, Voshaar M, van Vollenhoven R, Aletaha D, Aringer M, Boers M, Buckley CD, Buttgereit F, Bykerk V, Cardiel M, Combe B, Cutolo M, van Eijk-Hustings Y, Emery P, Finckh A, Gabay C, Gomez-Reino J, Gossec L, Gottenberg JE, Hazes JMW, Huizinga T, Jani M, Karateev D, Kouloumas M, Kvien T, Li Z, Mariette X, McInnes I, Mysler E, Nash P, Pavelka K, Poór G, Richez C, van Riel P, Rubbert-Roth A, Saag K, da Silva J, Stamm T, Takeuchi T, Westhovens R, de Wit M, van der Heijde D. EULAR recommendations for the management of rheumatoid arthritis with synthetic and biological disease-modifying antirheumatic drugs: 2016 update. Ann Rheum Dis. 2017 Jun;76(6):960-977. doi: 10.1136/annrheumdis-2016-210715. Epub 2017 Mar 6. Review.

#### E7. Folic acid supplement in patients on methotrexate.

E7(i): Visser K, Katchamart W, Loza E, Martinez-Lopez JA, Salliot C, Trudeau J,Bombardier C, Carmona L, van der Heijde D, Bijlsma JW, Boumpas DT, Canhao H,Edwards CJ, Hamuryudan V, Kvien TK, Leeb BF, Martín-Mola EM, Mielants H,Müller-Ladner U, Murphy G, Østergaard M, Pereira IA, Ramos-Remus C, Valentini G, Zochling J, Dougados M. Multinational evidence-based recommendations for the use of methotrexate in rheumatic disorders with a focus on rheumatoid arthritis: integrating systematic literature research and expert opinion of a broad international panel of rheumatologists in the 3E Initiative. Ann Rheum Dis 2009; 68(7): 1086-93.

E7(ii): Ortiz Z, Shea B, Suarez Almazor M, Moher D, Wells G, Tugwell P. Folic acid and folinic acid for reducing side effects in patients receiving methotrexate for rheumatoid arthritis. Cochrane Database Syst Rev 2000; (2):CD000951. Review.

E7(iii): O'Mahony D, O'Sullivan D, Byrne S, O'Connor MN, Ryan C, Gallagher P. STOPP/START criteria for potentially inappropriate prescribing in older people: version 2. Age Ageing. 2015 Mar;44(2):213-8. doi: 10.1093/ageing/afu145. Epub 2014 Oct 16. Review.

E7(iv): Shea, B., Swinden, M. V., Ghogomu, E. T., Ortiz, Z., Katchamart, W., Rader, T., ... & Tugwell, P. (2014). Folic acid and folinic acid for reducing side effects in patients receiving methotrexate for rheumatoid arthritis.The Journal of rheumatology, 41(6), 1049-1060.

E7(v): British National Formulary vol. 76, September 2018-March 2019: p 993, 888-89.

E7(vi): Smolen JS, Landewé R, Bijlsma J, Burmester G, Chatzidionysiou K, Dougados M,Nam J, Ramiro S, Voshaar M, van Vollenhoven R, Aletaha D, Aringer M, Boers M,Buckley CD, Buttgereit F, Bykerk V, Cardiel M, Combe B, Cutolo M, vanEijk-Hustings Y, Emery P, Finckh A, Gabay C, Gomez-Reino J, Gossec L, Gottenberg JE, Hazes JMW, Huizinga T, Jani M, Karateev D, Kouloumas M, Kvien T, Li Z,Mariette X, McInnes I, Mysler E, Nash P, Pavelka K, Poór G, Richez C, van Riel P,Rubbert-Roth A, Saag K, da Silva J, Stamm T, Takeuchi T, Westhovens R, de Wit M, van der Heijde D. EULAR recommendations for the management of rheumatoidarthritis with synthetic and biological disease-modifying antirheumatic drugs:2016 update. Ann Rheum Dis. 2017 Jun;76(6):960-977. doi:10.1136/annrheumdis-2016-210715. Epub 2017 Mar 6. Review.

#### E8. Xanthine-oxidase inhibitors (primarily allopurinol) with a history of recurrent episodes of gout.

E8(i): Fravel MA, Ernst ME. Management of gout in the older adult. Am J Geriatr Pharmacother 2011; 9(5): 271-85. Review.

E8(ii): Zhang W, Doherty M, Bardin T, Pascual E, Barskova V, Conaghan P, Gerster J,Jacobs J, Leeb B, Lioté F, McCarthy G, Netter P, Nuki G, Perez-Ruiz F, Pignone A,Pimentão J, Punzi L, Roddy E, Uhlig T, Zimmermann-Gòrska I; EULAR Standing Committee for International Clinical Studies Including Therapeutics. EULAR evidence based recommendations for gout. Part II: Management. Report of a task force of the EULAR Standing Committee for International Clinical Studies Including Therapeutics (ESCISIT). Ann Rheum Dis. 2006; 65(10): 1312-24. Review.

E8(iii): Tayar JH, Lopez-Olivo MA, Suarez-Almazor ME. Febuxostat for treating chronic gout. Cochrane Database Syst Rev. 2012 Nov 14;11:CD008653. doi:10.1002/14651858.CD008653.pub2. Review.

E8(iv): O'Mahony D, O'Sullivan D, Byrne S, O'Connor MN, Ryan C, Gallagher P. STOPP/START criteria for potentially inappropriate prescribing in older people: version 2. Age Ageing. 2015 Mar;44(2):213-8. doi: 10.1093/ageing/afu145. Epub 2014 Oct 16. Review.

E8(v): British National Formulary vol. 76, September 2018-March 2019: p 1085-87.

E8(vi): Richette P, Doherty M, Pascual E, Barskova V, Becce F, Castañeda-Sanabria J,Coyfish M, Guillo S, Jansen TL, Janssens H, Lioté F, Mallen C, Nuki G, Perez-RuizF, Pimentao J, Punzi L, Pywell T, So A, Tausche AK, Uhlig T, Zavada J, Zhang W,Tubach F, Bardin T. 2016 updated EULAR evidence-based recommendations for themanagement of gout. Ann Rheum Dis. 2017 Jan;76(1):29-42. doi:10.1136/annrheumdis-2016-209707. Epub 2016 Jul 25. Review.

#### E9. High-potency opioids in moderate-severe pain, if paracetamol, NSAIDs or low-potency opioids are not adequate to the pain severity or have been ineffective.

E9(i): Papaleontiou M, Henderson CR Jr, Turner BJ, Moore AA, Olkhovskaya Y, Amanfo L,Reid MC. Outcomes associated with opioid use in the treatment of chronic non-cancer pain in older adults: a systematic review and meta-analysis. J Am Geriatr Soc 2010; 58(7): 1353-69. Review.

E9(ii): van Ojik AL, Jansen PA, Brouwers JR, van Roon EN. Treatment of chronic pain in older people: evidence-based choice of strong-acting opioids. Drugs Aging 2012; 29(8): 615-25. Review.

E9(iii):O'Mahony D, O'Sullivan D, Byrne S, O'Connor MN, Ryan C, Gallagher P. STOPP/START criteria for potentially inappropriate prescribing in older people: version 2. Age Ageing. 2015 Mar;44(2):213-8. doi: 10.1093/ageing/afu145. Epub 2014 Oct 16. Review.

E9(iv): Guerriero F. Guidance on opioids prescribing for the management of persistent non-cancer pain in older adults. World J Clin Cases. 2017 Mar 16;5(3):73-81.

E9(v): Bruce A. Ferrell. Pain Management in Hazzards Geriatric Medicine and Gerontology Seventh edition. Eds. Halter J B, Ouslander J G, Studenski S, High K P, Asthana S, Ritchie C S, Supiano M A,; 2017.

#### E10. Short-acting opioids in the presence of breakthrough pain (severe pain at intervals) for the patients with chronic pain already on long-acting opioids (risk of uncontrollable severe pain).

E10(i):Portenoy RK, Mehta Z, Ahmed E.Cancer pain management with opioids: Optimizing analgesia.In: UpToDate, Post, TW (Ed), UpToDate, Waltham, MA, 2019 last accessed date 11 November 2019

E10(ii): O'Mahony D, O'Sullivan D, Byrne S, O'Connor MN, Ryan C, Gallagher P. STOPP/START criteria for potentially inappropriate prescribing in older people: version 2. Age Ageing. 2015 Mar;44(2):213-8. doi: 10.1093/ageing/afu145. Epub 2014 Oct 16. Review.

E10(iii): John G. Cagle, Eric W. Widera. Geriatrics and Palliative Care in Current Diagnosis and Treatment: Geriatrics Second edition. Eds. Brie Williams, Anna Chang, C. Seth Landefeld, Cyrus Ahalt, Rebecca Conant, Helen Chen.; 2014 page 65.

E10(iv): Bruce A. Ferrell. Pain Management in Hazzards Geriatric Medicine and Gerontology Seventh edition. Eds. Halter J B, Ouslander J G, Studenski S, High K P, Asthana S, Ritchie C S, Supiano M A,; 2017 page 1204-1211.

**Section F: Endocrine System criteria.**

#### F1. ACEI or ARB in diabetes mellitus with proteinuria (>300 mg/day) or microalbuminuria (>30 mg/day).

#### **At the beginning of ACEI or ARB therapy, in patients with renal insufficiency, an increase in serum creatinine level is anticipated.*

#### **If serum creatinine increase is less than 30%, it is recommended to continue the treatment.*

#### **There is no absolute contraindicated baseline creatinine level to start ACEI or ARB, however, if serum creatinine> 3.0 mg/dl, it may be suggested not to start.*

#### **In patients with diabetes mellitus, serum creatinine and potassium levels should be monitored 1- 2 weeks after beginning ACEI-ARB therapy, at every dose increment and at least once a year (risk of renal detoriation and hyperpotassemia)*

F1(i):Schmidt M, Mansfield KE, Bhaskaran K, Nitsch D, Sørensen HT, Smeeth L,Tomlinson LA. Serum creatinine elevation after renin-angiotensin system blockade and long term cardiorenal risks: cohort study. BMJ. 2017 Mar 9;356:j791.

F1(ii): Bakris GL, Weir MR. Angiotensin-converting enzyme inhibitor-associated elevations in serum creatinine: is this a cause for concern? Arch Intern Med. 2000 Mar 13;160(5):685-93.

F1(iii): Bicket DP. Using ACE inhibitors appropriately. Am Fam Physician. 2002 Aug 1;66(3):461-8. Review.

F1(iv):American Geriatrics Society Expert Panel on Care of Older Adults with DiabetesMellitus, Moreno G, Mangione CM, Kimbro L, Vaisberg E. Guidelines abstracted fromthe American Geriatrics Society Guidelines for Improving the Care of Older Adultswith Diabetes Mellitus: 2013 update. J Am Geriatr Soc. 2013 Nov;61(11):2020-6.

F1(v): Lv J, Perkovic V, Foote CV, Craig ME, Craig JC, Strippoli GF. Antihypertensive agents for preventing diabetic kidney disease. Cochrane Database Syst Rev 2012 Dec 12;12:CD004136. doi: 10.1002/14651858.CD004136.pub3. Review.

F1(vi): Strippoli GF, Bonifati C, Craig M, Navaneethan SD, Craig JC. Angiotensin converting enzyme inhibitors and angiotensin II receptor antagonists for preventing the progression of diabetic kidney disease.Cochrane Database Syst Rev 2006 Oct 18;(4):CD006257. Review.

F1(vii): Blacklock CL, Hirst JA, Taylor KS, Stevens RJ, Roberts NW, Farmer AJ. Evidence for a dose effect of renin-angiotensin system inhibition on progression of microalbuminuria in Type 2 diabetes: a meta-analysis. Diabet Med 2011; 28(10): 1182-7.

F1(viii): O'Mahony D, O'Sullivan D, Byrne S, O'Connor MN, Ryan C, Gallagher P. STOPP/START criteria for potentially inappropriate prescribing in older people: version 2. Age Ageing. 2015 Mar;44(2):213-8. doi: 10.1093/ageing/afu145. Epub 2014 Oct 16. Review.

F1(ix): Pearl G. Lee, Jeffrey B. Halter. Diabetes Mellitus in Hazzards Geriatric Medicine and Gerontology Seventh edition. Eds. Halter J B, Ouslander J G, Studenski S, High K P, Asthana S, Ritchie C S, Supiano M A,; 2017 page 2309.

**Section G: Urogenital System criteria.**

#### G1. Alpha-1 receptor blocker with moderate-severe (IPSS score) LUTS, where prostatectomy is not considered necessary.

G1(i): Lowe FC. Role of the newer alpha, -adrenergic-receptor antagonists in the treatment of benign prostatic hyperplasia-related lower urinary tract symptoms. Clin Ther 2004; 26(11): 1701-13. Review.

G1(ii): Schwinn DA, Roehrborn CG. Alpha1-adrenoceptor subtypes and lower urinary tractsymptoms. Int J Urol. 2008 Mar;15(3):193-9. doi:10.1111/j.1442-2042.2007.01956.x. Review.

G1(iii): Dunn CJ, Matheson A, Faulds DM. Tamsulosin: a review of its pharmacology and therapeutic efficacy in the management of lower urinary tract symptoms. Drugs Aging 2002; 19(2):135-61. Review.

G1(iv):Cunningham GR, Kadmon D. Medical treatment of benign prostatic hyperplasia.In: UpToDate, Post, TW (Ed), UpToDate, Waltham, MA, 2019 last accessed date 11 November 2019

G1(v): O'Mahony D, O'Sullivan D, Byrne S, O'Connor MN, Ryan C, Gallagher P. STOPP/START criteria for potentially inappropriate prescribing in older people: version 2. Age Ageing. 2015 Mar;44(2):213-8. doi: 10.1093/ageing/afu145. Epub 2014 Oct 16. Review.

G1(vi):S. Gravas (Chair), J.N. Cornu, M.J. Drake, M. Gacci, C. Gratzke, T.R.W. Herrmann, S. Madersbacher, C. Mamoulakis, K.A.O. Tikkinen Guidelines Associates: M. Karavitakis, I. Kyriazis, S. Malde, V. Sakkalis, R. Umbach. EAU Guidelines on Management of Non-Neurogenic Male Lower Urinary Tract Symptoms (LUTS), incl. Benign Prostatic Obstruction (BPO).European Association of Urology 2018. Page 17-18.

#### G2. 5-alpha reductase inhibitor in addition to alpha-1 receptor blocker with moderate-severe (IPSS score) symptomatic LUTS, if the prostate volume is >30-40 ml and prostatectomy is not considered necessary.

G2(i):Cunningham GR, Kadmon D. Medical treatment of benign prostatic hyperplasia. In: UpToDate, Post, TW (Ed), UpToDate, Waltham, MA, 2019 last accessed date 11 November 2019

G2(ii): O'Mahony D, O'Sullivan D, Byrne S, O'Connor MN, Ryan C, Gallagher P. STOPP/START criteria for potentially inappropriate prescribing in older people: version 2. Age Ageing. 2015 Mar;44(2):213-8. doi: 10.1093/ageing/afu145. Epub 2014 Oct 16. Review.

G2(iii): S. Gravas (Chair), J.N. Cornu, M.J. Drake, M. Gacci, C. Gratzke, T.R.W. Herrmann, S. Madersbacher, C. Mamoulakis, K.A.O. Tikkinen Guidelines Associates: M. Karavitakis, I. Kyriazis, S. Malde, V. Sakkalis, R. Umbach. EAU Guidelines on Management of Non-Neurogenic Male Lower Urinary Tract Symptoms (LUTS), incl. Benign Prostatic Obstruction (BPO).European Association of Urology 2018. Page 18-19.

#### G3. Topical vaginal estrogen for symptomatic atrophic vaginitis after failure of non-hormonal treatments.

G3(i): Lynch C. Vaginal estrogen therapy for the treatment of atrophic vaginitis. J Womens Health (Larchmt) 2009; 18(10): 1595-606. Review.

G3(ii): Bachmann G, Bouchard C, Hoppe D, Ranganath R, Altomare C, Vieweg A, Graepel J, Helzner E. Efficacy and safety of low-dose regimens of conjugated estrogens cream administered vaginally. Menopause 2009; 16(4): 719-27.

G3(iii): Mainini G, Scaffa C, Rotondi M, Messalli EM, Quirino L, Ragucci A. Local estrogen replacement therapy in postmenopausal atrophic vaginitis: efficacy and safety of low dose 17beta-estradiol vaginal tablets. Clin Exp Obstet Gynecol 2005; 32(2): 111-3.

G3(iv): O'Mahony D, O'Sullivan D, Byrne S, O'Connor MN, Ryan C, Gallagher P. STOPP/START criteria for potentially inappropriate prescribing in older people: version 2. Age Ageing. 2015 Mar;44(2):213-8. doi: 10.1093/ageing/afu145. Epub 2014 Oct 16. Review.

G3(v):The use of vaginal estrogen in women with a history of estrogen-dependent breast cancer. Committee Opinion No. 659.American College of Obstetricians and Gynecologists. Obstet Gynecol 2016;127:e93–6.

G3(vi): The NAMS 2017 Hormone Therapy Position Statement Advisory Panel. The 2017 hormone therapy position statement of The North American Menopause Society.Menopause. 2017 Jul;24(7):728-753.

G3(vii): Bergendal A, Kieler H, Sundström A, Hirschberg AL, Kocoska-Maras L. Risk ofvenous thromboembolism associated with local and systemic use of hormone therapy in peri- and postmenopausal women and in relation to type and route ofadministration. Menopause. 2016 Jun;23(6):593-9.

G3(viii): Calle EE, Feigelson HS, Hildebrand JS, Teras LR, Thun MJ, Rodriguez C. Postmenopausal hormone use and breast cancer associations differ by hormone regimen and histologic subtype. Cancer 2009; 115(5): 936-45. Erratum in: Cancer 2009; 115(7): 1587.

G3(ix): Diergaarde B, Potter JD, Jupe ER, Manjeshwar S, Shimasaki CD, Pugh TW, Defreese DC, Gramling BA, Evans I, White E. Polymorphisms in genes involved in sex hormone metabolism, estrogen plus progestin hormone therapy use, and risk of postmenopausal breast cancer. Cancer Epidemiol Biomarkers Prev 2008; 17(7): 1751-9.

**Section H: Vaccines criteria.**

#### H1. Seasonal influenza vaccination annually.

#### **Trivalent high-dose and tetravalent influenza vaccines are more effective in the older adults than the standard-dose trivalent vaccine, and may be preferred.*

#### **Trivalent, high-dose trivalent and tetravalent influenza vaccines have FDA approval.*

H1(i):Hibberd PL. Seasonal influenza vaccination in adults. In: UpToDate, Post, TW (Ed), UpToDate, Waltham, MA, 2019 last accessed date 11November 2019

H1(ii): O'Mahony D, O'Sullivan D, Byrne S, O'Connor MN, Ryan C, Gallagher P. STOPP/START criteria for potentially inappropriate prescribing in older people: version 2. Age Ageing. 2015 Mar;44(2):213-8. doi: 10.1093/ageing/afu145. Epub 2014 Oct 16. Review.

H1(iii): U.S. Food and Drug Administration. Vaccines Licensed for Use in the United States. Content current as of: 05/09/2019. Available at:<https://www.fda.gov/vaccines-blood-biologics/vaccines/vaccines-licensed-use-united-states>(last accessed date 10November 2019)

H1(iv): Grohskopf LA, Alyanak E, Broder KR, Walter EB, Fry AM, Jernigan DB. Prevention and Control of Seasonal Influenza with Vaccines: Recommendations of the Advisory Committee on Immunization Practices — United States, 2019–20 Influenza Season. MMWR Recomm Rep 2019;68(No. RR-3):1–21.

#### H2. Pneumococcal vaccination (each one dose for 13-valent conjugate and 23-valent polysaccharide) after age 65.

#### i)in individuals who have not been previously vaccinated, 13-valent conjugate vaccine should be administered as the first dose. One year after conjugate vaccine, 23-valent polysaccharide vaccine should be applied

#### ii) in individuals who have been vaccinated by 23-valent polysaccharide vaccine previously, 13-valent conjugate vaccine should be administered one year later

#### **If the 23-valent polysaccharide vaccine was administered before the age of 65, it should be repeated after 65 years of age. Repeat vaccination should be at least 5 years after the first vaccination.* ** 23-valent polysaccharide vaccine may be repeatedatevery 10 years after 65 years.*

H2(i):Advisory Committee on Immunization Practices. Recommended Adult Immunization Schedule, United States, 2019.Centers for Disease Control and Prevention. Available at:<https://www.cdc.gov/vaccines/schedules/downloads/adult/adult-combined-schedule.pdf>.(lastaccessed date 10 November 2019)

H2(ii):An Advisory Committee Statement (ACS) National Advisory Committee on Immunization (NACI). Update on the use of 13-valent pneumococcal conjugate vaccine (PNEU-C-13) in addition to 23-valent pneumococcal polysaccharide vaccine (PNEU-P-23) in immunocompetent adults 65 years of age and older – Interim Recommendation. Date published: October 2016. Available at: <https://www.canada.ca/en/public-health/services/publications/healthy-living/update-use-of-13-valent-pneumococcal-conjugate-vaccine-pneu-c-13-in-addition-to-23-valent-pneumococcal-polysaccharide-vaccine-pneu-p-23-immunocompetent-adults-65-years-and-older-interim-recommendation.html> (lastaccessed date 10 November 2019)

H2(iii): O'Mahony D, O'Sullivan D, Byrne S, O'Connor MN, Ryan C, Gallagher P. STOPP/START criteria for potentially inappropriate prescribing in older people: version 2. Age Ageing. 2015 Mar;44(2):213-8. doi: 10.1093/ageing/afu145. Epub 2014 Oct 16. Review.

H2(iv): U.S. Food and Drug Administration. Vaccines Licensed for Use in the United States. Content current as of: 05/09/2019. Available at: <https://www.fda.gov/vaccines-blood-biologics/vaccines/vaccines-licensed-use-united-states> (last accessed date 10 November 2019)

H2(v): Heflin MT. Geriatric health maintenance.In: UpToDate, Post, TW (Ed), UpToDate, Waltham, MA, 2019 last accessed date 11 November 2019

#### H3.Vaccination for herpes zoster (reduction in risk of shingles infection and post-herpetic neuralgia).

**Vaccination is recommended to the patients including those had prior zona infection (shingles) or chickenpox (varicella).*

**RZV provides greater protection than ZVL.*

**RZV is preferred to ZVL.*

** RZV is applied intramuscularly, in two doses. The second dose should be administered 2-6 months after the first.This schedule should be used for all patients, including those with a history of prior herpes zoster and those who previously received ZVL.*

** Vaccination is recommended earliest at 6-12 months after shingles (zona infection).*

H3(i):[Oxman MN, Levin MJ, Shingles Prevention Study Group. Vaccination against Herpes Zoster and Postherpetic Neuralgia. J Infect Dis 2008; 197 Suppl 2:S228.](https://www.uptodate.com/contents/clinical-manifestations-of-varicella-zoster-virus-infection-herpes-zoster/abstract/17)

H3(ii):Albrecht MA, Levin MJ. Vaccination for the prevention of shingles (herpes zoster).In: UpToDate, Post, TW (Ed), UpToDate, Waltham, MA, 2019 last accessed date 11 November 2019

H3(iii): Oxman MN, Levin MJ, Johnson GR, Schmader KE, Straus SE, Gelb LD, Arbeit RD,Simberkoff MS, Gershon AA, Davis LE, Weinberg A, Boardman KD, Williams HM, Zhang JH, Peduzzi PN, Beisel CE, Morrison VA, Guatelli JC, Brooks PA, Kauffman CA,Pachucki CT, Neuzil KM, Betts RF, Wright PF, Griffin MR, Brunell P, Soto NE,Marques AR, Keay SK, Goodman RP, Cotton DJ, Gnann JW Jr, Loutit J, Holodniy M,Keitel WA, Crawford GE, Yeh SS, Lobo Z, Toney JF, Greenberg RN, Keller PM,Harbecke R, Hayward AR, Irwin MR, Kyriakides TC, Chan CY, Chan IS, Wang WW,Annunziato PW, Silber JL; Shingles Prevention Study Group. A vaccine to preventherpes zoster and postherpetic neuralgia in older adults. N Engl J Med. 2005 Jun 2;352(22):2271-84.

H3(iv):Heflin MT. Geriatric health maintenance.In: UpToDate, Post, TW (Ed), UpToDate, Waltham, MA, 2019 last accessed date 11 November 2019

H3(v): Kimberlin DW, Whitley RJ. Varicella-zoster vaccine for the prevention ofherpes zoster. N Engl J Med. 2007 Mar 29;356(13):1338-43. Review.

H3(vi): Curran D, Patterson BJ, Van Oorschot D, Buck PO, Carrico J, Hicks KA, Lee B,Yawn BP. Cost-effectiveness of an adjuvanted recombinant zoster vaccine in older adults in the United States who have been previously vaccinated with zostervaccine live. Hum Vaccin Immunother. 2019;15(4):765-771. doi:10.1080/21645515.2018.1558689. Epub 2019 Feb 20.

H3(vii): U.S. Food and Drug Administration. Vaccines Licensed for Use in the United States. Content current as of: 05/09/2019. Available at:<https://www.fda.gov/vaccines-blood-biologics/vaccines/vaccines-licensed-use-united-states>(last accessed date 10November 2019)

H3(viii): An Advisory Committee Statement (ACS) National Advisory Committee on Immunization (NACI). Updated Recommendations on the Use of Herpes Zoster Vaccines.Date published: 2018-08-30.

#### H4. Vaccination with Td (tetanus-diphtheria toxoid) every 10 years. **Pertussis vaccine may be recommended in older adults (such as grandfather, grandmother) who have close contact with infants younger than 1 year old. In this case, a single dose can be applied in the form of TdaP.*

H4(i): Recommended Adult Immunization Schedule for ages 19 years or older. <https://www.cdc.gov/vaccines/schedules/downloads/adult/adult-combined-schedule.pdf>. lastaccessed date 23 October 2019.

H4(ii): Ridda I, Yin JK, King C, Raina MacIntyre C, McIntyre P. The importance ofpertussis in older adults: a growing case for reviewing vaccination strategy in the elderly. Vaccine. 2012 Nov 6;30(48):6745-52.

H4(iii): [Centers for Disease Control and Prevention (CDC). Updated recommendations for use of tetanus toxoid, reduced diphtheria toxoid and acellular pertussis (Tdap) vaccine from the Advisory Committee on Immunization Practices, 2010. MMWR Morb Mortal Wkly Rep 2011; 60:13.](https://www.uptodate.com/contents/geriatric-health-maintenance/abstract/34);

H4(iv):Heflin MT. Geriatric health maintenance. In: UpToDate, Post, TW (Ed), UpToDate, Waltham, MA, 2019 last accessed date 11 November 2019

H4(v): Liang JL, Tiwari T, Moro P, Messonnier NE, Reingold A, Sawyer M, Clark TA.Prevention of Pertussis, Tetanus, and Diphtheria with Vaccines in the UnitedStates: Recommendations of the Advisory Committee on Immunization Practices(ACIP). MMWR Recomm Rep. 2018 Apr 27;67(2):1-44.

H4(vi): Diphtheria, tetanus toxoids, and acellular pertussis vaccine (DTaP and Tdap): Drug information, Lexicomp online. Lastaccessed date 11November 2019.

#### H5. Vaccination with meningococcal vaccine for patients who will pilgrimage to Mecca.  **Recommended at least 10 days before the Hajj.* **If the patient is going to be traveling again after 5 years, the dose should be repeated.*

H5(i):RecommendedAdultImmunization Schedule for ages 19 years or older. <https://www.cdc.gov/vaccines/schedules/downloads/adult/adult-combined-schedule.pdf>. last accessed date 23 October 2019.

H5(ii): David O F, Karin L. Immunizations for travel.In: UpToDate, Post, TW (Ed), UpToDate, Waltham, MA, 2019 last accessed date 10 November 2019

H5(iii): Kim DK, Riley LE, Hunter P; Advisory Committee on Immunization Practices. Recommended Immunization Schedule for Adults Aged 19 Years or Older, United States, 2018. Ann Intern Med. 2018 Feb 6;168(3):210-220.

**Section I: Supplements criteria.**

#### I1. ONS with MN or MNR if nutritional counseling/dietary supplementation are not sufficient to achieve nutritional goals. **There is more evidence for the initiation of ONS for older adults with chronic diseases.* **ONS should contain at least 400 kcal energy and 30 g of protein per day and continued for at least 1 month.*

#### **Patients receiving ONS shall be evaluated on a monthly basis.* **For guiding the treatment, energy intake of 30 kcal/kg/day is recommended.* **For healthy older adults, 1.0-1.2 g/kg/day protein intake is recommended.* **For older adults with acute or chronic disease, 1.2-1.5 g/kg/day protein intake is recommended.* **For older adults with severe illness, injury or malnutrition, higher protein intake (> 1.5 g/kg/day; up to 2.0 g/kg/day) may be required.*

I1(i):LLLnutrition Topic 8. [Approach to Oral and Enteral Nutrition in Adults](https://lllnutrition.com/course/view.php?id=13). Module 8.1. Indications, Contraindications, Complications and Monitoring of EN.  Zanetti M.Available at: <https://lllnutrition.com/mod/page/view.php?id=2654>; (last accessed date 10November 2019.)

I1(ii):Norman K, Pichard C, Lochs H, Pirlich M. Prognostic impact of disease-related malnutrition. Clin Nutr 2008; 27: 5-15.

I1(iii):Stratton RJ, Green CJ, Elia M. Disease-related malnutrition: an evidence-based approach to treatment. First Edition. CABI; First edition (January 30, 2003)

I1(iv):Guest JF, Panca M, Baeyens JP, de Man F, Ljungqvist O, Pichard C, Wait S, Wilson L. Health economic impact of managing patients following a community-based diagnosis of malnutrition in the UK. Clin Nutr. 2011; 30: 422-429.

I1(v):lllnutrition: Topic 36. Nutrition in OlderAdults. Module 36.1 Epidemiology, Aetiology and  Consequences of Malnutrition in Older Adults: Cederholm T.Available at:  <https://lllnutrition.com/mod/page/view.php?id=2685#u361p4>(last accessed date 10November 2019.)

I1(vi):Volkert D, Beck AM, Cederholm T, Cruz-Jentoft A, Goisser S, Hooper L, Kiesswetter E, Maggio M, Raynaud-Simon A, Sieber CC, Sobotka L, van Asselt D, Wirth R, Bischoff SC. ESPEN guideline on clinical nutrition and hydration in geriatrics. Clin Nutr. 2018 Jun 18.pii: S0261-5614(18)30210-3.

#### I2. ONS for hospitalized older adults with MN or MNR (increases nutrient intake and body weight, reduces the risk of complications and readmissions).

#### **Spontaneous oral energy intake of older adults with acute hospitalization is generally low and does not meet the requirements.* **It may be appropriate to continue the use of ONS in most cases after discharge from the hospital*.

I2(i): Volkert D, Beck AM, Cederholm T, Cruz-Jentoft A, Goisser S, Hooper L, Kiesswetter E, Maggio M, Raynaud-Simon A, Sieber CC, Sobotka L, van Asselt D, Wirth R, Bischoff SC. ESPEN guideline on clinical nutrition and hydration in geriatrics. Clin Nutr. 2018 Jun 18.pii: S0261-5614(18)30210-3.

#### I3. ONS for older adults with hip fractures in the postoperative period (regardless of nutritional status) (improves food intake and reduces the risk of complications). **There is no specific ONS (standard or high protein) recommendation.*

#### **It may be appropriate to continue ONS at least 1 month. In the studies, ONS was used for 1 to 6 months after hip fracture.*

#### **Preoperative initiation may also be considered.*

I3(i):Volkert D, Beck AM, Cederholm T, Cruz-Jentoft A, Goisser S, Hooper L, Kiesswetter E, Maggio M, Raynaud-Simon A, Sieber CC, Sobotka L, van Asselt D, Wirth R, Bischoff SC. ESPEN guideline on clinical nutrition and hydration in geriatrics. Clin Nutr. 2018 Jun 18.pii: S0261-5614(18)30210-3.

#### I4. ONS with pressure ulcers to ensure adequate protein and energy intake targeting 1.2-2 g/kg/day protein, 30-35 kcal/kg/day energy. ** ONS enriched with arginine, zinc, and antioxidants with a high protein and energy content may be more beneficial.* **Addition of arginine, glutamine, and HMB mayhave some positive effects.*

I4(i) Gomes F, Schuetz P, Bounoure L, Austin P, Ballesteros-Pomar M, Cederholm T, Fletcher J, Laviano A, Norman K, Poulia KA, Ravasco P, Schneider SM, Stanga Z, Weekes CE, Bischoff SC. ESPEN guidelines on nutritional support for polymorbid internal medicine patients. Clin Nutr. 2018 Feb;37(1):336-353.

I4(ii):Volkert D, Beck AM, Cederholm T, Cruz-Jentoft A, Goisser S, Hooper L, Kiesswetter E, Maggio M, Raynaud-Simon A, Sieber CC, Sobotka L, van Asselt D, Wirth R, Bischoff SC. ESPEN guideline on clinical nutrition and hydration in geriatrics. Clin Nutr. 2018 Jun 18.pii: S0261-5614(18)30210-3.

I4(iii):Stratton RJ, Ek AC, Engfer M, Moore Z, Rigby P, Wolfe R, Elia M. Enteralnutritional support in prevention and treatment of pressure ulcers: a systematic review and meta-analysis. Ageing Res Rev. 2005 Aug;4(3):422-50. Review.

I4(iv): Volkert D, Berner YN, Berry E, Cederholm T, Coti Bertrand P, Milne A, Palmblad J, Schneider S, Sobotka L, Stanga Z; DGEM (German Society for Nutritional Medicine), Lenzen-Grossimlinghaus R, Krys U, Pirlich M, Herbst B, Schütz T, Schröer W, Weinrebe W, Ockenga J, Lochs H; ESPEN (European Society for Parenteral and Enteral Nutrition). ESPEN Guidelines on Enteral Nutrition: Geriatrics. Clin Nutr. 2006 Apr;25(2):330-60.

I4(v): Cereda E, Klersy C, Serioli M, Crespi A, D'Andrea F; OligoElement Sore Trial Study Group. A nutritional formula enriched with arginine, zinc, and antioxidants for the healing of pressure ulcers: a randomized trial. Ann Intern Med. 2015 Feb 3;162(3):167-74. doi: 10.7326/M14-0696. Erratum in: Ann Intern Med. 2015 Dec 15;163(12):964.

I4(vi): Liu P, Shen WQ, Chen HL. Efficacy of arginine-enriched enteral formulas for the healing of pressure ulcers: a systematic review. J Wound Care. 2017 Jun 2;26(6):319-323.

I4(vii): Wong A, Chew A, Wang CM, Ong L, Zhang SH, Young S. The use of a specialised amino acid mixture for pressure ulcers: a placebo-controlled trial. J Wound Care. 2014 May;23(5):259-60, 262-4, 266-9.

| **ABBREVIATIONS**  ACEI: Angiotensin converting enzyme inhibitors |
| --- |
| ARB: Angiotensin receptor blockers |
| BMD: Bone mineral density |
| BPSD: Behavioral and psychological symptoms of dementia |
| BTMs: Bone Turnover Markers |
| ChEIs:Acetylcholinesterase inhibitors |
| COMT: Catechol-O-methyltransferase |
| COPD: Chronic obstructive pulmonary disease |
| EF: Ejection fraction |
| FDA: Food and Drug Administration |
| FEV1: Forced expiratory volume in 1 second |
| GIS: Gastrointestinal system |
| HMB: Beta-hydroxy beta-methylbutyrate |
| IPSS: International Prostate Symptom Score |
| LUTS: Lower urinary tract symptoms |
| MAO-B: Monoamine oxidase-B |
| MI: myocardial infarction |
| MN: Malnutrition |
| MNR: Malnutrition risk |
| NSAID: Non steroidal anti inflammatory drug |
| OAC: Oral anticoagulant |
| ONS: Oral nutritional supplements |
| pO2: Partial pressure of oxygen |
| RZV: Recombinant zoster vaccine |
| SaO2: Oxygen saturation |
| SNRIs: Serotonin-norepinephrine reuptake inhibitors |
| SSRIs: Selective serotonin reuptake inhibitors |
| TdaP: Tetanus, diphtheria, and acellular pertussis |
| TIA: Transient ischemic attack |
| ZVL: Zoster vaccine live |
